# PGAM5: A necroptosis gene associated with poor tumor prognosis that promotes cutaneous melanoma progression

**DOI:** 10.3389/fonc.2022.1004511

**Published:** 2022-11-29

**Authors:** Jianzhong Peng, Tao Wang, Chao Yue, Xianyan Luo, Peng Xiao

**Affiliations:** ^1^ Department of Dermatologic Surgery, Hangzhou Third People’s Hospital, Hangzhou, Zhejiang, China; ^2^ Sir Run Run Shaw Hospital, Zhejiang University School of Medicine, Hangzhou, China; ^3^ Institute of Immunology, Zhejiang University School of Medicine, Hangzhou, China

**Keywords:** SKCM, necrotizing apoptosis, PGAM5, prognosis, biomarker

## Abstract

Cutaneous melanoma is the deadliest type of skin cancer, and its highly aggressive and metastatic nature leads to an extremely poor prognosis. Necrotizing apoptosis, a specific form of programmed cell death, has been extensively studied in recent years. In this study, we analyzed the relationship between necroptosis-related functional genes and cutaneous melanoma in order to identify the biomarkers associated with the prognosis and progression of cutaneous melanoma. Cutaneous melanoma samples were classified into three subgroups on the basis of a necroptosis gene set. These subgroups were subjected to a prognostic survival analysis, and the greatest differences were observed between subgroups C1 and C3. Between these subgroups, 28 necrotizing apoptosis-related genes were significantly differently expressed. Among these, 16 necrotizing apoptosis-related genes were associated with cutaneous melanoma prognosis. Downscaling analysis and prognostic modeling using the least absolute shrinkage and selection operator analysis yielded nine pivotal genes and revealed phosphoglycerate translocase 5 (PGAM5) as the key gene. Then, qRT-PCR was used to verify the expression level of PGAM5. The results showed that PGAM5 was highly expressed in cutaneous melanoma tissues. In this study, a bioinformatics approach was used to identify PGAM5, a biomarker whose high expression is associated with the poor prognosis of cutaneous melanoma.

## Introduction

Melanocytes produce melanin, a pigment that help protect the skin against UV damage, and then they transfer this pigment to keratin-forming cells (1). However, when the skin is exposed to UV light or other stimuli for a long time, melanocytes may be transformed into melanoma cells due to the abnormal changes that occur at the molecular and biochemical levels (2). Cutaneous melanoma (SKCM) is the most common form of melanoma, and its incidence has steadily increased worldwide in recent years (3). Although the incidence of cutaneous melanoma is not as high as that of other cancers, the aggressive nature of cutaneous melanoma and its high mortality rate render it the most lethal type of skin cancer (4). The Global Cancer Statistics 2020 has revealed that there are over 320,000 new cutaneous melanoma cases and nearly 60,000 deaths (5). Differences in ethnic skin phenotype and in the amount of sunlight exposure, along with gender and age specificity, lead to variations in the prevalence of cutaneous melanoma across countries (6). Studies have shown that overexposure to UV radiation is the main cause of cutaneous melanoma development; in addition, the number of melanocytic nevi, genetic susceptibility, and family history are important factors contributing to the development of cutaneous melanoma (7, 8).

“Early detection, early treatment” has been emphasized in oncology treatment because most tumors can be potentially cured in early stages but show poor prognosis in late stages (9). Most newly diagnosed cutaneous melanoma cases are in their early stages; usually, these cases can be treated with surgical excision, and they are cured almost completely (10). However, the risk of recurrence remains one of the major concerns for patients with melanoma, and survival rates drop dramatically when the disease metastasizes (11). Cutaneous melanoma is a malignant neoplasm that accounts for more than 80% of all mortality in skin cancer patients (12). The highly aggressive and metastatic nature of cutaneous melanoma results in an extremely poor prognosis, with a five-year overall survival (OS) rate of not exceeding 15% for patients with advanced disease (13, 14). Therefore, the search for biomarkers associated with cutaneous melanoma progression and prognosis can help in further understanding the mechanisms of cutaneous melanoma development and progression, and it can provide useful targets for clinical treatment.

Necroptosis is a new form of programmed cell death activated by necrosomes, and it involves serine/threonine-protein kinase 1 (RIPK1) and RIPK3, as well as pseudokinase mixed-spectrum kinase structural domain-like protein (MLKL) (15). The release of cell contents into the microenvironment following necroptosis can cause a severe inflammatory response; various diseases, including certain cancers, have been found to be associated with the *in vivo* effects of necroptosis (16). Necroptosis has been speculated to play a significant regulatory role in melanoma (17). In this study, we aimed to clarify the role and function of necroptosis-related genes in the precision treatment of cutaneous melanoma through bioinformatics analysis. First, tumor samples were classified into subtypes through consensus clustering, and the core genes were identified through differential expression analysis, OS analysis, and least absolute shrinkage and selection operator (LASSO) analysis to resolve the tumor heterogeneity in necroptosis-related cutaneous melanoma. Then, *in vitro* experiments were conducted to validate the expression of the key necrotizing apoptosis-associated genes.

## Materials and methods

### Data and material sources

Clinical information corresponding to the RNA sequencing dataset for cutaneous melanoma was obtained from the The Cancer Genome Atlas (TCGA) database that includes 470 patient samples. Data on paraneoplastic skin melanoma samples were obtained from the The Genotype-Tissue Expression (GTEx) database, which includes 1809 cases. The gene sets associated with necroptosis were obtained from the Molecular Signature Database (MSigDB) (https://www.gsea-msigdb.org/gsea/msigdb/index.jsp), from which 33 genes associated with humans were screened; then, a necroptosis-related functional gene set was constructed.

### Consensus clustering

Subtype grouping of cutaneous melanoma samples was performed using the constructed necroptosis gene set, with a maximum number of clusters of 6, by using the R package ConsensusClusterPlus; the clustering heat map was analyzed by the R package pheatmap. The appropriate number of clusters by which to divide the samples was selected, and then the subgroups were subjected to a survival analysis using the R packages survival and survminer. The survival status of each subgroup was demonstrated by a KM survival curve, in which different groups were subjected to a log-rank test.

### Preliminary gene screening

The two subgroups with large differences in survival status based on the KM survival curve were compared in terms of the differential expression of necrotizing apoptotic genes. Box line plots were drawn using the R package ggplot2, and the Wilcoxon rank-sum test was employed to test the significance of gene expression between the two subgroups. Also, observation of factors with prognostic impact in the cutaneous melanoma samples was achieved using the R package SURVIVAL. One-way cox was used to analyze the genes with significant prognostic features, and forest plots were drawn using the R package forestplot to show the P-value, risk factor (HR), and 95% confidence interval (95% CI) for the top 20 significant genes. Intersecting genes were identified using Venn diagrams to select the prognostic correlates that were significantly expressed in cutaneous melanoma.

### LASSO

A dimensionality reduction analysis was performed, and prognostic models were constructed using LASSO. A LASSO-Cox regression analysis was performed using the R package glmnet with a 10-fold cross-validation. Log-rank was used in the KM survival analysis, KM curves were used to compare the survival differences between the two subgroups, and timeROC was performed to compare the predictive accuracy and risk scores of the signature genes. A one-way Cox analysis was performed for the signature genes, and forest plots were drawn using the R package forestplot to show the P-value, HR, and 95% CI for each gene; the significant genes were selected as key genes. The expression of the pivotal genes was verified in the TCGA database using the R package ggplot2 to observe their differential expression in cutaneous melanoma tissues and paracancerous tissues.

### Sample source and ethical review

The samples used in this study were obtained from Hangzhou Third People’s Hospital, and three pairs of carcinoma and adjacent samples were collected from three patients with cutaneous melanoma. This study was reviewed and approved by the Medical Ethics Review Committee of Hangzhou Third People’s Hospital (Approval No.: 2022KA032). The review process follows international ethical guidelines and relevant domestic laws and regulations.

### Quantitative reverse transcription-polymerase chain reaction

Three pairs of fresh cutaneous melanoma samples were collected for the external validation of the key genes. Total RNA was extracted from fresh cutaneous melanoma tissues using a Trizol kit (Beyotime), and cDNA was synthesized using a high-capacity cDNA reverse transcription kit (Thermo Fisher Scientific). The following primer sequences were used, with GAPDH as the endogenous control: phosphoglycerate translocase 5 (PGAM5) (forward primer: TCGTCCATTCGTCTATGACGC; reverse primer: GGCTTCCAATGAGACACGG); GAPDH (forward primer: CTGGGCTACACTGAGCACC; reverse primer: AAGTGGTCGTTGAGGGCAATG). The result is calculated by 2^-ΔΔct^.

### Statistical analysis

Data were analyzed using the SPSS software (V 17.0) and the prism software (V 6.01). A t-test was used to compare the differences between the two groups, and P<0.05 indicated statistical significance.

## Results

### Consensus clustering

**Figure 1:**
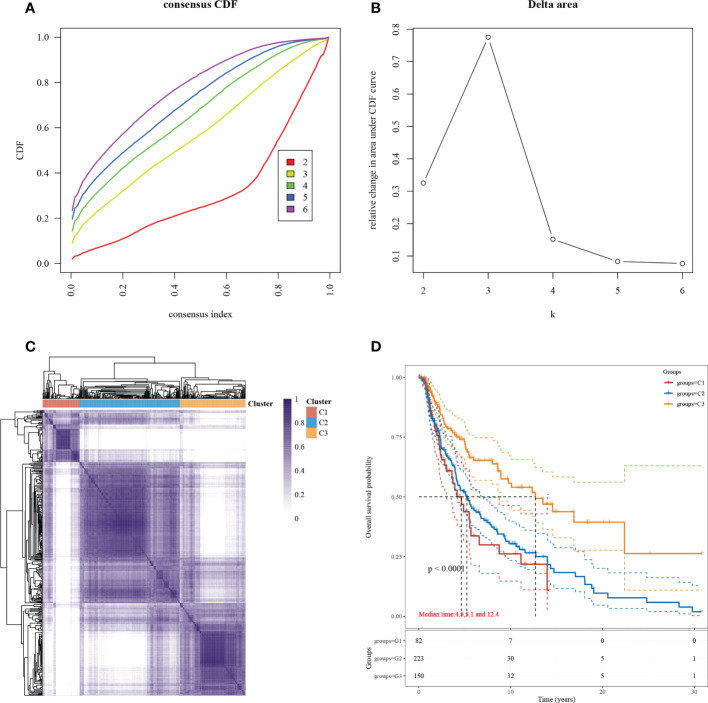
Subgroup typing based on necroptosis-associated gene sets. **(A)** CDF curve. Delta area curve for the consistent clustering and the area under the curve when samples were classified into different subtypes. **(B)** CDF delta area curve. The horizontal axis indicates a category number *k*, and the vertical axis indicates the relative change in area under the CDF curve. **(C)** A heat map showing consistent clustering results. Different colors indicate different subgroups. **(D)** KM survival curve distribution, indicating the different subgroups between prognosis comparisons between different subgroups.

Subgroup typing of cutaneous melanoma based on the set of necroptosis-related functional genes was performed through consensus clustering using similarity features, and the relative change in the area under the CDF curve was most significant when *k*=3 (**Figures 1A, B**). The samples were divided into three subgroups, each containing a different number of samples: C1 (86 cases), C2 (232 cases), and C3 (152 cases) (**Figure 1C**). To understand the differences between the three subgroups in terms of survival, we compared the prognosis based on the KM survival curves (**Figure 1D**). A significant difference in prognosis was observed, and the most significant difference was found between C1 and C3.

### Necrotizing apoptosis-related genes with prognostic significance

**Figure 2:**
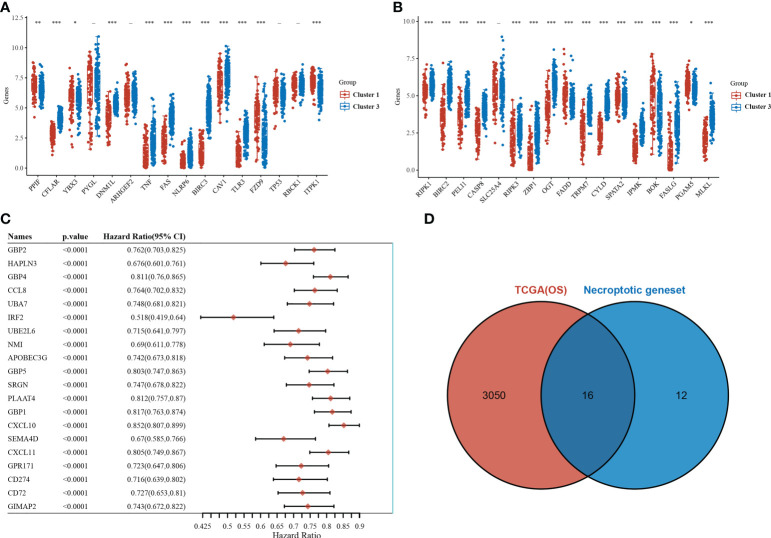
Necroptosis-related genes associated with prognosis. **(A, B)** Differential expression of necroptosis-related genes between the C1 and C3 subgroups. **(C)** Results of the whole genome survival analysis of cutaneous melanoma showing the top 20 genes. **(D)** Venn diagram. *p<0.05,**p<0.01, ***p<0.001.

Of the 33 necrotizing apoptosis-related genes, 28 genes were significantly differentially expressed between the C1 and C3 subgroups (**Figures 2A, B**). Subsequent gene-wide survival analysis of cutaneous melanoma showed that a total of 3066 genes were associated with prognosis (**Figure 2C**) (see **Supplementary Table 1** for details). When the differentially expressed necroapoptosis-related genes and the genes with prognostic significance for cutaneous melanoma were graphically represented using Venn diagrams, 16 overlapping genes were obtained (**Figure 2D**), and these genes were associated with both prognostic and necroapoptotic functions.

### PGAM5 as the pivotal gene

**Figure 3:**
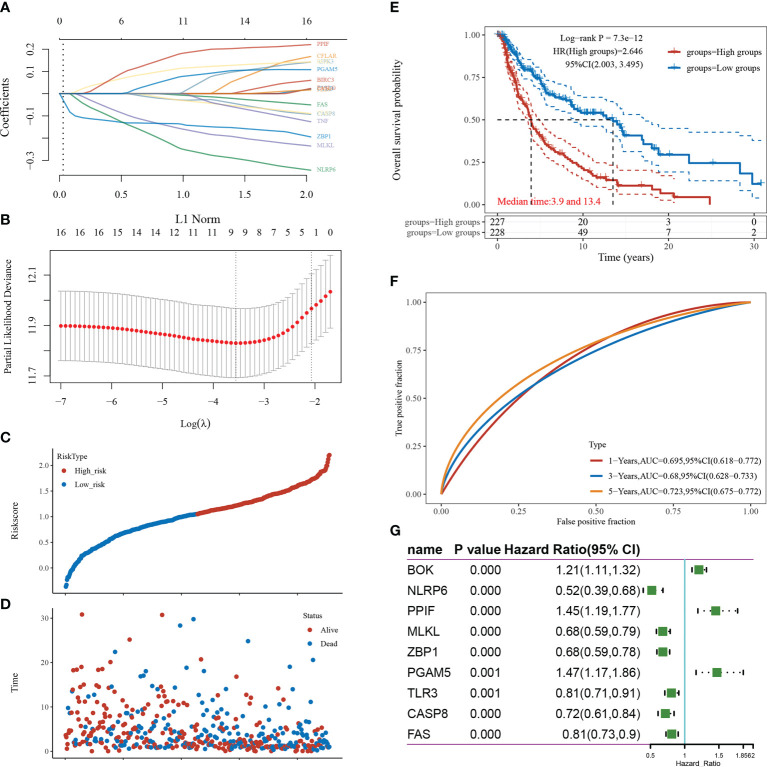
Screening of pivotal genes. **(A)** The coefficients for the selected features are presented as the λ parameter. **(B)** Partial likelihood deviation plotted against log(λ) using the LASSO-Cox regression model. **(C)** Scatter plot of risk scores from low to high. **(D)** Scatter plot distribution of survival time and survival status corresponding to the risk scores of different samples. **(E)** KM survival curve plot based on the high and low grouping of risk scores, excluding some of the missing values containing samples with missing values. **(F)** ROC curve. **(G)** Forest plot showing the P-value, HR, and 95% CI of each gene.

LASSO-based dimensionality reduction and prognostic model construction were employed to further analyze the pivotal genes associated with necroptosis in cutaneous melanoma (**Figures 3A, B**). The minimum value for the independent variable λ was 0.0285, and the risk score calculation formula was as follows: Riskscore=(-0.1351)*ZBP1+(-0.1491)*MLKL+(-0.2293)*NLRP6+(0.1086)*BOK+(-0.0261)*CASP8+(-0.004)*FAS+(0.1684)*PPIF+(-0.0248)*TLR3+(0.0672)*PGAM5.

The samples were divided into high-risk and low-risk groups according to risk score values (**Figure 3C**), and their survival times and survival status are presented in scatter plots (**Figure 3D**). The risk score value of each sample is calculated according to the risk scoring formula, and the samples are divided into high risk group and low risk group according to the median value. The KM survival curves demonstrated that survival was higher and the median survival time was longer in the low-risk score group than in the high-risk score group (**Figure 3E**). The ROC curves demonstrated the strong prognostic predictive power of the risk score model, with AUC values of 0.695, 0.68, and 0.723 at 1, 3, and 5 years, respectively (**Figure 3F**). A one-way Cox regression analysis of the genetic characteristics selected for the model was performed, and PGAM5 was selected for the follow-up study based on the results (**Figure 3G**).

### Association between high PGAM5 expression and poor prognosis in cutaneous melanoma

**Figure 4:**
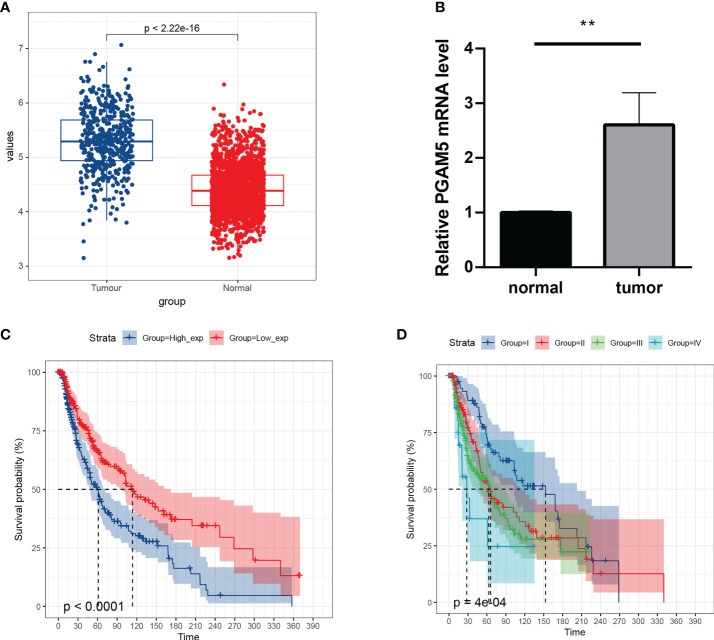
Expression and prognosis of pivotal genes. **(A)** Distribution of PGAM5 expression in cancer and paraneoplastic tissues. **(B)** Expression level of PGAM5 as determined by qRT-PCR. **(C)** KM survival curve showing the prognosis of high and low PGAM5 expression. **(D)** Prognosis of PGAM5 expression in relation to pTNM staging. **P<0.01.

To understand the expression and prognostic relationship between PGAM5 and cutaneous melanoma, we conducted an analysis using the TGCA database and GTEX database. The results showed that PGAM5 was differentially expressed in cutaneous melanoma tissues and paraneoplastic tissues and was highly expressed in tumor tissues (**Figure 4A**). PGAM5 expression in cutaneous melanoma was assessed by qRT-PCR, and the results showed that PGAM5 was overexpressed in cutaneous melanoma tissues compared with its expression in normal tissues (**Figure 4B**). The KM curves showed that high PGAM5 expression was associated with poor prognosis (**Figure 4C**); also, it showed the differences of the pTNM stages in terms of survival, wherein the survival rate decreased with higher disease stage (**Figure 4D**).

## Discussion

Unlike apoptosis and cell necrosis, necroptosis is a regulated form of necrotic cell death (18). When cells are unable to enter the apoptotic pathway, the necroptosis pathway can be activated by the stimulation of the proinflammatory cytokine tumor necrosis factor-alpha (TNF-α) (19). The loss of function of necroptosis-related genes in cutaneous melanoma mediates tumor immune escape (20), and certain compounds that effectively inhibit the proliferation of cutaneous melanoma cells in *in vitro* experiments act through the very pathway of necroptosis (21). RIPK1, a regulator of necroptosis, is known to be activated by its interaction with RIPK3, promoting the necroptotic process (22). Several genes have been identified to be involved in the necroapoptotic process; for example, RIPK3 deletion has been shown to predict the necroapoptosis resistance process in malignant melanoma (23). In this study, we conducted a series of bioinformatics analyses based on 33 human-associated necroapoptosis-related genes, aiming to identify the biomarkers related to the prognosis of cutaneous melanoma and to explore their biological functions.

Melanoma is the most aggressive type of skin cancer, and its progression and survival depend on mechanisms that favor survival, such as manipulation of cell death pathways and immune evasion by cancer cell lines (24). Necroptosis can sensitize tumor cells to anticancer drugs, and induction of this process is beneficial for killing tumor cells and is a promising tool for cancer therapy (25). Based on the expression level of the necroptosis-related gene set and the prognostic significance between this gene set and cutaneous melanoma, PGAM5 was selected as a key gene in this study. PGAM5 is a protein phosphatase that is localized in the mitochondria through its N terminus (26, 27). PGAM5 is involved in cellular activities related to the control of signal transduction pathways (28), and it can participate in apoptotic and necroptotic pathways by inducing mitochondrial autophagy after mitochondrial damage (29). The lack of translocase activity leads to the involvement of PGAM5 in the regulation of mitochondrial dynamics and programmed cell death, usually through protein–protein interactions and through a specific Ser/Thr/His protein phosphatase activity (30). Evidence has shown that PGAM5 expression is associated with necroptosis (31, 32). Research on the mechanism of necroptosis has increased in the recent years, and PGAM5 has been found to be involved in some cancers. For example, both the mRNA and protein expression of PGAM5 were much higher in lung cancer tissues than in normal tissues (33). Moreover, PGAM5 depletion inhibits hepatocellular carcinoma cell growth and promotes apoptosis (34).

In cancer, necroptosis is a double-edged sword. For some types of cancer, necroptosis occurs when apoptosis fails, thereby preventing further tumor progression; however, necroptosis can also trigger an inflammatory response that promotes cancer metastasis and immunosuppression (35, 36). Necroptosis is usually regulated by three pro-necrotic molecules, namely, RIPK1, RIPK3, and MLKL, and the use of pro-necrotic molecules for gene expression, for the analysis of prognostic impact, and for the evaluation of responsiveness to anticancer therapy has become a research hotspot in recent years (37). No direct evidence has ever established a link between PGAM5 and cutaneous melanoma. In this study, we found for the first time that PGAM5 was overexpressed in cutaneous melanoma tissues compared with its expression levels in non-tumor tissues (P<0.001) and that PGAM5 leads to a poor prognosis. In other types of tumors or diseases, PGAM5 is used as a mediator of signaling pathways, such as the RIP1/RIP3/PGAM5 pathway in breast cancer (38), the PGAM5-CypD pathway in prolactinoma (39), and the Ripk3/Pgam5 signaling pathway in septic cardiomyopathy, to induce necroptosis (40). The results of this study showed that PGAM5 was overexpressed in cutaneous melanoma and that it played an active role in tumor progression. This implies that overexpression of PGAM5, a necroptosis-associated gene, promotes cutaneous melanoma progression.

In conclusion, this study used bioinformatics analysis and *in vitro* experiments to identify the prognosis-related biomarkers for cutaneous melanoma. The results showed that PGAM5 was highly expressed in cutaneous melanoma and that it led to a poor prognosis.

## Data availability statement

The original contributions presented in the study are included in the article/**Supplementary Material**. Further inquiries can be directed to the corresponding authors.

## Author contributions

We contributed equally for this work. All authors contributed to the article and approved the submitted version.

## Funding

Hangzhou medical key discipline construction project (No [2021]21-3).

## Conflict of interest

The authors declare that the research was conducted in the absence of any commercial or financial relationships that could be construed as a potential conflict of interest.

## Publisher’s note

All claims expressed in this article are solely those of the authors and do not necessarily represent those of their affiliated organizations, or those of the publisher, the editors and the reviewers. Any product that may be evaluated in this article, or claim that may be made by its manufacturer, is not guaranteed or endorsed by the publisher.
