# Supplementary material for: PGAM5: A necroptosis gene associated with poor tumor prognosis that promotes cutaneous melanoma progression
Source: Front Oncol. 2022 Nov 29;12:1004511. doi: 10.3389/fonc.2022.1004511 (PMC9745120; doi:10.3389/fonc.2022.1004511)
Supplement: Supplementary file 1 [file Table_1.pdf]

Genes	HR	Low 95%Cl	High 95%(	cox p	log rank p
GBP2	0.761799	0.703333	0.825124	2.42E-11	6.93E-09
HAPLN3	0.676244	0.601297	0.760534	6.70E-11	1.86E-08
GBP4	0.810666	0.759769	0.864973	2.23E-10	2.63E-08
CCL8	0.764374	0.701946	0.832354	6.36E-10	6.90E-08
UBA7	0.747515	0.681021	0.820502	9.23E-10	7.61E-09
IRF2	0.517951	0.419276	0.639848	1.06E-09	3.36E-07
UBE2L6	0.714545	0.640814	0.79676	1.46E-09	1.38E-07
NMI	0.689538	0.611027	0.778137	1.67E-09	4.47E-05
APOBEC3G	0.741923	0.6731	0.817783	1.86E-09	1.80E-08
GBP5	0.802801	0.746683	0.863137	2.84E-09	6.85E-09
SRGN	0.746516	0.677857	0.822129	2.87E-09	2.51E-06
PLAAT4	0.811557	0.756986	0.870062	4.13E-09	1.64E-07
GBP1	0.816566	0.76311	0.873767	4.46E-09	6.53E-06
CXCL10	0.851577	0.807041	0.89857	4.56E-09	8.12E-07
SEMA4D	0.669832	0.585377	0.766471	5.62E-09	1.04E-06
CXCL11	0.805387	0.748544	0.866548	6.81E-09	1.80E-06
GPR171	0.722509	0.647289	0.80647	6.85E-09	1.93E-07
CD274	0.716024	0.639425	0.801799	7.19E-09	9.18E-08
CD72	0.727099	0.652615	0.810083	7.49E-09	7.86E-08
GIMAP2	0.742988	0.671763	0.821764	7.56E-09	6.14E-06
CCL4	0.780344	0.717291	0.84894	7.94E-09	1.97E-07
B2M	0.731463	0.657681	0.813522	8.20E-09	9.77E-06
CD80	0.536581	0.434032	0.66336	8.78E-09	6.82E-07
SAMSN1	0.757548	0.689137	0.832749	8.92E-09	6.00E-06
LAP3	0.66927	0.583627	0.76748	9.02E-09	8.51E-06
ADGRG5	0.666598	0.580177	0.765893	1.04E-08	1.13E-08
GCH1	0.716721	0.63934	0.803468	1.10E-08	2.58E-08
CXCL9	0.863025	0.820408	0.907855	1.19E-08	2.45E-05
APOL3	0.783668	0.720428	0.852458	1.36E-08	9.23E-07
SAMHD1	0.73994	0.66685	0.82104	1.38E-08	5.14E-07
CD38	0.771984	0.705963	0.84418	1.40E-08	2.50E-06
IDO1	0.819317	0.764794	0.877726	1.41E-08	1.68E-09
PARP12	0.728936	0.65348	0.813105	1.42E-08	4.30E-08
IRF1	0.767476	0.700183	0.841237	1.58E-08	6.13E-08
RHCG	1.202911	1.127947	1.282857	1.83E-08	0.000167
EAF2	0.662294	0.573507	0.764827	2.02E-08	3.06E-08
APOL6	0.763592	0.694893	0.839083	2.05E-08	1.96E-07
SAMD9L	0.782875	0.718654	0.852835	2.08E-08	1.09E-07
IFIH1	0.752209	0.680885	0.831005	2.12E-08	3.99E-05
PSMB9	0.779985	0.714899	0.850997	2.28E-08	1.53E-07
GIMAP7	0.774588	0.708182	0.847221	2.33E-08	1.34E-06
STAT1	0.747884	0.675317	0.828249	2.42E-08	3.39E-07
KIR2DL4	0.672415	0.584795	0.773163	2.52E-08	2.48E-07
HLA-DRB1	0.813456	0.756431	0.87478	2.58E-08	1.98E-06
HLA-DPB1	0.787266	0.723683	0.856436	2.59E-08	4.11E-07
TRIM22	0.772469	0.704932	0.846476	3.19E-08	8.94E-07
LAG3	0.814591	0.757483	0.876004	3.21E-08	2.92E-06
DDX60	0.749494	0.676557	0.830294	3.39E-08	3.82E-06
ZBP1	0.68123	0.594097	0.781143	3.86E-08	1.52E-06
CALHM6	0.817194	0.760278	0.878371	4.23E-08	3.28E-06

FCGR2A	0.748206	0.67442	0.830064	4.35E-08	7.63E-07
PARP14	0.726689	0.648164	0.814727	4.45E-08	3.66E-05
PLA2G2D	0.840373	0.789567	0.894447	4.60E-08	1.07E-07
TIGIT	0.779686	0.71297	0.852646	4.96E-08	4.69E-06
KLRD1	0.539539	0.432015	0.673825	5.29E-08	1.08E-09
IL2RA	0.752443	0.679158	0.833636	5.32E-08	3.41E-07
GCNT1	0.705384	0.621969	0.799987	5.47E-08	4.72E-07
GZMA	0.828725	0.774402	0.886858	5.60E-08	2.87E-06
CLEC2B	0.747771	0.673288	0.830493	5.65E-08	2.38E-05
IL21R	0.739327	0.662879	0.824592	5.85E-08	3.67E-08
HLA-DMB	0.793046	0.729268	0.862402	5.94E-08	1.80E-06
TBX21	0.706642	0.623201	0.801257	6.09E-08	2.29E-06
EVI2B	0.788809	0.723783	0.859676	6.50E-08	9.42E-07
NCCRP1	1.180962	1.111772	1.254457	6.68E-08	1.15E-06
TRIM69	0.67453	0.584689	0.778177	6.70E-08	8.56E-06
PTPN22	0.716715	0.634989	0.808959	6.97E-08	7.63E-08
SH2D1A	0.779859	0.712435	0.853663	7.07E-08	8.32E-08
CLEC4A	0.696625	0.610699	0.794641	7.36E-08	5.30E-06
BTN3A1	0.711575	0.628393	0.805767	8.10E-08	2.89E-05
SLA2	0.75894	0.685844	0.839826	9.38E-08	4.48E-05
IFITM1	0.808899	0.748212	0.874508	9.82E-08	6.33E-07
HLA-DQA1	0.836714	0.783494	0.89355	1.06E-07	2.42E-07
PSME1	0.572586	0.466096	0.703406	1.09E-07	0.000124
C1QA	0.81869	0.760411	0.881436	1.10E-07	7.81E-07
HLA-DMA	0.787115	0.720509	0.859878	1.12E-07	7.12E-05
CD86	0.767174	0.695502	0.846233	1.18E-07	1.35E-08
CD8A	0.835988	0.782349	0.893305	1.19E-07	5.74E-07
NCF1	0.754068	0.679252	0.837126	1.19E-07	2.92E-07
XCL2	0.770429	0.699521	0.848524	1.19E-07	9.36E-08
TLR2	0.752539	0.677289	0.83615	1.23E-07	1.07E-05
IL18BP	0.743324	0.665672	0.830035	1.37E-07	2.66E-05
CD2	0.83803	0.784659	0.895032	1.42E-07	2.47E-06
CXCR6	0.763028	0.689827	0.843997	1.47E-07	1.11E-06
LILRB1	0.761149	0.68748	0.842711	1.48E-07	6.29E-07
FCRL6	0.664372	0.570326	0.773926	1.51E-07	9.53E-08
NLRC5	0.737654	0.658392	0.826459	1.55E-07	3.06E-07
CCR5	0.796491	0.731444	0.867321	1.65E-07	2.89E-06
STAT4	0.678606	0.586903	0.784638	1.66E-07	7.30E-09
IKZF3	0.795817	0.730524	0.866946	1.71E-07	2.37E-06
PDCD1	0.809784	0.748206	0.87643	1.71E-07	2.83E-06
CD247	0.76894	0.696672	0.848704	1.81E-07	1.38E-05
CLEC4E	0.730236	0.648864	0.821812	1.83E-07	4.17E-06
PTPRC	0.820221	0.761332	0.883666	1.85E-07	9.97E-07
NKG7	0.848684	0.797904	0.902696	1.87E-07	7.16E-07
KRT17	1.116307	1.071054	1.163472	1.88E-07	0.007115
PYHIN1	0.77825	0.708216	0.85521	1.88E-07	2.44E-06
P2RY13	0.748223	0.670858	0.83451	1.90E-07	6.62E-05
HLA-DRA	0.850648	0.800411	0.904038	1.91E-07	5.31E-07
ARHGAP9	0.756622	0.681107	0.840508	2.01E-07	4.00E-06
PATL2	0.613533	0.510322	0.737617	2.01E-07	1.90E-05
CTSW	0.811491	0.750015	0.878005	2.03E-07	0.000222

HSH2D	0.760139	0.685407	0.843019	2.06E-07	5.38E-06
APOL1	0.825015	0.767232	0.88715	2.08E-07	4.23E-07
CLEC7A	0.73962	0.659989	0.82886	2.11E-07	3.62E-06
PRF1	0.82545	0.767758	0.887478	2.11E-07	1.34E-05
HLA-DQB1	0.828478	0.771631	0.889514	2.12E-07	3.25E-07
IL18RAP	0.571992	0.46312	0.706458	2.15E-07	9.96E-07
HLA-DOA	0.829239	0.772509	0.890135	2.23E-07	5.51E-08
CD7	0.819302	0.75975	0.883522	2.26E-07	4.96E-05
CD74	0.829075	0.772272	0.890057	2.26E-07	1.56E-06
MRPS2	1.60093	1.339616	1.913217	2.27E-07	5.85E-05
SP140	0.734026	0.652914	0.825214	2.27E-07	6.50E-07
TENT5C	0.792396	0.725539	0.865415	2.29E-07	1.28E-05
GIMAP4	0.783896	0.714817	0.859649	2.30E-07	4.74E-06
ARHGAP25	0.750511	0.67316	0.836751	2.32E-07	1.90E-05
SLAMF6	0.804092	0.740302	0.873377	2.34E-07	1.45E-05
PILRA	0.735139	0.654117	0.826195	2.41E-07	1.13E-05
SOD2	0.690216	0.599622	0.794498	2.41E-07	3.10E-07
CXCL13	0.865091	0.818779	0.914022	2.44E-07	7.03E-06
LAX1	0.747894	0.669734	0.835175	2.49E-07	3.60E-06
CD1D	0.68731	0.59601	0.792595	2.52E-07	0.000224
CD8B	0.818212	0.758123	0.883063	2.53E-07	2.31E-06
IRX3	1.299755	1.176294	1.436173	2.63E-07	5.15E-05
HLA-B	0.813293	0.751731	0.879896	2.66E-07	5.29E-06
C16orf54	0.767065	0.693333	0.848638	2.70E-07	1.30E-05
TNFSF13B	0.814456	0.753141	0.880763	2.76E-07	1.11E-05
SIRPG	0.809664	0.746991	0.877594	2.80E-07	1.18E-06
PIM2	0.725413	0.641672	0.820082	2.91E-07	2.90E-05
JAKMIP1	0.676493	0.582636	0.78547	2.92E-07	8.87E-06
PSME2	0.607188	0.501783	0.734735	2.92E-07	5.72E-05
IFNG	0.753938	0.676701	0.83999	3.02E-07	7.05E-06
MLKL	0.679949	0.586501	0.788286	3.16E-07	1.06E-06
CRTAM	0.731956	0.649411	0.824993	3.20E-07	1.71E-05
NR1H3	0.674017	0.579388	0.784102	3.21E-07	9.10E-06
TMSB4X	0.768714	0.69489	0.850381	3.29E-07	6.19E-06
ARID5A	0.62699	0.52411	0.750064	3.31E-07	1.74E-06
FGL2	0.833964	0.777779	0.894207	3.36E-07	1.36E-08
PARP9	0.740714	0.660035	0.831254	3.38E-07	5.66E-06
BTN3A3	0.731209	0.648302	0.824719	3.42E-07	3.74E-05
OTULINL	0.748219	0.669196	0.836573	3.52E-07	5.12E-08
SLA	0.771228	0.697825	0.852352	3.57E-07	3.81E-06
N4BP2L1	0.702454	0.613124	0.804799	3.59E-07	2.46E-09
MS4A6A	0.78517	0.715293	0.861873	3.66E-07	5.65E-06
FASLG	0.759736	0.683327	0.844689	3.76E-07	6.31E-06
CCL5	0.849098	0.797161	0.904419	3.78E-07	1.62E-05
PSTPIP1	0.749724	0.670842	0.837881	3.81E-07	1.76E-05
TFEC	0.699405	0.608934	0.803317	4.22E-07	6.77E-07
TNFRSF9	0.756449	0.678887	0.842872	4.26E-07	1.49E-07
BIRC3	0.803295	0.73789	0.874497	4.31E-07	2.61E-07
PLEK	0.813366	0.750753	0.881201	4.32E-07	6.34E-05
LILRB2	0.773077	0.699597	0.854274	4.40E-07	5.90E-06
PKP1	1.155051	1.092195	1.221525	4.44E-07	0.000417

HLA-DOB	0.7723	0.698576	0.853804	4.47E-07	1.88E-06
GATAD2A	2.025411	1.539437	2.664799	4.61E-07	1.39E-05
UBD	0.856689	0.806553	0.909941	4.97E-07	9.19E-05
OR2I1P	0.856065	0.805736	0.909537	4.98E-07	4.71E-06
GZMH	0.824771	0.765087	0.889111	4.99E-07	6.07E-06
CXCR3	0.819783	0.758638	0.885856	5.05E-07	2.60E-05
IFNAR2	0.619937	0.514436	0.747075	5.07E-07	0.00053
HLA-DPA1	0.835028	0.778288	0.895904	5.12E-07	2.97E-08
CD69	0.759256	0.681813	0.845496	5.23E-07	3.91E-06
SIGLEC10	0.783232	0.711927	0.861678	5.25E-07	2.83E-08
CD3D	0.844399	0.790403	0.902083	5.27E-07	3.64E-06
KLRK1	0.553541	0.439262	0.697551	5.36E-07	5.20E-08
SLC27A4	1.620189	1.3412	1.957211	5.60E-07	4.20E-05
LCP2	0.786373	0.715736	0.86398	5.60E-07	6.92E-07
RABIF	1.804566	1.432069	2.273954	5.60E-07	0.000419
GZMK	0.831875	0.774002	0.894076	5.64E-07	3.19E-05
C1QB	0.841849	0.786904	0.90063	5.76E-07	7.66E-06
HMOX2	2.058895	1.551059	2.733004	5.81E-07	8.13E-05
FCGR3A	0.829407	0.770741	0.89254	5.81E-07	6.60E-05
CCDC88B	0.701966	0.61096	0.806528	5.88E-07	0.000109
MNDA	0.78881	0.718679	0.865785	5.93E-07	1.65E-06
IL15RA	0.725073	0.639112	0.822596	5.94E-07	9.31E-06
AKAP5	0.646266	0.544342	0.767274	6.19E-07	1.82E-06
CIITA	0.784078	0.712562	0.862772	6.20E-07	3.53E-05
CD3G	0.780846	0.708393	0.860708	6.39E-07	2.08E-05
GPR65	0.732239	0.647548	0.828006	6.71E-07	1.28E-06
TBC1D10C	0.772447	0.697612	0.855311	6.83E-07	1.13E-05
TRIM29	1.176758	1.103537	1.254837	6.85E-07	0.014716
HLA-DRB5	0.83743	0.780714	0.898265	7.10E-07	3.33E-05
CLN6	1.561907	1.309229	1.863351	7.33E-07	5.77E-06
IL15	0.639736	0.536009	0.763537	7.46E-07	2.30E-06
C1QC	0.832413	0.77402	0.89521	7.69E-07	3.50E-05
CYBB	0.82775	0.767948	0.892209	7.77E-07	3.41E-05
AIF1	0.800228	0.732449	0.874278	8.00E-07	1.13E-05
GPR84	0.734325	0.649442	0.830303	8.34E-07	6.63E-05
STAC3	0.677141	0.579792	0.790835	8.51E-07	1.86E-06
HCLS1	0.7887	0.71758	0.866869	8.52E-07	5.84E-06
SAMD3	0.514078	0.394348	0.670161	8.72E-07	5.57E-07
KIAA0040	0.779776	0.706104	0.861135	8.99E-07	1.46E-05
HCST	0.79727	0.728331	0.872735	9.11E-07	0.00022
ZNF831	0.641276	0.536986	0.765821	9.28E-07	1.08E-07
RHOV	1.237175	1.136271	1.347039	9.44E-07	0.000307
SLFN12L	0.618022	0.509864	0.749124	9.45E-07	1.13E-06
SNX20	0.771741	0.69575	0.856032	9.63E-07	4.10E-06
IL12RB1	0.782846	0.709784	0.863428	9.70E-07	0.000192
PTPN7	0.796363	0.726963	0.872388	9.85E-07	2.35E-07
SIT1	0.817415	0.754009	0.886154	9.89E-07	8.70E-07
DNAJC5B	0.649755	0.546626	0.772341	1.01E-06	2.74E-06
IL4I1	0.82116	0.758795	0.888651	1.01E-06	1.01E-06
KRT14	1.097989	1.057588	1.139932	1.02E-06	0.028826
IL27	0.555844	0.439163	0.703525	1.03E-06	1.23E-05

CCR2	0.768325	0.691091	0.85419	1.08E-06	1.92E-05
TLR8	0.75093	0.669238	0.842594	1.09E-06	3.30E-07
LST1	0.780004	0.705729	0.862096	1.14E-06	1.42E-07
KLHDC7B	0.75656	0.676071	0.846631	1.17E-06	3.84E-07
TRAT1	0.732977	0.646676	0.830794	1.17E-06	3.14E-06
TTYH3	1.344847	1.193365	1.515559	1.18E-06	0.000214
EPSTI1	0.798924	0.729661	0.874762	1.22E-06	6.20E-06
RASGRP1	0.752035	0.670217	0.843842	1.24E-06	2.16E-05
KIT	1.134527	1.078044	1.193969	1.27E-06	0.002148
CYLD	0.689787	0.593397	0.801834	1.33E-06	1.89E-06
ZMYND15	0.669639	0.569149	0.787872	1.34E-06	4.80E-05
APOBEC3D	0.74397	0.659815	0.83886	1.37E-06	1.47E-06
CD40	0.781071	0.706498	0.863514	1.39E-06	5.50E-05
DTX3L	0.74673	0.663167	0.840822	1.41E-06	0.000207
IL2RG	0.838563	0.780627	0.900799	1.44E-06	7.27E-07
SECTM1	0.800624	0.731402	0.876397	1.44E-06	6.14E-06
CD27	0.837587	0.779343	0.900184	1.44E-06	1.69E-05
DLX3	1.273161	1.153949	1.40469	1.48E-06	0.004107
ADAMDEC1	0.834866	0.775724	0.898516	1.48E-06	9.78E-06
TASL	0.72936	0.641403	0.829378	1.49E-06	1.11E-05
DRAM1	0.721989	0.632236	0.824483	1.51E-06	4.18E-05
CSF1	0.758324	0.67741	0.848903	1.54E-06	3.18E-05
BIN2	0.784752	0.710805	0.866393	1.59E-06	4.97E-05
CARMIL2	0.712781	0.620729	0.818485	1.59E-06	1.69E-05
DENND3	0.656354	0.552636	0.779538	1.60E-06	2.22E-08
CLECL1	0.650275	0.54544	0.77526	1.60E-06	3.06E-07
OLFML2A	1.247704	1.13985	1.365763	1.61E-06	0.004415
LHB	1.267146	1.150157	1.396035	1.66E-06	0.001215
ABCB1	0.685959	0.587921	0.800345	1.67E-06	1.15E-05
TAGAP	0.776493	0.700029	0.861309	1.73E-06	7.72E-07
CALML3	1.142722	1.081917	1.206945	1.73E-06	0.013531
KRT5	1.105204	1.06081	1.151456	1.73E-06	0.040829
KRT6B	1.109942	1.063477	1.158438	1.75E-06	0.01984
CRIP1	0.665775	0.563486	0.786632	1.75E-06	0.000142
ZRSR2	0.55027	0.430648	0.70312	1.78E-06	0.000514
RASAL3	0.788741	0.715551	0.869417	1.79E-06	3.19E-06
TTC24	0.590612	0.475832	0.733081	1.79E-06	9.39E-06
FCGR1A	0.775727	0.698934	0.860956	1.80E-06	2.68E-06
RHOH	0.747443	0.663246	0.84233	1.81E-06	4.27E-07
LILRB4	0.811691	0.74505	0.884292	1.81E-06	8.84E-06
GZMB	0.838936	0.780557	0.90168	1.82E-06	5.20E-06
ANKRD22	0.82076	0.756787	0.890141	1.84E-06	1.49E-05
CD53	0.830117	0.768974	0.896122	1.85E-06	1.07E-05
SP140L	0.758336	0.676722	0.849792	1.92E-06	7.92E-06
ST8SIA4	0.740781	0.654652	0.838242	1.96E-06	1.56E-05
FGD1	1.59486	1.315618	1.933371	2.00E-06	3.57E-07
CYTIP	0.801605	0.731723	0.878161	2.02E-06	0.0001
ACAP1	0.756706	0.674491	0.848942	2.03E-06	1.67E-05
PSMB8	0.753466	0.670394	0.846831	2.04E-06	0.000374
JCHAIN	0.896061	0.856359	0.937604	2.07E-06	7.51E-06
MYO1F	0.75721	0.675053	0.849367	2.07E-06	1.17E-05

ITGAL	0.829807	0.768262	0.896282	2.09E-06	2.99E-05
CLIC2	0.800284	0.729917	0.877434	2.09E-06	0.000458
LCK	0.830444	0.769072	0.896713	2.11E-06	1.59E-05
NCF4	0.777028	0.7	0.862531	2.18E-06	3.15E-05
HAVCR2	0.794695	0.722509	0.874094	2.25E-06	2.99E-06
HPDL	1.243866	1.136292	1.361623	2.26E-06	0.001338
APBB1IP	0.794544	0.722278	0.87404	2.28E-06	3.50E-06
THEMIS	0.708598	0.614268	0.817414	2.29E-06	4.02E-08
CORO1A	0.827024	0.764382	0.894799	2.29E-06	2.94E-05
MZB1	0.87347	0.825785	0.92391	2.32E-06	8.11E-05
HK3	0.776073	0.698576	0.862168	2.32E-06	1.94E-05
GAB3	0.685243	0.585754	0.80163	2.33E-06	0.000121
NECTIN1	1.332118	1.182504	1.500661	2.38E-06	0.002259
CNFN	1.170643	1.096456	1.249849	2.40E-06	0.00137
HLA-C	0.793311	0.720539	0.873434	2.40E-06	4.47E-05
GNGT2	0.711014	0.617027	0.819318	2.42E-06	8.38E-05
GIMAP5	0.611462	0.49831	0.750307	2.46E-06	4.51E-07
GYS1	1.596434	1.313949	1.939651	2.50E-06	0.012258
CD3E	0.850024	0.794407	0.909535	2.52E-06	1.71E-05
EBI3	0.799926	0.728909	0.877863	2.52E-06	3.66E-05
FOXM1	1.415042	1.224451	1.635299	2.56E-06	9.25E-07
CCR1	0.793079	0.720027	0.873542	2.58E-06	1.50E-05
CYTH4	0.765911	0.685264	0.856048	2.63E-06	2.02E-05
SPATC1	0.369365	0.243757	0.5597	2.64E-06	3.07E-06
MICALL1	1.59825	1.314136	1.943789	2.66E-06	2.51E-06
SCIMP	0.769683	0.689984	0.858588	2.68E-06	4.34E-05
MAP3K7CL	0.665934	0.561923	0.789196	2.70E-06	2.37E-05
CD96	0.786089	0.710835	0.869309	2.76E-06	2.17E-05
OCA2	1.112423	1.063936	1.163119	2.79E-06	2.02E-05
UBASH3A	0.763705	0.682247	0.854888	2.81E-06	2.57E-05
ANKRD9	1.343055	1.187115	1.519478	2.82E-06	1.47E-05
TTC39C	0.737173	0.648829	0.837546	2.84E-06	0.002255
NLRC3	0.709617	0.614692	0.819202	2.84E-06	1.10E-05
PTGER2	0.674784	0.572301	0.795619	2.86E-06	2.13E-05
FCRL3	0.755764	0.672129	0.849806	2.87E-06	4.42E-07
NLRP6	0.518999	0.394313	0.683112	2.89E-06	6.24E-07
GCA	0.690923	0.591771	0.806688	2.90E-06	0.000848
AOAH	0.808678	0.739807	0.883961	2.93E-06	2.93E-05
CFB	0.665098	0.560459	0.789273	3.02E-06	0.000145
TRAF3IP3	0.755481	0.671584	0.849858	3.03E-06	2.15E-06
KRT6A	1.098994	1.056251	1.143467	3.10E-06	0.048411
GPR141	0.609573	0.495019	0.750637	3.15E-06	6.24E-06
IL10RA	0.807854	0.738471	0.883756	3.21E-06	5.48E-05
MCOLN2	0.743372	0.65613	0.842213	3.22E-06	7.69E-05
NEURL3	0.628274	0.516597	0.764094	3.25E-06	1.10E-05
PDCD1LG2	0.775017	0.696145	0.862826	3.25E-06	7.10E-07
NECTIN4	1.256859	1.141398	1.383999	3.32E-06	0.002456
LY75	0.695791	0.597149	0.810726	3.32E-06	3.06E-06
FCGR1B	0.632891	0.521738	0.767725	3.44E-06	1.62E-05
CD48	0.828418	0.764974	0.897124	3.65E-06	3.29E-05
TMEM156	0.708862	0.612719	0.820092	3.71E-06	1.01E-05

XRN1	0.717231	0.622968	0.825758	3.78E-06	0.001299
P2RY10	0.783074	0.705956	0.868616	3.79E-06	9.86E-06
ARMH1	0.65496	0.547334	0.78375	3.83E-06	6.62E-05
B3GNT4	1.771318	1.389846	2.257492	3.83E-06	0.003284
VNN2	0.757186	0.672841	0.852104	3.91E-06	5.47E-07
CDIP1	1.527238	1.27586	1.828144	3.93E-06	0.000271
TRANK1	0.751371	0.665421	0.848422	3.99E-06	9.57E-07
NLRC4	0.640207	0.529644	0.773851	4.02E-06	2.50E-06
P2RY6	0.72272	0.629389	0.829891	4.17E-06	0.000536
DPYD	0.797526	0.724268	0.878193	4.18E-06	1.98E-06
CALML5	1.1126	1.063111	1.164393	4.30E-06	0.033254
A2ML1	1.209375	1.115183	1.311524	4.33E-06	0.000694
FGD2	0.73671	0.646687	0.839266	4.33E-06	1.12E-05
MARCHF1	0.714218	0.618671	0.824522	4.36E-06	3.15E-05
REC8	0.693543	0.593227	0.810823	4.42E-06	0.0001
SPATA13	0.741772	0.652932	0.8427	4.44E-06	0.004763
ZNF80	0.385003	0.256056	0.578886	4.50E-06	4.60E-07
GIMAP1	0.741807	0.652913	0.842802	4.52E-06	1.63E-05
PITX1	1.278091	1.150836	1.419417	4.53E-06	0.012524
JAML	0.761815	0.678143	0.855811	4.58E-06	7.06E-06
IGSF6	0.792534	0.717451	0.875475	4.68E-06	1.04E-05
NUB1	0.595357	0.476822	0.743359	4.69E-06	0.000803
LAT2	0.745264	0.657052	0.845319	4.78E-06	4.71E-06
CD300LF	0.765773	0.683032	0.858537	4.78E-06	8.46E-06
BATF2	0.787186	0.710472	0.872184	4.78E-06	0.000156
CTSS	0.817057	0.749286	0.890959	4.80E-06	5.43E-06
KRT16	1.100837	1.056399	1.147145	4.88E-06	0.020052
COL17A1	1.167875	1.092579	1.24836	5.02E-06	5.73E-05
FLAD1	1.801157	1.398351	2.319993	5.21E-06	0.000161
KRTDAP	1.113014	1.062885	1.165506	5.27E-06	0.00054
ETV7	0.811562	0.741764	0.887929	5.35E-06	3.65E-05
SEMA6A	1.219121	1.119383	1.327746	5.37E-06	0.002986
IFIT2	0.795955	0.721367	0.878255	5.47E-06	4.77E-06
VAV1	0.800753	0.72757	0.881297	5.52E-06	6.80E-06
ESRP2	1.297777	1.159755	1.452224	5.54E-06	0.000547
FLT3LG	0.631923	0.518362	0.770361	5.59E-06	0.00013
ZNF25	0.717927	0.622229	0.828343	5.62E-06	0.0006
ALOX5	0.79042	0.714096	0.874901	5.64E-06	2.02E-05
BST2	0.846361	0.787544	0.909572	5.65E-06	7.00E-07
NT5DC2	1.403071	1.212138	1.624079	5.69E-06	1.58E-06
TIMD4	0.732975	0.640927	0.838243	5.71E-06	9.25E-08
PTK2B	0.696984	0.596333	0.814623	5.72E-06	0.000114
PTPN6	0.810037	0.739559	0.88723	5.72E-06	3.42E-07
ZC3H12D	0.627138	0.512612	0.76725	5.76E-06	1.10E-05
RUFY4	0.467297	0.336346	0.649229	5.77E-06	0.000471
CS	1.743909	1.371184	2.217951	5.81E-06	3.22E-05
SEPTIN1	0.762613	0.678284	0.857426	5.82E-06	0.000251
ARHGEF3	0.724282	0.62996	0.832725	5.86E-06	0.000583
KCNA3	0.724272	0.629898	0.832787	5.93E-06	2.97E-05
TESPA1	0.744718	0.655519	0.846055	5.95E-06	1.06E-07
CD84	0.784014	0.705605	0.871135	6.01E-06	1.29E-05

TNFRSF18	0.775677	0.694848	0.865908	6.06E-06	1.99E-05
TBC1D16	1.35274	1.18672	1.541987	6.11E-06	0.001173
ABCD2	0.582602	0.460985	0.736304	6.11E-06	5.48E-05
SLC25A3	1.84106	1.413047	2.398718	6.15E-06	3.72E-05
MEI1	0.7139	0.616789	0.826301	6.26E-06	0.001271
PROM2	1.234688	1.126753	1.352963	6.28E-06	0.000133
CD200R1	0.67962	0.574643	0.803774	6.43E-06	2.38E-06
WAS	0.805982	0.733837	0.88522	6.54E-06	3.01E-06
ITGB2	0.828714	0.763649	0.899324	6.69E-06	2.05E-05
DSC3	1.171072	1.093265	1.254418	6.73E-06	0.03224
ZBED2	0.781118	0.701404	0.869891	6.86E-06	0.000618
ADA2	0.826827	0.761059	0.898279	6.90E-06	4.84E-05
RBM43	0.722943	0.627554	0.832832	7.00E-06	0.001169
S1PR4	0.79918	0.724739	0.881267	7.00E-06	0.000987
KLRC1	0.565738	0.441277	0.725302	7.01E-06	8.56E-08
RNF213	0.673916	0.56733	0.800527	7.03E-06	0.020515
FCER1G	0.835119	0.771988	0.903412	7.03E-06	4.60E-05
HSD11B1	0.791834	0.715172	0.876713	7.04E-06	4.92E-06
DTHD1	0.536704	0.409026	0.704238	7.13E-06	1.94E-06
RSAD2	0.800988	0.727003	0.882501	7.20E-06	8.16E-06
ADAM28	0.710286	0.611698	0.824764	7.23E-06	1.96E-06
GIMAP6	0.782347	0.702783	0.87092	7.27E-06	0.000137
NCKAP1L	0.802462	0.728886	0.883466	7.28E-06	2.35E-05
IFITM3	0.749593	0.660878	0.850216	7.30E-06	0.001891
SPOCK2	0.813146	0.742859	0.890084	7.31E-06	4.05E-06
HLA-F	0.811754	0.741004	0.88926	7.38E-06	4.20E-05
ICOS	0.771277	0.688442	0.864078	7.46E-06	0.000341
FYB1	0.821185	0.753335	0.895147	7.56E-06	6.96E-07
CST7	0.850226	0.791905	0.912843	7.64E-06	0.000253
NSMF	1.437292	1.225886	1.685155	7.86E-06	0.002501
KLK13	1.203261	1.109398	1.305064	7.99E-06	0.008595
TNFAIP8L2	0.790077	0.712435	0.876181	8.03E-06	0.000531
CD47	0.665504	0.556546	0.795793	8.05E-06	0.033196
IKZF1	0.798213	0.722978	0.881278	8.12E-06	1.56E-06
PCED1B	0.805087	0.731935	0.88555	8.16E-06	8.36E-06
PARVG	0.759924	0.673562	0.857359	8.18E-06	5.81E-06
C1orf162	0.770499	0.687082	0.864042	8.21E-06	3.40E-05
RTP5	0.482861	0.350586	0.665042	8.30E-06	3.07E-06
AIM2	0.859127	0.803631	0.918455	8.33E-06	0.000142
TMEM273	0.732223	0.638413	0.839818	8.37E-06	0.000243
IFIT5	0.744823	0.654267	0.847912	8.41E-06	0.008047
C3AR1	0.804589	0.731163	0.885389	8.46E-06	6.22E-06
NCS1	1.360353	1.18796	1.557762	8.54E-06	4.09E-05
RASSF4	0.769201	0.685263	0.86342	8.55E-06	7.70E-05
NFKBIA	0.66722	0.558293	0.797398	8.60E-06	0.001374
APOL4	0.786051	0.706923	0.874036	8.71E-06	0.000241
FUT2	1.48493	1.247351	1.767761	8.80E-06	0.039142
TNFRSF17	0.818894	0.749776	0.894384	8.96E-06	1.04E-06
SIRPB2	0.699576	0.597511	0.819075	8.98E-06	0.000188
ABCG4	1.8826	1.4238	2.489243	9.03E-06	0.001623
SATB1	0.797315	0.721401	0.881218	9.12E-06	0.000113

FGF1	0.804086	0.730193	0.885456	9.27E-06	0.000314
GSDMD	0.731515	0.637056	0.839978	9.34E-06	0.012036
FUT8	0.715368	0.616838	0.829638	9.42E-06	8.03E-05
DUSP6	0.76608	0.680886	0.861933	9.42E-06	0.001659
DOK2	0.804049	0.730083	0.885509	9.44E-06	9.53E-05
GRWD1	1.748595	1.365411	2.239314	9.52E-06	4.70E-05
APOBEC3F	0.756007	0.667952	0.85567	9.56E-06	1.54E-06
PIK3R6	0.702477	0.600775	0.821396	9.61E-06	1.72E-05
JSRP1	0.74167	0.649717	0.846638	9.64E-06	1.56E-05
LAMP3	0.781416	0.700526	0.871646	9.69E-06	0.000364
PIK3R2	2.030491	1.483557	2.77906	9.72E-06	0.001361
SLFN12	0.751483	0.662124	0.852903	9.72E-06	0.001167
LIF	0.862912	0.808321	0.92119	9.79E-06	0.000147
RAP1A	0.676799	0.569256	0.80466	9.80E-06	0.006593
HLA-DQB2	0.865251	0.811478	0.922587	9.81E-06	0.000406
TRIM21	0.702068	0.600158	0.821281	9.85E-06	2.12E-05
RAB25	1.16943	1.090987	1.253514	9.96E-06	0.00764
TTYH2	1.255828	1.135039	1.389471	1.01E-05	0.000588
NUGGC	0.664933	0.554713	0.797054	1.02E-05	6.22E-05
LAIR1	0.796191	0.719541	0.881006	1.02E-05	1.45E-05
CARD11	0.799501	0.723854	0.883053	1.02E-05	1.31E-05
POLDIP2	1.943869	1.446874	2.611579	1.02E-05	0.000731
LYZ	0.871043	0.81921	0.926156	1.03E-05	3.93E-06
ZNF683	0.742686	0.650703	0.847673	1.04E-05	8.10E-05
PAK4	1.571204	1.285223	1.92082	1.04E-05	0.006094
GBP3	0.822322	0.753754	0.897128	1.06E-05	0.000999
IL2RB	0.832966	0.767885	0.903562	1.07E-05	0.000189
RGS18	0.697557	0.594194	0.818902	1.07E-05	5.75E-05
CLEC6A	0.431951	0.2972	0.6278	1.08E-05	1.48E-07
PHF11	0.730335	0.634933	0.840071	1.08E-05	0.000205
DOCK2	0.78639	0.706535	0.875271	1.09E-05	1.56E-06
CSGALNACT	0.815374	0.744476	0.893024	1.09E-05	0.035833
CD6	0.807723	0.734398	0.88837	1.09E-05	0.00012
SUSD3	0.794261	0.716714	0.880198	1.11E-05	1.45E-05
NCKAP5L	1.619646	1.306175	2.008348	1.11E-05	0.000928
SERPINB5	1.171675	1.091703	1.257504	1.12E-05	0.002838
ICAM3	0.757595	0.669321	0.85751	1.12E-05	0.000209
HES2	1.180035	1.095968	1.270551	1.13E-05	0.011004
SASH3	0.829495	0.763018	0.901764	1.15E-05	2.53E-05
SERPINB13	1.25932	1.13601	1.396013	1.16E-05	0.013915
C1RL	0.770527	0.685735	0.865804	1.17E-05	0.00123
SPI1	0.811741	0.739373	0.891193	1.20E-05	1.09E-05
LCP1	0.836444	0.772133	0.906111	1.21E-05	2.38E-05
PRKACB	0.771046	0.68622	0.866357	1.23E-05	0.006728
DDX60L	0.745099	0.653002	0.850184	1.24E-05	0.000161
CEACAM21	0.764313	0.67743	0.862339	1.27E-05	7.66E-06
TNFRSF1B	0.796515	0.719166	0.882183	1.27E-05	0.000222
FCRL5	0.738512	0.644538	0.846187	1.27E-05	2.06E-06
CTNS	1.609181	1.299629	1.992463	1.28E-05	4.65E-05
TAP1	0.804291	0.729331	0.886955	1.28E-05	0.000623
GMFG	0.796934	0.719591	0.88259	1.31E-05	3.59E-05

MYBPC3	0.109108	0.040275	0.29558	1.32E-05	0.000501
CCL25	0.508006	0.374567	0.688982	1.32E-05	9.86E-05
EMB	0.813137	0.740867	0.892457	1.33E-05	3.92E-06
SLC7A7	0.774148	0.689828	0.868774	1.36E-05	7.50E-06
SLC5A3	0.810022	0.736623	0.890734	1.38E-05	0.00052
IRAG2	0.746171	0.653882	0.851485	1.38E-05	1.02E-05
ARRDC5	0.525995	0.393619	0.70289	1.40E-05	5.34E-05
SPRR1B	1.100917	1.054157	1.149752	1.41E-05	0.00041
AQP5	1.197461	1.103821	1.299045	1.44E-05	0.006338
PTPRE	0.762013	0.673932	0.861606	1.45E-05	5.76E-05
PRADC1	1.554902	1.273544	1.898419	1.46E-05	8.53E-05
KRT78	1.236626	1.123361	1.361312	1.47E-05	0.014275
RNASE6	0.800142	0.723347	0.885091	1.48E-05	0.000232
VAMP5	0.812234	0.739268	0.892402	1.49E-05	4.62E-05
LAPTM5	0.836707	0.771802	0.907069	1.51E-05	1.30E-06
SPRR1A	1.106331	1.056837	1.158144	1.51E-05	0.010119
TMEM150B	0.759061	0.669959	0.860012	1.51E-05	5.08E-05
IVL	1.13557	1.07197	1.202943	1.54E-05	0.000843
TMEM97	1.411054	1.206939	1.649687	1.57E-05	6.19E-05
CSF2RB	0.808384	0.733976	0.890335	1.58E-05	4.95E-05
SLAMF8	0.833122	0.766837	0.905137	1.59E-05	3.89E-06
USP35	1.415682	1.208806	1.657963	1.61E-05	0.01436
ARL11	0.684108	0.575591	0.813084	1.65E-05	7.28E-06
TNFRSF4	0.788201	0.707308	0.878345	1.65E-05	0.000401
GPR174	0.783826	0.701589	0.875704	1.66E-05	2.91E-07
PLA2G4D	1.297774	1.152617	1.461211	1.66E-05	0.00428
EVPL	1.186007	1.097404	1.281764	1.66E-05	0.000979
CCL4L2	0.823323	0.753597	0.899502	1.66E-05	0.000183
GPI	1.490092	1.242686	1.786752	1.67E-05	0.004972
KLK8	1.199034	1.103939	1.30232	1.67E-05	0.011211
CD4	0.817568	0.745923	0.896094	1.67E-05	8.48E-05
C19orf38	0.739027	0.643915	0.848189	1.69E-05	0.001373
GNLY	0.820297	0.749484	0.897801	1.71E-05	7.40E-05
LGALS9	0.795623	0.716891	0.883003	1.71E-05	3.64E-05
GRIN3A	0.492502	0.356593	0.680211	1.72E-05	7.37E-05
SLFN13	0.71649	0.615447	0.834123	1.72E-05	0.000166
GJB5	1.219148	1.113785	1.334479	1.73E-05	0.000227
NFAM1	0.778552	0.694516	0.872757	1.74E-05	0.000148
CD300C	0.759918	0.670371	0.861427	1.77E-05	1.95E-06
CCNI2	0.351497	0.21804	0.566639	1.78E-05	0.01146
CD5	0.828717	0.760565	0.902976	1.78E-05	2.31E-05
VPS53	1.613148	1.296603	2.006974	1.78E-05	0.004682
SEL1L3	0.837612	0.772397	0.908332	1.83E-05	0.000851
RWDD2A	0.630056	0.510001	0.778373	1.84E-05	0.003394
DAPP1	0.762724	0.673729	0.863474	1.88E-05	5.80E-06
SERPING1	0.813616	0.740233	0.894274	1.89E-05	0.000574
SIGLEC1	0.806529	0.730778	0.890131	1.93E-05	0.000122
RTN1	0.772457	0.686174	0.869589	1.94E-05	1.12E-05
PARP15	0.729784	0.631564	0.843278	1.94E-05	1.02E-07
IFI44L	0.835979	0.770003	0.907608	1.94E-05	5.34E-07
TNFAIP8	0.740991	0.645743	0.850288	1.95E-05	0.000361

JAK2	0.744337	0.649993	0.852375	1.96E-05	0.000126
CLEC2D	0.745315	0.651206	0.853025	1.97E-05	0.00036
DSG3	1.172635	1.089918	1.26163	1.98E-05	0.00642
HIVEP3	0.730197	0.631956	0.84371	2.00E-05	0.000118
TGM3	1.213046	1.110024	1.32563	2.00E-05	0.046238
PPP2R1A	1.929255	1.42638	2.609421	2.00E-05	0.001101
EVI2A	0.821344	0.750316	0.899096	2.00E-05	0.000632
BCL2L14	0.415863	0.277772	0.622603	2.03E-05	3.78E-06
MOB3C	0.614538	0.491215	0.768822	2.04E-05	0.000191
CTBS	0.707074	0.602799	0.829388	2.06E-05	0.002937
IPCEF1	0.673552	0.561521	0.807935	2.06E-05	8.54E-06
TANK	0.720325	0.619267	0.837874	2.11E-05	0.015979
SOCS1	0.791148	0.71019	0.881334	2.11E-05	0.000764
LRRK2	0.773685	0.687402	0.870798	2.11E-05	0.001406
IRF8	0.824354	0.754129	0.901118	2.12E-05	1.77E-05
ASCL2	0.731514	0.633337	0.84491	2.12E-05	0.002001
FAM83C	1.229793	1.11774	1.35308	2.20E-05	0.000189
XCL1	0.750006	0.656674	0.856602	2.21E-05	4.37E-06
CTLA4	0.825906	0.756056	0.90221	2.21E-05	2.47E-05
RNF223	1.622575	1.297131	2.029673	2.26E-05	0.018595
LTA	0.750032	0.656353	0.857081	2.38E-05	4.23E-05
CBLC	1.238073	1.12131	1.366994	2.39E-05	0.037465
SELPLG	0.821386	0.749735	0.899883	2.39E-05	5.09E-05
ARHGAP30	0.814437	0.740464	0.895799	2.39E-05	1.93E-06
CLEC10A	0.822582	0.751281	0.900649	2.42E-05	0.000177
BOK	1.208428	1.106748	1.319449	2.42E-05	0.000519
LRRC43	1.404043	1.199361	1.643656	2.43E-05	0.000182
HSD11B2	1.330813	1.165367	1.519748	2.45E-05	0.000477
TUBA1C	1.455975	1.222792	1.733626	2.46E-05	0.002055
CSF1R	0.825909	0.755622	0.902735	2.50E-05	5.62E-06
TEAD4	1.443467	1.216886	1.712236	2.52E-05	0.013333
C4orf50	0.031458	0.006279	0.157608	2.59E-05	5.68E-06
IL18	0.818432	0.745485	0.898517	2.59E-05	1.71E-06
LILRB3	0.692586	0.58363	0.821883	2.60E-05	5.69E-05
IQANK1	1.326521	1.162868	1.513206	2.60E-05	0.000132
FOXQ1	1.313528	1.156758	1.491546	2.60E-05	0.02635
SFN	1.098436	1.051384	1.147593	2.63E-05	0.003149
WIPF1	0.818187	0.745065	0.898486	2.66E-05	0.001718
SMARCA4	1.669125	1.314177	2.119941	2.67E-05	0.026729
MBD4	0.656489	0.539333	0.799095	2.72E-05	0.017188
ZBTB32	0.658374	0.541551	0.800399	2.74E-05	0.000241
INPP5D	0.799979	0.720721	0.887953	2.76E-05	1.20E-05
IFIT3	0.820608	0.748144	0.900091	2.77E-05	4.57E-05
PSD4	0.77224	0.684326	0.871447	2.77E-05	0.000719
GGT6	1.249193	1.125746	1.386178	2.78E-05	0.00028
SMTNL1	0.551808	0.417831	0.728744	2.79E-05	9.59E-05
CPEB3	0.571902	0.440296	0.742844	2.82E-05	0.001212
GPX2	1.258705	1.130143	1.401893	2.84E-05	0.014398
IL32	0.840887	0.775332	0.911985	2.86E-05	3.27E-05
ITK	0.786945	0.703389	0.880427	2.87E-05	0.000373
MS4A4A	0.802529	0.723915	0.889681	2.89E-05	0.000255

NCOA7	0.721329	0.618904	0.840703	2.91E-05	0.003472
DOK1	0.709656	0.604112	0.833638	2.98E-05	0.023867
MFSD5	1.739192	1.341069	2.255505	3.01E-05	0.000199
SP100	0.73165	0.631728	0.847377	3.04E-05	0.000228
CD33	0.740981	0.643463	0.853277	3.13E-05	5.62E-05
BTLA	0.728427	0.627444	0.845662	3.16E-05	5.42E-05
APOBEC3H	0.762172	0.670616	0.866229	3.19E-05	1.12E-05
ZC3H6	0.650675	0.531251	0.796945	3.27E-05	0.009123
GALM	0.797842	0.717191	0.887562	3.27E-05	0.00488
TLDC2	0.60092	0.472535	0.764187	3.28E-05	8.31E-05
TAX1BP3	1.516705	1.24602	1.846193	3.28E-05	0.004482
ACSL5	0.811244	0.734951	0.895457	3.31E-05	0.000337
ABCA12	1.295381	1.146381	1.463746	3.31E-05	2.88E-05
ENPP4	0.802265	0.722991	0.890232	3.32E-05	8.31E-05
GPR18	0.75822	0.665287	0.864134	3.34E-05	0.000819
AMPD1	0.559867	0.42567	0.736369	3.34E-05	2.34E-05
TNIP3	0.617303	0.491489	0.775322	3.35E-05	1.72E-06
PLA2G4E	1.261841	1.130487	1.408456	3.37E-05	0.029159
LY6D	1.132835	1.067948	1.201665	3.41E-05	0.044636
GIMAP8	0.781713	0.695765	0.878278	3.41E-05	9.23E-05
TLR1	0.775703	0.687896	0.874718	3.42E-05	0.000195
FBRSL1	1.616123	1.287567	2.02852	3.48E-05	0.000874
SPN	0.817545	0.743168	0.899366	3.48E-05	9.78E-06
IFI30	0.671684	0.556298	0.811003	3.50E-05	0.004796
HLA-E	0.771195	0.681886	0.872202	3.51E-05	9.21E-05
XAF1	0.792996	0.71045	0.885133	3.54E-05	0.000144
CD226	0.63557	0.512676	0.787922	3.56E-05	3.20E-05
MBNL1	0.745195	0.64819	0.856716	3.57E-05	0.007042
CYSLTR1	0.642521	0.520916	0.792514	3.59E-05	2.90E-05
TXNDC11	0.552648	0.417092	0.732262	3.62E-05	0.001861
LY9	0.721963	0.618504	0.842729	3.66E-05	0.001033
CMPK2	0.789061	0.705116	0.883	3.66E-05	5.12E-05
KCNN4	0.876652	0.823515	0.933218	3.68E-05	0.002611
CLEC4D	0.483375	0.342234	0.682725	3.69E-05	3.44E-05
RASSF5	0.802906	0.723399	0.891152	3.69E-05	0.000266
KLK5	1.141588	1.071964	1.215735	3.72E-05	0.004338
POU2AF1	0.835364	0.766878	0.909966	3.76E-05	5.67E-06
IRF9	0.705037	0.597039	0.832571	3.79E-05	0.000392
PIK3AP1	0.81019	0.732897	0.895634	3.88E-05	5.80E-05
CBX7	0.756011	0.661695	0.863771	3.89E-05	0.000471
BLOC1S3	1.837615	1.374661	2.456479	3.98E-05	0.000238
RCSD1	0.808007	0.729874	0.894504	3.98E-05	7.65E-06
SQOR	0.796701	0.71482	0.887961	4.00E-05	0.000353
ABCC2	1.151483	1.076516	1.23167	4.02E-05	0.000487
C11orf21	0.692374	0.580924	0.825205	4.03E-05	0.001067
OASL	0.821797	0.74824	0.902585	4.09E-05	0.00014
SLFN11	0.814919	0.738967	0.898678	4.13E-05	6.17E-05
ZNF703	1.22523	1.111802	1.350229	4.16E-05	0.002066
THYN1	0.608848	0.480182	0.77199	4.20E-05	0.006803
SELL	0.849901	0.786237	0.918721	4.24E-05	0.00052
ABI3	0.798934	0.717506	0.889604	4.26E-05	0.000243

CTSV	1.255196	1.125709	1.399577	4.28E-05	0.000119
RTL10	1.57855	1.268455	1.964452	4.29E-05	0.005606
LTB	0.857217	0.796196	0.922916	4.33E-05	2.91E-05
CD79A	0.888427	0.839422	0.940292	4.38E-05	6.71E-06
RAB32	1.330193	1.160056	1.525283	4.38E-05	0.009791
DOCK8	0.799295	0.717843	0.889989	4.40E-05	0.000127
ACY3	0.751776	0.655551	0.862126	4.45E-05	0.000324
TNFSF10	0.833736	0.764018	0.909817	4.48E-05	1.10E-07
ACSL4	0.777601	0.689089	0.877482	4.51E-05	0.017911
PRDM1	0.786677	0.701021	0.882799	4.52E-05	0.001204
GPR87	1.251121	1.12343	1.393325	4.52E-05	0.010962
RPP25	1.246433	1.12123	1.385617	4.53E-05	0.004893
TYROBP	0.835326	0.766107	0.910801	4.56E-05	4.70E-05
GSTP1	1.236839	1.116684	1.369923	4.57E-05	8.57E-05
SIGLEC11	0.699403	0.588915	0.83062	4.59E-05	0.000401
GLIPR1	0.784422	0.697935	0.881625	4.63E-05	4.78E-05
OVOL2	1.683346	1.310225	2.162724	4.63E-05	0.00138
FAM83A	1.236017	1.116136	1.368774	4.69E-05	0.000411
PCMTD1	0.736789	0.635999	0.853552	4.71E-05	0.013413
ERLEC1	0.7207	0.615437	0.843968	4.79E-05	0.046
BCL6	0.691359	0.578643	0.826031	4.81E-05	0.008391
CIB2	1.301962	1.146406	1.478625	4.81E-05	0.000422
ERAP2	0.864465	0.80583	0.927367	4.82E-05	0.002434
P2RY14	0.691011	0.578091	0.825988	4.91E-05	0.000102
FLG	1.183689	1.091085	1.284152	4.96E-05	0.007408
SERPINB3	1.164213	1.081757	1.252955	4.98E-05	0.036176
LRRC8C	0.727956	0.624425	0.848653	4.98E-05	8.68E-05
MARCO	0.872995	0.817544	0.932208	4.98E-05	0.004341
CEACAM4	0.609551	0.479871	0.774275	4.99E-05	0.001938
SLAMF7	0.870966	0.81468	0.931141	5.06E-05	0.000108
KRCC1	0.771484	0.680506	0.874625	5.07E-05	0.001232
TPCN2	1.23234	1.113874	1.363406	5.09E-05	0.004933
GGTA1	0.777338	0.688117	0.878128	5.14E-05	0.0027
MYO7A	0.763554	0.670054	0.870101	5.17E-05	4.19E-05
FAM20A	0.802288	0.721109	0.892606	5.18E-05	0.002166
FCN1	0.804248	0.723656	0.893815	5.26E-05	0.001569
ZNF730	1.561869	1.258068	1.939032	5.34E-05	0.016658
TIFAB	0.664011	0.544377	0.809936	5.35E-05	0.000375
BNIPL	1.241293	1.11765	1.378615	5.40E-05	0.013519
COX6A1	1.548487	1.252239	1.914819	5.43E-05	0.000416
RASA2	0.728868	0.6251	0.849863	5.44E-05	0.026512
HEATR9	0.219785	0.10521	0.459136	5.56E-05	0.003114
SLC2A5	0.793239	0.708707	0.887853	5.60E-05	0.002622
PLA2G5	0.783359	0.695619	0.882165	5.61E-05	0.002467
PAEP	1.079885	1.040251	1.12103	5.62E-05	0.024455
PARP11	0.705362	0.595165	0.835963	5.64E-05	0.008046
STIM2	0.748154	0.649557	0.861716	5.72E-05	0.003539
TOMM40	1.601446	1.272952	2.01471	5.81E-05	4.17E-05
NOD2	0.74762	0.648575	0.86179	6.04E-05	2.41E-05
MANEA	0.761196	0.666129	0.86983	6.10E-05	0.011016
GABRA5	1.240737	1.116548	1.37874	6.11E-05	0.001142

NOTCH3	1.254218	1.122694	1.401151	6.14E-05	0.010965
DEF6	0.802027	0.719996	0.893404	6.14E-05	0.021282
ZAP70	0.831016	0.759081	0.909768	6.15E-05	0.001747
HCK	0.824107	0.749644	0.905966	6.23E-05	4.44E-06
NCR3	0.757258	0.660888	0.867679	6.24E-05	7.53E-05
TCAF2	0.736951	0.634649	0.855745	6.26E-05	4.94E-07
KRT80	1.164554	1.080851	1.254738	6.26E-05	0.001033
CDH3	1.123109	1.06104	1.18881	6.27E-05	0.00397
TMEM176B	0.83604	0.765846	0.912667	6.27E-05	5.76E-06
SLC35A4	1.722192	1.319605	2.247601	6.30E-05	0.001042
APOL2	0.731801	0.627987	0.852776	6.33E-05	3.26E-05
CASP8	0.718206	0.610661	0.844692	6.35E-05	0.005128
LRRC25	0.79763	0.713936	0.891134	6.39E-05	0.000364
ITM2B	0.714431	0.605752	0.842608	6.50E-05	0.006504
EIF4E3	0.763801	0.669098	0.871908	6.62E-05	0.000313
LY6G6C	1.154231	1.075688	1.238509	6.63E-05	0.003156
TAPBP	0.734751	0.631447	0.854955	6.69E-05	1.91E-05
ATP8B4	0.590603	0.455888	0.765127	6.70E-05	0.000164
STK17A	0.764324	0.669692	0.872327	6.74E-05	0.001078
C19orf47	1.816666	1.354363	2.436772	6.77E-05	0.000378
ATP5F1B	1.473275	1.21756	1.782696	6.78E-05	0.001362
EPS8L1	1.310259	1.1471	1.496625	6.82E-05	0.000371
TSPAN9	1.397493	1.185243	1.647752	6.83E-05	0.000227
ARHGDIB	0.813408	0.734778	0.900451	6.85E-05	0.000311
PRKCQ	0.783192	0.694418	0.883315	6.85E-05	0.000443
STAP1	0.769869	0.67683	0.875697	6.90E-05	6.74E-06
CASS4	0.683669	0.566889	0.824504	6.91E-05	1.88E-05
TUBB4B	1.447756	1.20651	1.737241	6.93E-05	0.000164
TSPAN32	0.721817	0.61471	0.847586	6.96E-05	0.000397
CCDC28A	0.650065	0.525767	0.803748	6.96E-05	0.000489
SPRR2A	1.117503	1.057933	1.180427	7.04E-05	0.023495
SLAMF1	0.835632	0.764824	0.912995	7.04E-05	5.83E-06
SPRR2E	1.109497	1.054079	1.167829	7.05E-05	0.003428
AK1	1.525838	1.238815	1.879363	7.06E-05	0.000776
AFTPH	0.670547	0.550485	0.816794	7.18E-05	0.004862
ALOX5AP	0.836761	0.766284	0.913721	7.19E-05	9.95E-05
ACRBP	0.683512	0.566388	0.824855	7.26E-05	0.000272
GRIPAP1	0.542015	0.400512	0.733512	7.26E-05	0.003804
TRAPPC3L	0.122004	0.043141	0.345032	7.30E-05	0.000182
SUCNR1	0.730172	0.625034	0.852995	7.36E-05	0.00242
IRAK2	0.764704	0.669661	0.873237	7.44E-05	2.03E-05
IFI35	0.79537	0.710185	0.890773	7.46E-05	0.00131
FAM83H	1.288385	1.136562	1.460489	7.46E-05	0.000233
DENND1B	0.712782	0.602812	0.842813	7.49E-05	0.002826
DUSP5	0.815758	0.737549	0.90226	7.49E-05	0.002785
TRIM32	1.515474	1.233617	1.861729	7.51E-05	0.027614
CASP5	0.624664	0.494855	0.788524	7.53E-05	5.72E-07
SKAP2	0.792465	0.706224	0.889237	7.59E-05	0.024866
RNASET2	0.738587	0.635638	0.858211	7.61E-05	0.000662
JPT2	1.698135	1.306245	2.207597	7.63E-05	4.68E-05
ZNF267	0.716726	0.607647	0.845385	7.69E-05	0.00102

SSTR2	0.544997	0.403354	0.736381	7.73E-05	0.000258
CFLAR	0.666351	0.544846	0.814951	7.74E-05	0.005018
KLRB1	0.780637	0.690399	0.882669	7.77E-05	2.45E-05
OAS1	0.827925	0.753892	0.909229	7.78E-05	1.63E-06
ACTR1A	1.764311	1.331124	2.338469	7.82E-05	0.006804
WDR77	1.617611	1.27413	2.053686	7.84E-05	0.038898
DDX58	0.793446	0.707347	0.890027	7.88E-05	0.000372
PKN3	1.357887	1.166433	1.580765	7.97E-05	0.000277
CCR8	0.6784	0.559461	0.822624	7.97E-05	0.000145
CASP7	0.720175	0.611673	0.847925	8.15E-05	0.004238
TRAFD1	0.617883	0.486274	0.785111	8.16E-05	0.000252
PTGDR	0.509373	0.364154	0.712504	8.16E-05	0.000107
P2RX1	0.712725	0.602209	0.843522	8.17E-05	0.006302
MAN1A1	0.814089	0.734862	0.901857	8.24E-05	0.00058
TSHR	0.429012	0.2815	0.653824	8.27E-05	0.000346
LGALS7B	1.119039	1.05809	1.1835	8.28E-05	0.015503
SRD5A3	1.5533	1.247404	1.93421	8.30E-05	1.52E-05
ZNF749	1.246862	1.117071	1.391734	8.35E-05	0.0105
DMAC2	1.869682	1.368879	2.553702	8.36E-05	9.76E-05
ERFL	0.656893	0.532776	0.809924	8.39E-05	0.008298
GPR143	1.11196	1.054652	1.172382	8.46E-05	0.003238
RBM14	1.849936	1.361276	2.514011	8.47E-05	0.000251
DENND1C	0.7996	0.715221	0.893934	8.48E-05	0.001119
WDR62	1.433103	1.197684	1.714797	8.49E-05	8.14E-05
RAB37	0.772493	0.679157	0.878656	8.53E-05	8.09E-05
TUFM	1.683026	1.297987	2.182285	8.57E-05	0.000621
NECAB2	1.183202	1.087924	1.286824	8.59E-05	0.001524
SIRPD	0.199456	0.089212	0.445936	8.59E-05	0.007259
ADGRE5	0.745985	0.64448	0.863477	8.60E-05	1.32E-05
FGR	0.802885	0.719537	0.895887	8.64E-05	0.000154
DEXI	1.488088	1.220216	1.814765	8.66E-05	2.98E-06
CLEC12A	0.763848	0.667678	0.873869	8.72E-05	0.000533
PEAK3	0.616878	0.484588	0.785282	8.76E-05	0.000354
MCL1	0.68694	0.569383	0.828769	8.81E-05	0.001845
KLRG1	0.693956	0.578081	0.833058	8.87E-05	0.000817
SLC8A1	0.676194	0.555993	0.822383	8.92E-05	1.03E-05
KIR3DX1	0.154009	0.060399	0.392699	8.96E-05	1.46E-05
TMPRSS11I	1.316077	1.147087	1.509962	8.96E-05	0.029986
CEP85L	0.731431	0.625443	0.85538	9.01E-05	0.014291
CD14	0.840954	0.771113	0.917121	9.01E-05	0.000101
TRAF1	0.840097	0.769923	0.916666	9.04E-05	4.46E-05
MECR	1.722302	1.311897	2.261095	9.05E-05	0.020615
DHRS3	0.81626	0.737342	0.903624	9.10E-05	0.000433
DERL3	0.828122	0.753475	0.910165	9.12E-05	0.001668
TYRP1	1.061401	1.030179	1.093569	9.16E-05	0.001783
SLC43A2	0.745975	0.644063	0.864012	9.22E-05	0.000256
IL27RA	0.828328	0.753703	0.910343	9.23E-05	0.003245
TRIM5	0.72287	0.614323	0.850597	9.26E-05	0.000886
NAXE	1.409256	1.186553	1.673757	9.27E-05	0.000127
ARRB2	0.674223	0.553268	0.821621	9.32E-05	0.000318
P2RY8	0.805481	0.722647	0.897811	9.35E-05	0.000117

DNAJB9	0.738533	0.634305	0.859888	9.43E-05	0.013237
RGL4	0.570383	0.430298	0.756075	9.44E-05	0.003962
B4GALT2	1.568076	1.251	1.965516	9.50E-05	0.000529
RAC2	0.861662	0.799557	0.928591	9.58E-05	0.000918
SLC1A3	0.813945	0.733851	0.902781	9.82E-05	0.000522
CHI3L1	0.883738	0.830446	0.940449	9.83E-05	2.89E-05
VSIR	0.800623	0.715796	0.895504	9.97E-05	8.05E-05
CTB-60B18	1.249308	1.116731	1.397625	0.000101	0.006362
SIGLEC9	0.767222	0.671289	0.876865	0.000101	0.00023
VPS13C	0.757709	0.658805	0.871462	0.000101	0.002506
PSMD9	1.914638	1.379861	2.656671	0.000102	0.000448
SLC27A2	0.69219	0.574966	0.833315	0.000102	3.64E-05
IDO2	0.230785	0.110144	0.483563	0.000102	1.27E-06
SIGLEC14	0.823416	0.746496	0.908261	0.000103	3.26E-05
FERMT3	0.845816	0.777224	0.920462	0.000104	0.000259
CLNK	0.42454	0.275384	0.654483	0.000105	3.85E-06
UBAP2L	1.692697	1.297216	2.20875	0.000106	4.01E-05
PGGHG	0.798195	0.712139	0.89465	0.000108	0.004359
CCL2	0.836699	0.764504	0.915711	0.000108	0.000239
C4B	0.766931	0.670529	0.877194	0.000108	0.011194
POLR2A	1.578416	1.252785	1.988688	0.000108	0.002034
PKIG	0.73243	0.625525	0.857607	0.00011	0.00063
ICAM1	0.825621	0.749228	0.909805	0.00011	5.62E-06
DOCK11	0.790586	0.701834	0.890562	0.00011	0.008681
SH3BGRL	0.707512	0.593667	0.843189	0.000111	0.001528
PTGER4	0.793756	0.706001	0.892419	0.000112	0.001108
ARHGEF6	0.82187	0.743998	0.907892	0.000112	0.001212
MGAT5B	1.153128	1.072699	1.239586	0.000112	0.000198
PI3	1.099505	1.047763	1.153803	0.000115	0.043351
SYDE1	1.380883	1.172012	1.626979	0.000115	0.002178
SNTB1	0.824964	0.748109	0.909715	0.000115	0.023718
UBE2O	1.607991	1.262938	2.047317	0.000116	0.00569
C6orf132	1.249063	1.115483	1.398639	0.000116	0.004148
SMIM20	0.812632	0.731226	0.903101	0.000117	0.00738
PAQR7	1.510937	1.224542	1.864314	0.000119	0.005266
PNOC	0.766954	0.670028	0.8779	0.000119	2.92E-06
CCNF	1.441431	1.196472	1.736542	0.000119	0.000106
CCL3	0.821565	0.743243	0.908141	0.000121	0.00202
PTAFR	0.823818	0.746312	0.909373	0.000121	0.000252
ITGAM	0.80115	0.715519	0.897028	0.000121	5.61E-07
GPR132	0.774485	0.679818	0.882334	0.000122	0.00093
PLA2G4F	1.241736	1.111868	1.386773	0.000122	0.037264
SYTL3	0.764069	0.666001	0.876577	0.000123	0.001034
BAIAP2L2	1.175111	1.082184	1.276018	0.000124	0.002044
MPEG1	0.847024	0.778173	0.921966	0.000124	0.000312
AIMP2	1.56541	1.244891	1.968452	0.000126	0.001323
PNMA6A	1.174969	1.081984	1.275946	0.000127	0.009853
TMPRSS4	1.375863	1.168728	1.61971	0.000127	0.049599
PUS10	0.6268	0.493586	0.795967	0.000127	0.00521
SP4	0.732321	0.624434	0.858849	0.000127	0.005336
ZNRF2	0.700601	0.583988	0.840498	0.000128	0.005449

RNF166	0.684057	0.563246	0.830779	0.000128	0.000685
EEF2KMT	1.684829	1.289799	2.200845	0.00013	0.000232
DDX41	1.701917	1.296157	2.234699	0.00013	0.003046
ZNF438	0.697985	0.58057	0.839146	0.00013	0.011936
SAMD9	0.808381	0.7249	0.901477	0.000131	0.006354
CD163	0.851921	0.784751	0.92484	0.000131	0.000502
TPRN	1.390471	1.174249	1.646506	0.000132	0.003589
BICD2	1.562137	1.242537	1.963944	0.000134	0.012812
ADORA2A	0.365146	0.217722	0.612392	0.000134	0.016652
FBP1	0.841191	0.769727	0.919289	0.000135	7.25E-05
HLA-DQA2	0.900925	0.853927	0.950509	0.000135	0.011068
IPO13	1.576411	1.247621	1.991849	0.000137	0.000281
KMO	0.735795	0.628454	0.86147	0.000137	0.000153
EIF3B	1.6051	1.258403	2.047314	0.000138	0.012343
FOXN1	1.287033	1.130373	1.465404	0.000139	0.014452
OGFRL1	0.766588	0.668603	0.878932	0.000139	0.022678
NUP188	1.618275	1.263152	2.073237	0.00014	0.000405
TINCR	1.17035	1.079322	1.269054	0.00014	0.009718
COL22A1	0.901453	0.854564	0.950914	0.000141	0.006262
ZDHHC16	1.678182	1.285482	2.190847	0.000141	0.016842
GSAP	0.780998	0.687643	0.887027	0.000141	0.002269
CNTFR	1.132039	1.061987	1.206712	0.000142	0.010759
INSL3	0.650503	0.521267	0.811781	0.000142	0.00083
IFI44	0.843079	0.772105	0.920576	0.000142	0.011132
TRIM34	0.474997	0.323676	0.697061	0.000142	0.003596
ZMAT1	0.775019	0.679619	0.88381	0.000143	0.001755
IQCE	1.36645	1.163181	1.605241	0.000145	0.010025
PARVA	1.442003	1.193851	1.741735	0.000145	0.001886
DENND4A	0.724968	0.614086	0.85587	0.000146	0.011524
BCL7A	1.348265	1.155574	1.573088	0.000146	0.011885
CERS3	1.229637	1.105181	1.368108	0.000147	0.002253
LGALS2	0.829629	0.753345	0.913637	0.000147	0.006801
MYO1G	0.798493	0.710844	0.896949	0.000149	0.001505
VCAM1	0.842639	0.771248	0.920639	0.00015	0.000138
GSG1L	1.245531	1.111819	1.395325	0.000151	0.009308
TNF	0.753444	0.650783	0.872301	0.000152	0.004621
PIK3CG	0.795361	0.706502	0.895396	0.000152	0.000321
PLCL2	0.773541	0.677294	0.883466	0.000152	0.000209
CCDC194	0.589467	0.448365	0.774973	0.000153	5.55E-06
ARL6IP5	0.745943	0.640893	0.868211	0.000154	0.001323
TROAP	1.34336	1.152821	1.565392	0.000155	0.000313
ERMN	0.531473	0.382947	0.737605	0.000157	1.30E-05
LMO2	0.766939	0.668337	0.880088	0.000157	0.000184
CCND2	0.824598	0.746072	0.91139	0.000159	0.003433
CD37	0.829627	0.752993	0.91406	0.000159	3.79E-05
MILR1	0.802771	0.716248	0.899745	0.00016	5.08E-05
LAIR2	0.710971	0.595574	0.848727	0.00016	7.16E-05
SNX10	0.84536	0.774743	0.922414	0.00016	0.001585
CIAO2A	0.683068	0.560348	0.832666	0.000162	0.001702
ARHGEF4	1.242889	1.110087	1.391578	0.000162	0.000524
SH3PXD2B	1.261264	1.117911	1.422999	0.000163	0.000374

TLR7	0.795552	0.706336	0.896037	0.000164	0.000104
SIGLEC7	0.786339	0.69393	0.891054	0.000164	0.000723
CFAP36	0.736704	0.628375	0.863709	0.000166	0.041979
FGF2	0.838589	0.765157	0.919068	0.000167	0.000933
KIR3DL2	0.332311	0.187236	0.589794	0.000167	0.000141
C2	0.858989	0.793578	0.929792	0.000169	0.000612
SH2D1B	0.687725	0.565803	0.835917	0.00017	9.21E-05
ALOXE3	1.273562	1.122711	1.444681	0.00017	0.029229
EPHX3	1.189069	1.086406	1.301434	0.000171	0.015742
PSMB10	0.789633	0.698058	0.89322	0.000173	0.000241
KIF1C	1.568027	1.239727	1.983267	0.000175	2.28E-05
PMPCA	1.588417	1.247378	2.022699	0.000175	0.001814
NSDHL	1.603538	1.253013	2.052121	0.000175	0.001297
XXbac-BP(	0.435738	0.282325	0.672514	0.000176	8.74E-05
ARHGAP12	0.762966	0.662392	0.87881	0.000176	0.012489
ID2	0.785699	0.692623	0.891282	0.000178	0.000772
JAK3	0.82387	0.744406	0.911817	0.000181	0.004858
SELENOT	0.723074	0.61017	0.85687	0.000182	0.003059
GOT2	1.526363	1.223178	1.904699	0.000182	0.006033
CDC42SE2	0.720687	0.607094	0.855536	0.000182	0.000984
SAE1	1.603449	1.251978	2.053589	0.000184	6.92E-05
CFAP157	1.649019	1.268776	2.143217	0.000184	4.75E-05
KCTD21	1.365922	1.159969	1.608442	0.000184	0.013188
LPXN	0.806407	0.720405	0.902676	0.000184	0.003429
NME8	0.579065	0.43486	0.771091	0.000185	9.50E-05
FAS	0.810906	0.726512	0.905104	0.000185	0.001908
BLNK	0.799153	0.710503	0.898864	0.000186	3.16E-05
STK32C	1.372439	1.162281	1.620596	0.000189	0.003381
C1GALT1C1	0.661993	0.533005	0.822195	0.000191	0.0049
RILPL2	0.705885	0.587805	0.847685	0.000192	0.008997
POR	1.538623	1.226759	1.929767	0.000193	0.000144
MRPS12	1.532935	1.224499	1.919063	0.000194	5.40E-05
CCDC69	0.8248	0.745309	0.912769	0.000195	0.000471
GNAL	1.180574	1.081818	1.288346	0.000196	0.004723
KLK11	1.175211	1.079453	1.279463	0.000197	0.006776
KLRG2	1.563016	1.235513	1.977331	0.000197	0.000664
FETUB	1.455919	1.194673	1.774293	0.000197	0.00018
GORAB	0.763059	0.661749	0.879878	0.000199	0.011689
HELB	0.644997	0.511933	0.812648	0.000199	0.006933
FBXO6	0.799478	0.710544	0.899543	0.0002	0.00019
RPGR	0.722787	0.609115	0.857672	0.0002	0.003557
POU2F2	0.791225	0.69935	0.89517	0.0002	1.44E-05
GDPD5	1.250119	1.111307	1.406269	0.000201	0.001267
DGLUCY	0.680379	0.555329	0.833589	0.000202	0.010624
FGD3	0.794074	0.703125	0.896786	0.000203	0.003119
TLR4	0.823412	0.743144	0.91235	0.000205	2.64E-05
RNF114	0.624458	0.486987	0.800736	0.000206	0.004041
ULK1	1.565456	1.235401	1.98369	0.000207	0.000529
CA8	1.131769	1.060098	1.208286	0.000209	2.26E-06
SH3BP4	1.239438	1.106454	1.388405	0.00021	0.000841
MFNG	0.798192	0.708477	0.899267	0.000211	0.000199

ANKRD52	1.456077	1.193579	1.776305	0.000212	0.002557
TNFSF4	0.786427	0.692555	0.893023	0.000212	0.000253
LILRA5	0.801538	0.71299	0.901082	0.000212	0.007709
CXCL16	0.807715	0.721377	0.904386	0.000214	0.020269
NUCB2	0.788639	0.695466	0.894294	0.000214	0.000211
PPIF	1.454108	1.19261	1.772944	0.000214	0.000617
CP	0.78708	0.693332	0.893503	0.000215	4.20E-05
BTN3A2	0.803028	0.714937	0.901973	0.000215	0.000707
NUDT8	1.274735	1.120876	1.449714	0.000217	0.00312
ALPK1	0.737369	0.627429	0.866574	0.000217	0.0064
GPBAR1	0.661582	0.531496	0.823507	0.000217	0.025861
BYSL	1.477858	1.201518	1.817755	0.000217	5.48E-05
MAD1L1	1.32941	1.143127	1.546049	0.000218	0.006679
ODF3B	0.805194	0.717804	0.903222	0.000219	0.004113
RARRES1	0.844195	0.771676	0.923529	0.000219	0.000835
TXNRD2	1.483365	1.203365	1.828514	0.00022	0.007333
PTK6	1.198701	1.088812	1.31968	0.00022	0.002017
RP11-203J	0.648944	0.515894	0.816308	0.000221	0.021678
GPR183	0.831212	0.753535	0.916896	0.000221	0.00731
IDS	0.730592	0.618291	0.86329	0.000228	0.001132
OSCAR	0.813983	0.729521	0.908224	0.000231	2.41E-05
FXN	1.569079	1.234507	1.994326	0.000232	0.032528
CCRL2	0.757194	0.652961	0.878067	0.000233	0.000335
RNPEP	1.479561	1.200914	1.822863	0.000234	0.000127
ZDHHC11B	1.262269	1.114958	1.429043	0.000234	0.000446
IL7R	0.848327	0.777141	0.926033	0.000235	0.000122
HESX1	0.70469	0.584772	0.849199	0.000236	0.013547
TBC1D13	1.61476	1.250626	2.084916	0.000238	0.017531
FPR2	0.666136	0.536358	0.827315	0.000238	8.72E-05
NUTF2	1.646013	1.261797	2.147224	0.000238	0.029574
DBN1	1.31792	1.137248	1.527296	0.000243	0.002155
KLRC4	0.643118	0.508029	0.814129	0.000243	6.60E-07
KLK10	1.145056	1.065092	1.231022	0.000245	0.034363
ALG8	1.32399	1.13955	1.538282	0.000246	0.021231
EXOC3L4	0.717946	0.601373	0.857116	0.000247	0.000524
WDFY4	0.800944	0.711261	0.901934	0.000249	0.000899
SLC9A7	0.825033	0.744294	0.91453	0.000252	0.000244
VDAC1	1.604765	1.245617	2.067466	0.000253	8.71E-05
ATP1B1	0.85929	0.792221	0.932036	0.000255	0.009721
HAVCR1	0.242053	0.113171	0.517709	0.000255	4.42E-07
TAS1R3	0.690873	0.566493	0.842562	0.000261	0.000208
NCF2	0.82757	0.747601	0.916094	0.000262	0.000124
HROB	1.426449	1.178583	1.726445	0.000265	0.000221
SERPINA1	0.868769	0.805498	0.937009	0.000266	6.87E-06
SGPP1	0.731985	0.618906	0.865725	0.000268	0.001653
CBLN3	0.708685	0.588866	0.852885	0.000269	0.000978
UNG	1.526333	1.215758	1.916248	0.000269	0.000482
TIMM50	1.374213	1.158189	1.630531	0.00027	0.011832
SNX8	1.362969	1.153745	1.610135	0.000271	0.007703
ALG1	1.642908	1.257305	2.146771	0.000275	0.002031
ANXA2R	0.721122	0.604597	0.860104	0.000277	0.007963

CRABP2	1.12887	1.057438	1.205127	0.000279	0.001147
SERPINE2	0.873902	0.812622	0.939803	0.000279	0.000261
IFNL1	0.197565	0.082355	0.473947	0.000281	0.000123
NXT2	0.797715	0.706118	0.901194	0.000282	0.002647
HADHA	1.713585	1.281318	2.29168	0.000282	0.006274
WDR5	1.614448	1.246267	2.0914	0.000287	0.011114
SPRR3	1.13721	1.060852	1.219064	0.000288	0.015448
ARAP2	0.77975	0.681587	0.89205	0.00029	0.001751
SCPEP1	0.768808	0.6669	0.886289	0.00029	0.018273
TRIM56	0.70292	0.580886	0.85059	0.000291	0.030337
SDR9C7	1.208471	1.090757	1.338888	0.000293	0.002044
TAPBPL	0.776574	0.677218	0.890506	0.000294	1.32E-05
KCNMB1	0.661516	0.528893	0.827395	0.000295	2.26E-05
EPGN	1.307637	1.130837	1.512078	0.000296	0.01966
USP15	0.722365	0.605635	0.861593	0.000299	0.003956
IGFLR1	0.725423	0.609564	0.863303	0.0003	0.049853
VAMP1	0.762757	0.658581	0.883413	0.000301	0.016181
UQCRFS1	1.589648	1.236137	2.044256	0.000304	0.000782
ENDOG	1.40312	1.167535	1.686243	0.000304	0.007802
DDO	0.819137	0.73503	0.912868	0.000307	0.000352
MLXIP	1.505879	1.205705	1.880784	0.000307	0.009874
CD180	0.804477	0.714785	0.905424	0.000309	6.72E-07
SARAF	0.711088	0.590798	0.855869	0.000311	0.012448
IFI27	0.89611	0.84424	0.951166	0.000311	0.000185
WNT11	1.209971	1.090873	1.342072	0.000312	0.010466
GOLM2	0.798519	0.706554	0.902455	0.000313	0.009187
CCPG1	0.770392	0.66846	0.887867	0.000315	0.00152
MRC1	0.846545	0.773144	0.926914	0.000318	0.010625
SLC25A15	1.329	1.13831	1.551634	0.000319	1.58E-05
MEF2B	0.605086	0.460245	0.795508	0.00032	0.019034
CD244	0.783872	0.686495	0.895061	0.000321	0.000999
SNX1	0.718511	0.600107	0.860278	0.000321	0.043322
PIK3R5	0.786815	0.690465	0.89661	0.000321	0.00249
UTRN	0.796009	0.70292	0.901425	0.000324	0.001014
AGAP2	0.751123	0.642616	0.877952	0.000324	5.11E-05
TMEM35B	0.785051	0.688001	0.895791	0.000325	0.001552
ZEB1	0.826577	0.745019	0.917063	0.000326	0.003909
CD300A	0.818667	0.734021	0.913074	0.000327	0.000194
PHYKPL	0.660493	0.526751	0.828192	0.000327	0.010437
ZNF777	1.541996	1.217426	1.953098	0.000329	0.000284
STAMBPL1	0.799073	0.706944	0.903209	0.000332	0.037997
TIFA	0.721448	0.603574	0.862342	0.000334	0.009011
TM6SF2	1.353126	1.147013	1.596277	0.000335	0.013184
SLFN14	0.081779	0.020818	0.32125	0.000335	3.39E-06
RAC3	1.196162	1.084601	1.319198	0.000336	0.001582
NDUFC2	1.355985	1.148007	1.601643	0.000337	0.037734
IPO9	1.530873	1.21279	1.93238	0.000339	0.000598
ST6GAL1	0.817703	0.732466	0.912859	0.000339	0.001175
DUOXA1	1.197542	1.085075	1.321665	0.00034	0.005917
SLC3A2	1.344883	1.143568	1.581637	0.000342	0.000506
EIF3K	1.598573	1.236373	2.066881	0.000345	0.003819

SORD	1.316252	1.132225	1.530189	0.000349	0.018991
KPNA2	1.397105	1.163103	1.678184	0.00035	0.000907
ITM2A	0.855251	0.784998	0.931791	0.00035	4.05E-06
NYNRIN	1.179422	1.07739	1.291118	0.000351	0.000419
S100P	1.214022	1.091546	1.350241	0.000351	0.027304
MSR1	0.833	0.753573	0.920799	0.000352	0.000107
FBXO17	1.252586	1.107017	1.417297	0.000353	0.007047
PSMD8	1.614324	1.241282	2.099477	0.000354	0.012982
TOX	0.832971	0.753488	0.920838	0.000355	0.000498
PA2G4	1.610794	1.239753	2.092883	0.000359	0.000143
CD52	0.881437	0.822412	0.944699	0.000359	0.00065
FMNL2	0.840162	0.763519	0.924498	0.000359	0.005065
KRTAP19-1	1.092887	1.040842	1.147534	0.00036	0.000397
GRK2	0.657454	0.522151	0.827818	0.000361	0.001822
CALHM1	0.065324	0.014585	0.292566	0.000362	0.003742
PRXL2B	1.344632	1.142333	1.582757	0.000371	0.00981
FFAR4	0.541012	0.385748	0.75877	0.000372	5.87E-07
C5orf58	0.759671	0.652968	0.883811	0.000372	0.003244
FLVCR2	0.746162	0.635032	0.876739	0.000373	0.000318
CCL13	0.85001	0.777209	0.929631	0.000375	0.000116
RITA1	1.638467	1.248136	2.150867	0.000376	0.000994
TICRR	1.34487	1.142163	1.583553	0.000379	6.39E-05
AACS	1.427019	1.172872	1.736236	0.00038	0.004185
TNFSF14	0.692256	0.565116	0.848	0.000382	0.009175
XIRP1	0.743483	0.631288	0.875616	0.000383	0.000138
TNFRSF25	0.773645	0.671417	0.891438	0.000386	7.21E-05
APH1A	1.577267	1.22628	2.028713	0.000388	0.003979
P2RY12	0.794024	0.699041	0.901914	0.000388	0.000136
FLI1	0.792042	0.696332	0.900907	0.000388	0.001575
SLC38A7	1.538043	1.212487	1.951012	0.000389	0.021567
RNF19A	0.765649	0.66059	0.887417	0.000391	0.023621
OVOL1	1.224869	1.094954	1.370199	0.000392	0.009861
PKMYT1	1.287157	1.119478	1.479951	0.000393	0.002416
NR2F6	1.443172	1.178141	1.767825	0.000395	0.000491
WIZ	1.575672	1.225293	2.026244	0.000395	0.000122
CNOT6L	0.721191	0.601906	0.864116	0.000395	0.032352
MT2A	0.858821	0.789429	0.934314	0.000399	1.77E-05
DGCR6	1.313918	1.12933	1.528678	0.000408	0.008063
ATP2A3	0.848168	0.774148	0.929266	0.000408	0.004829
KIFC1	1.312186	1.128607	1.525624	0.00041	0.000117
SMIM4	1.383673	1.15555	1.656831	0.000411	2.79E-05
CPNE5	0.851856	0.779328	0.931133	0.000413	0.00088
ARNTL	0.666851	0.532496	0.835107	0.000416	0.00204
DDN	1.250415	1.104464	1.415654	0.000417	0.000181
FKBP4	1.385735	1.156086	1.661001	0.000417	0.006851
VSIG4	0.859438	0.790085	0.934878	0.000418	0.000192
NOP2	1.491075	1.194315	1.861574	0.000418	0.003645
TRAF7	1.557195	1.217556	1.991577	0.000419	0.000241
XCR1	0.740926	0.627225	0.875237	0.000419	5.44E-05
HNRNPUL1	1.752671	1.283155	2.393988	0.00042	0.009498
ARMC6	1.49787	1.196451	1.875226	0.000424	0.002526

SFRP1	0.931773	0.895853	0.969133	0.000426	0.001502
RUVBL2	1.445683	1.17762	1.774765	0.000428	0.011694
CPEB4	0.756603	0.647837	0.88363	0.000428	0.011393
FAH	1.320946	1.131352	1.542312	0.00043	0.001054
ACOXL	0.438094	0.27664	0.693776	0.000434	0.004305
ARHGAP18	0.79323	0.697201	0.902485	0.000434	0.017067
CLUH	1.419279	1.167344	1.725587	0.000445	5.35E-05
SLC38A8	1.127031	1.054222	1.204869	0.000449	3.98E-05
PSMA3	0.651084	0.512286	0.827487	0.000452	0.001749
OAS2	0.864304	0.796673	0.937677	0.000452	3.55E-05
ADCYAP1R1	1.347818	1.140771	1.592444	0.000452	0.023044
RABL6	1.499828	1.195828	1.881111	0.000452	0.000207
TPI1	1.458079	1.180924	1.80028	0.000455	0.015275
RREB1	1.606761	1.232581	2.094532	0.000455	0.000384
KDM4C	0.635203	0.492869	0.818641	0.000455	0.002341
MT1H	0.804436	0.712283	0.908513	0.000456	0.015495
PDE4B	0.827936	0.74497	0.920141	0.000457	0.00204
FHOD3	1.190258	1.079772	1.31205	0.000458	0.002585
WNK2	1.192011	1.080439	1.315105	0.00046	3.09E-05
TNFSF8	0.778934	0.677306	0.89581	0.000461	4.44E-05
CDCA3	1.379655	1.15225	1.651939	0.000462	4.31E-05
CH507-9B2	0.666483	0.53106	0.836438	0.000463	0.01442
CBLN2	1.231911	1.096111	1.384535	0.000465	0.011008
SIRPB1	0.764196	0.657311	0.888463	0.000468	0.005695
SHPK	1.479552	1.18785	1.842889	0.000472	0.012916
MITD1	0.713375	0.590319	0.862082	0.000472	0.023604
IGLL5	0.918971	0.876441	0.963564	0.000474	0.000757
PABIR3	0.586269	0.434525	0.791004	0.000476	0.003365
CLEC9A	0.650809	0.511457	0.82813	0.000476	0.000188
IBA57	1.536976	1.207552	1.956268	0.000479	0.001884
ACSM3	0.63467	0.491672	0.819256	0.000482	1.36E-07
SORL1	0.854558	0.782367	0.933411	0.000483	0.002873
ZNF574	1.5793	1.221792	2.04142	0.000484	0.000288
UMAD1	0.72535	0.605555	0.868844	0.000489	0.008951
EIF5A	1.543255	1.209153	1.969673	0.000491	0.016311
ITGB7	0.808302	0.717135	0.911059	0.000491	0.009576
GAB2	1.265156	1.108336	1.444166	0.000495	0.001478
MAST2	1.484833	1.188691	1.854755	0.000496	0.006792
KLRC4-KLF	0.608722	0.460361	0.804895	0.000496	2.13E-07
ATG9A	1.615587	1.233289	2.116392	0.000498	0.000343
PRAM1	0.750167	0.638083	0.881939	0.000498	0.000176
CGB7	2.443087	1.477412	4.039953	0.0005	0.049839
CSNK1E	1.477022	1.185604	1.840071	0.000505	0.00165
FPR1	0.834297	0.753321	0.923977	0.000506	0.000663
CIDEA	1.197688	1.081875	1.325898	0.000508	0.012527
TTC7A	0.715525	0.592469	0.864138	0.000508	0.02026
PLCB2	0.819652	0.732703	0.916918	0.000509	0.005684
UNC45A	1.589459	1.223238	2.065323	0.000524	8.55E-05
PPRC1	1.553658	1.21113	1.993059	0.000526	0.014255
SLC47A2	1.967772	1.342047	2.885239	0.000527	0.017609
BRD4	1.722756	1.266643	2.343114	0.000528	0.00272

GTF3C2	1.695915	1.258001	2.286268	0.000528	0.001403
SGK3	0.814352	0.725024	0.914685	0.000532	0.00227
GFI1	0.814576	0.72531	0.914827	0.000534	0.000114
OMA1	0.717709	0.594827	0.865976	0.000537	0.00112
ZNF695	1.373969	1.147727	1.644807	0.000538	0.000726
NFE2L3	0.845875	0.769361	0.929999	0.00054	0.002789
ATP6V0D1	1.499323	1.191876	1.886077	0.000542	0.00673
L3HYPDH	0.723491	0.602243	0.86915	0.000543	0.022352
SLC31A2	0.713837	0.5897	0.864106	0.000543	0.002086
MRPS6	0.766087	0.658576	0.891149	0.000553	0.004155
CAMSAP3	1.207766	1.085047	1.344364	0.000554	0.047289
IL10	0.761522	0.652395	0.888903	0.000556	2.62E-05
COL4A3	0.561494	0.404589	0.779248	0.000557	0.0006
BTK	0.823446	0.737412	0.919518	0.00056	0.000126
IP6K1	1.584814	1.219998	2.058722	0.000561	0.008607
SPRYD4	1.59691	1.223912	2.083581	0.000563	0.001075
NEUROG3	0.447394	0.283221	0.706732	0.000565	0.002438
TNFAIP2	0.833754	0.751849	0.924582	0.000568	0.005805
SLC12A3	0.358439	0.199966	0.642502	0.00057	0.000252
TMEM176A	0.841113	0.762263	0.92812	0.000571	0.000265
EIF6	1.546623	1.206799	1.982139	0.000571	0.02087
CCL7	0.808222	0.715908	0.912439	0.00058	0.002306
SUV39H1	1.54691	1.206497	1.983372	0.000581	0.000442
RBPJ	0.708158	0.581766	0.862009	0.000581	0.049888
ATP6AP2	0.683242	0.549921	0.848884	0.000583	0.018755
ITPR1	0.806869	0.713981	0.911842	0.000584	0.003912
OTX1	1.255411	1.102752	1.429204	0.000585	0.013991
ERAP1	0.783343	0.681543	0.900348	0.000586	0.011708
C4A	0.791384	0.692559	0.904312	0.000586	0.002431
YPEL4	0.748142	0.633992	0.882843	0.000592	0.024998
FAM174B	1.18712	1.076394	1.309237	0.000596	0.000216
EN2	1.188645	1.076973	1.311895	0.000597	0.043403
SLCO2B1	0.845319	0.767982	0.930444	0.000598	0.00355
TLCD3A	1.341252	1.134173	1.58614	0.0006	0.000878
FBL	1.430322	1.165864	1.754768	0.000601	2.06E-05
TBC1D8	0.75392	0.641576	0.885937	0.000601	0.000632
FCHO1	0.785871	0.684805	0.901851	0.000602	0.035623
NAAA	0.797456	0.700652	0.907635	0.000609	0.006278
ATG3	0.686	0.552994	0.850996	0.00061	0.010681
PLAC8	0.800808	0.705266	0.909293	0.00061	0.003886
CALCOCO2	0.67755	0.542311	0.846515	0.000611	3.21E-05
TMEM30A	0.7744	0.669019	0.89638	0.000613	0.02616
MVK	1.420795	1.162123	1.737043	0.000614	0.008995
MRPL21	1.413724	1.159611	1.723524	0.000615	0.010752
PLA2G1B	0.628994	0.482396	0.820143	0.000616	0.016785
PRODH	1.265979	1.106022	1.449068	0.000621	0.004491
CDCA8	1.30971	1.12215	1.528621	0.000623	0.001338
RP11-872I	1.333536	1.130808	1.572608	0.000624	0.002089
AKAP6	0.881482	0.82001	0.947562	0.000625	0.003558
UBE2J1	0.724571	0.602377	0.871553	0.000629	0.006195
FUT7	0.705525	0.577648	0.861712	0.000629	0.00339

FCRL2	0.730994	0.610752	0.874909	0.000632	1.20E-05
ATP11A	1.211551	1.085238	1.352566	0.000635	0.012432
SLC26A2	0.842158	0.763066	0.929449	0.00064	0.032656
FARP1	1.28302	1.111875	1.480509	0.000645	0.001804
BCAP29	0.783739	0.681357	0.901505	0.000646	0.037352
FMNL1	0.831797	0.748275	0.924642	0.000647	0.000824
CDC25A	1.297768	1.117263	1.507434	0.000647	0.000114
ANKS6	1.378705	1.146308	1.658218	0.00065	0.000618
MRPS11	1.680383	1.246841	2.264673	0.000652	0.001003
EPN1	1.543097	1.202457	1.980235	0.000653	0.003124
TEAD3	1.267364	1.10594	1.45235	0.000653	0.020114
GTSF1L	0.613804	0.463562	0.812741	0.000656	2.51E-06
PARP10	0.798733	0.701858	0.90898	0.000658	0.000986
GLRX	0.77798	0.673343	0.898877	0.000658	0.001137
LILRA1	0.711811	0.585336	0.865615	0.00066	3.66E-05
TMEM79	1.330952	1.128996	1.569035	0.000662	0.011686
SYNM	0.870816	0.804168	0.942989	0.000662	0.010386
CDC42BPG	1.205208	1.082422	1.341921	0.000662	0.005044
NARS2	1.2279	1.09099	1.381992	0.000665	0.000442
MAP6D1	1.26218	1.103802	1.443283	0.000665	0.009328
WNT1	0.390065	0.226753	0.670999	0.00067	0.002423
DCTPP1	1.534912	1.199052	1.964847	0.000672	0.00458
GRAP2	0.807176	0.713375	0.913312	0.000677	3.48E-05
MTCH2	1.574923	1.211769	2.046911	0.000683	0.005175
NUDT1	1.404363	1.15437	1.708494	0.000686	0.003371
KLRC2	0.721794	0.597897	0.871364	0.000691	2.58E-07
NOX4	0.852391	0.777265	0.934779	0.000692	0.003411
ERFE	1.205414	1.082091	1.342793	0.000692	0.018299
TNFRSF8	0.758059	0.645957	0.889614	0.000692	0.008948
GRHL3	1.223161	1.088754	1.374161	0.000695	0.008301
SDR16C5	1.21157	1.08424	1.353854	0.000705	0.003494
HMGB3	1.268942	1.105535	1.4565	0.000708	0.000744
PPP2R2B	0.761016	0.649724	0.891372	0.000711	7.53E-05
LILRA6	0.752012	0.637594	0.886964	0.000713	0.000813
CTPS1	1.379034	1.144815	1.661173	0.000714	0.000151
ELMO1	0.821886	0.733604	0.920791	0.000716	0.000531
PIPOX	0.589649	0.434208	0.800736	0.000716	2.11E-05
TFAP2A	1.157055	1.063291	1.259088	0.000716	0.009017
SPINK5	1.128548	1.052161	1.21048	0.00072	0.031376
SLC7A4	1.103818	1.042404	1.16885	0.00072	0.018014
CARM1	1.596784	1.217431	2.094345	0.000721	0.002513
TOGARAM2	0.334983	0.177701	0.631475	0.000722	0.001975
FLT3	0.678596	0.541979	0.849651	0.000724	0.000616
TMPRSS3	0.634619	0.487542	0.826065	0.000724	0.00134
JAG2	1.246598	1.097042	1.416541	0.000724	4.34E-06
AMD1	0.784222	0.681085	0.902978	0.000729	0.030854
PHETA1	1.365415	1.1397	1.635832	0.000729	0.000918
TIMP1	0.82212	0.733799	0.92107	0.00073	0.006009
DCTN2	1.497958	1.184864	1.893785	0.00073	0.000102
NMU	1.15515	1.062404	1.255992	0.000731	5.40E-05
CRLF3	0.735121	0.614865	0.878896	0.000735	0.011314

BNC1	1.196087	1.077941	1.327183	0.00074	0.016575
KBTBD8	0.736326	0.616364	0.879637	0.000743	0.000178
GSTO2	1.343263	1.131568	1.594563	0.000745	0.01445
HS3ST3B1	0.776631	0.670505	0.899555	0.000746	0.000295
KHSRP	1.563234	1.205583	2.026987	0.00075	0.007566
LCE2A	1.128689	1.051965	1.211009	0.000751	0.011657
SLC25A39	1.525663	1.193246	1.950686	0.000754	0.00106
MED12L	0.405607	0.239912	0.685737	0.000757	0.001545
PLD4	0.76987	0.661174	0.896435	0.000758	0.023465
CCDC7	0.549889	0.388228	0.778866	0.00076	0.027563
GHRL	0.625314	0.475687	0.822004	0.000766	0.014035
CD19	0.855501	0.781138	0.936943	0.000769	0.00015
TLR3	0.805718	0.710417	0.913804	0.00077	0.000191
GPR31	0.468749	0.301414	0.728983	0.000771	1.76E-06
MCEE	0.676914	0.539084	0.849984	0.000782	0.007181
OSGIN1	1.21347	1.083871	1.358566	0.000786	0.000288
CLIC4	0.806646	0.711524	0.914485	0.00079	0.010745
FBXW7	0.70085	0.569473	0.862537	0.00079	0.009122
PRRT4	1.148956	1.059405	1.246077	0.000797	0.00109
COL5A3	1.15273	1.060779	1.252652	0.000805	0.019661
HAMP	0.814432	0.72228	0.91834	0.000807	0.000362
POLE	1.425395	1.158434	1.753878	0.000808	3.30E-05
PARP8	0.82419	0.736029	0.922912	0.000809	0.009286
EGLN3	1.221233	1.086358	1.372852	0.000816	0.001624
ERMARD	0.692778	0.55877	0.858924	0.000818	0.044615
DHX58	0.803223	0.70648	0.913215	0.000819	0.008888
SLC29A4	1.192711	1.075742	1.3224	0.000819	0.000416
TMC8	0.849745	0.772417	0.934814	0.000824	0.002574
MGAT4A	0.79105	0.689461	0.907608	0.000831	0.00173
KHDRBS3	0.835271	0.751599	0.928258	0.000831	0.00256
TTC39A	1.127993	1.051063	1.210554	0.000832	0.016575
IFNAR1	0.710767	0.581717	0.868445	0.000839	0.048998
FPR3	0.860042	0.787212	0.93961	0.000839	0.001844
FOXRED2	1.295925	1.113031	1.508871	0.000839	0.003577
MYO10	1.20935	1.081688	1.352078	0.000839	0.004125
IL33	0.866432	0.796481	0.942527	0.000844	0.009329
GTF3C5	1.493325	1.179936	1.889949	0.000848	0.005146
NME4	1.393	1.146454	1.692566	0.000852	0.014935
TAP2	0.793795	0.693051	0.909183	0.000853	0.003276
COL4A4	0.73379	0.611734	0.8802	0.000854	0.002773
RHOF	0.635477	0.486792	0.829576	0.000856	0.002058
GAL	1.126143	1.050157	1.207626	0.000859	0.000172
HVCN1	0.816095	0.724164	0.919698	0.00086	0.013914
ZNF75D	0.740199	0.620166	0.883464	0.000861	0.017523
RNASE2	0.815111	0.722748	0.919278	0.000863	0.00233
FRMD3	0.810029	0.715584	0.91694	0.000866	0.026956
RETSAT	1.282343	1.107765	1.484432	0.000866	0.000268
ZDHHC9	1.428209	1.157921	1.761588	0.000869	0.008172
SLC9A9	0.803038	0.705663	0.91385	0.000881	0.00099
ZFAND6	0.71412	0.585598	0.870851	0.000881	0.00355
PGAM1	1.45078	1.165081	1.806538	0.000883	0.008087

EPPK1	1.372186	1.138673	1.653586	0.000886	0.000702
KDM7A	0.776321	0.668662	0.901312	0.000887	0.035601
DOLK	1.538648	1.193429	1.983728	0.000887	0.004439
OPN4	1.825384	1.28007	2.603003	0.000888	0.000856
GPR82	0.654041	0.509087	0.840268	0.000896	0.003197
APOBR	0.814146	0.721073	0.919232	0.000901	0.003667
PKD1L3	0.091044	0.022117	0.374786	0.000902	0.002309
NUP214	1.626916	1.220553	2.168572	0.000903	0.005197
KIF19	0.532216	0.366726	0.772385	0.000903	0.001694
CSF3R	0.797729	0.698013	0.911689	0.00091	0.00017
SUSD6	0.713859	0.584939	0.871192	0.00091	4.37E-05
BCL7C	1.537088	1.192218	1.981719	0.000912	0.011048
COX6B1	1.498269	1.179664	1.902925	0.000918	0.006111
MON1A	1.584351	1.206885	2.079874	0.000919	0.000616
MAL2	1.140421	1.055117	1.232621	0.000925	0.002223
PPM1H	1.204464	1.07887	1.344679	0.000929	0.006525
IRF7	0.833732	0.748591	0.928557	0.000938	0.001162
NME1	1.391317	1.144036	1.692047	0.000941	0.006143
SCAF4	1.605751	1.212837	2.125956	0.000941	0.028144
RELB	0.755008	0.639181	0.891823	0.000942	0.001336
DLG3	0.798919	0.69933	0.912689	0.00095	0.001908
FAT2	1.176852	1.068387	1.296327	0.000964	0.00269
MEAK7	1.531355	1.188981	1.972317	0.000965	0.037626
HNRNPAB	1.561327	1.198352	2.034245	0.000966	0.006707
IMPA2	1.245746	1.093237	1.419532	0.000974	0.006315
SLC25A51	1.471871	1.169681	1.852133	0.000978	0.013817
DIS3L2	1.743887	1.252801	2.427474	0.000982	0.001499
LOXL3	0.898026	0.84238	0.957349	0.000983	0.031204
LYN	0.825198	0.736088	0.925095	0.000983	0.004771
NTN4	0.849632	0.771068	0.9362	0.000996	0.028818
TTC16	0.539423	0.373492	0.779072	0.000998	0.005561
DNAH1	0.718563	0.590132	0.874946	0.001003	0.005708
CXorf38	0.689542	0.552485	0.8606	0.00101	0.017977
LMBRD2	0.783053	0.676789	0.906001	0.001014	0.013545
UAP1L1	1.339373	1.125162	1.594366	0.001015	0.031691
GAREM2	1.214445	1.081566	1.363649	0.001015	0.000445
POLR1A	1.485684	1.173159	1.881464	0.001019	0.025093
CREG1	0.789832	0.686073	0.909284	0.001026	0.000841
SELENOK	0.686382	0.548242	0.859328	0.00103	0.000148
GCN1	1.484001	1.172297	1.878584	0.001033	0.00065
SMCO4	0.810804	0.715313	0.919041	0.001036	0.000146
PLK1	1.264507	1.099016	1.454919	0.001041	0.000116
TULP3	1.379259	1.138062	1.671575	0.001043	0.008385
TC2N	0.842191	0.759996	0.933275	0.001046	0.000298
UCK1	1.408638	1.147663	1.728957	0.001048	0.01749
FANCE	1.372933	1.135677	1.659755	0.001059	0.001862
ZDHHC11	1.386775	1.140167	1.686723	0.001064	0.012262
APIP	0.697187	0.561729	0.865311	0.001066	0.02192
DAGLB	1.666319	1.227208	2.262549	0.001068	0.013155
RTL8A	1.429185	1.153729	1.770405	0.001079	0.00043
PGAM5	1.472077	1.167421	1.856236	0.001082	1.97E-05

MMP25	0.779406	0.671175	0.905089	0.001086	0.003325
TPPP	1.151297	1.057986	1.252837	0.001087	0.01397
TFAP2C	0.903753	0.850505	0.960335	0.00109	0.003955
BCL11B	0.82015	0.728139	0.923789	0.001092	4.40E-05
OR52N4	0.468107	0.296793	0.738307	0.001095	1.13E-05
CCR3	0.130643	0.038498	0.443336	0.001096	0.001281
UTS2R	0.376348	0.209267	0.676825	0.0011	0.000414
KCNA7	1.718552	1.241251	2.379391	0.001107	0.021691
BAIAP2	1.333354	1.121603	1.585081	0.001112	0.003935
C1orf116	1.185848	1.070254	1.313926	0.001124	0.019756
PYROXD2	0.885243	0.822634	0.952618	0.001126	0.027197
PRCC	1.550591	1.190741	2.019191	0.001131	0.00222
FAM184A	0.760185	0.644505	0.896627	0.001132	0.000226
RBBP8NL	1.468848	1.165265	1.851523	0.001135	0.026765
TBC1D10B	1.749703	1.249129	2.450875	0.001139	0.047365
MBD2	0.664093	0.518952	0.849826	0.001141	0.013149
ECEL1	0.72315	0.594852	0.879118	0.001142	0.000787
ATP5MC1	1.435133	1.154256	1.784358	0.00115	0.028712
PPP1R14B	1.33289	1.12087	1.585014	0.001151	0.027053
ASPHD2	0.755806	0.638334	0.894896	0.00116	0.000129
PACSIN1	0.702352	0.56749	0.869263	0.001162	0.00189
PLEKHO1	0.807792	0.710157	0.918849	0.001164	0.002359
ESRP1	1.151435	1.057488	1.253729	0.001166	0.000467
FERMT1	1.187879	1.07063	1.317969	0.001166	0.003406
TRIP13	1.25638	1.09469	1.441953	0.001166	0.017586
CEACAM5	1.266182	1.098028	1.460087	0.001169	0.016675
S100A2	1.113741	1.043607	1.188588	0.00117	0.005458
GLCCI1	0.796675	0.694505	0.913875	0.00117	0.007558
SLC66A3	0.7522	0.633365	0.893333	0.001172	0.000384
FOXN2	0.79944	0.69835	0.915164	0.001174	0.035194
CASP10	0.783228	0.675753	0.907796	0.001176	0.00166
DTL	1.296019	1.108098	1.515809	0.001178	2.72E-06
NIPAL3	0.820697	0.728315	0.924798	0.001182	0.036695
ZDHHC23	0.785015	0.678184	0.908676	0.001182	0.0013
C1D	0.704487	0.570015	0.870684	0.00119	0.022925
RAB40C	1.580512	1.198234	2.084749	0.001195	0.006465
GSK3A	1.695733	1.231896	2.334214	0.001199	0.000684
MPDU1	1.546594	1.187773	2.013812	0.001205	0.000733
CACFD1	1.334768	1.120693	1.589737	0.001206	0.001016
CENPO	1.394889	1.140094	1.706628	0.001221	0.001808
SRCAP	1.589528	1.200114	2.1053	0.001228	0.038202
A2M	0.863344	0.789688	0.94387	0.00124	0.008336
SKAP1	0.873142	0.804118	0.948091	0.001244	0.00035
NCK1	0.733742	0.608021	0.885458	0.001244	0.049984
ABCF1	1.465238	1.161944	1.847698	0.001245	0.001349
ISG15	0.878478	0.812022	0.950373	0.001246	0.003812
GINS2	1.32257	1.116087	1.567255	0.001246	0.010173
FOXP3	0.818984	0.725442	0.924587	0.001251	0.001237
FNDC3B	0.779112	0.669513	0.906652	0.001251	0.035125
BET1	0.720364	0.590243	0.879171	0.001252	0.008187
STARD4	0.842156	0.758705	0.934787	0.001253	0.002425

RCC1	1.359743	1.128154	1.638873	0.001256	0.001709
RAB8B	0.787272	0.680775	0.91043	0.001258	0.02992
IFIT1	0.866421	0.794092	0.945338	0.001265	0.007992
DBH	0.66233	0.51557	0.850868	0.001266	0.017473
LSM4	1.484283	1.167342	1.887277	0.001271	0.006693
RBM47	0.811385	0.714502	0.921404	0.001274	8.70E-05
ARHGAP15	0.829091	0.739721	0.929259	0.001279	2.70E-05
PITRM1	1.336651	1.120056	1.595131	0.001296	0.00238
CXCR4	0.850976	0.771236	0.938959	0.001306	0.018369
TUBB2A	1.277274	1.100132	1.48294	0.001315	0.008459
CYFIP2	0.848427	0.767445	0.937955	0.001321	0.034599
GPR155	0.863157	0.789004	0.944279	0.001323	0.00717
SPC24	1.32345	1.115354	1.570371	0.001324	0.00908
PDE3A	1.138226	1.051701	1.231869	0.001329	0.027485
SPNS3	0.691824	0.552404	0.866433	0.001334	0.011882
PMEL	1.06143	1.02347	1.100798	0.001334	0.024389
AEN	1.325451	1.115863	1.574406	0.001335	0.015888
RRP1	1.501646	1.171299	1.925162	0.00134	0.002391
CIBAR2	0.627453	0.471914	0.834258	0.001343	2.82E-05
SGCE	0.862209	0.787483	0.944026	0.001349	0.00105
IGIP	0.792075	0.686822	0.913457	0.001354	0.039873
CEP120	0.773181	0.660607	0.904939	0.001355	0.014641
TRIM28	1.430748	1.149214	1.781251	0.001355	1.30E-06
KLHL6	0.82738	0.736783	0.929118	0.001362	0.00019
SHISAL2A	0.716978	0.584888	0.878898	0.001363	0.000116
EMP3	1.284431	1.101978	1.497093	0.001364	0.011494
CEP89	1.534279	1.180524	1.994041	0.00137	0.015829
PRELID1	1.597512	1.198942	2.128581	0.001379	0.001118
ZNF667	1.205984	1.075209	1.352665	0.001383	0.001748
LSM2	1.455865	1.156501	1.83272	0.001384	0.000389
EDEM1	0.782097	0.672702	0.909281	0.001388	0.040602
ERAL1	1.639428	1.210803	2.219788	0.001389	0.000399
DIPK1B	1.177603	1.065294	1.301751	0.001389	0.015164
TRPM1	1.08536	1.032195	1.141264	0.001391	0.003097
IRX2	1.159323	1.058818	1.269368	0.001397	0.007229
ERI3	1.5984	1.198789	2.13122	0.001397	0.046427
CNOT9	1.623637	1.205647	2.186542	0.001416	0.003512
C5AR2	0.582124	0.417467	0.811726	0.001425	0.02724
PHEX	0.761226	0.643715	0.900188	0.001427	0.000559
HPS6	1.534724	1.179461	1.996996	0.001429	0.024762
TUBB4A	1.10659	1.039756	1.177719	0.00144	6.56E-06
METTL7B	0.870174	0.798828	0.947892	0.001442	0.006229
DPEP2	0.775768	0.663535	0.906986	0.00145	0.002336
SLC7A5	1.173773	1.063527	1.295447	0.001453	0.004979
SMIM12	1.68282	1.221439	2.318482	0.001455	0.013576
DLST	1.532942	1.17831	1.994307	0.001461	0.005827
FSTL4	1.24358	1.087331	1.422282	0.001462	0.005917
ZCCHC4	0.700761	0.562898	0.87239	0.001466	0.013978
SLC7A1	1.270165	1.096155	1.471799	0.001466	0.000176
SOWAHB	1.540479	1.180238	2.010675	0.001476	0.003678
SPATA1	0.474493	0.299661	0.751328	0.001477	0.007064

BATF3	0.778113	0.666598	0.908283	0.001478	0.001454
TLR10	0.787246	0.679233	0.912436	0.001488	1.57E-06
TARBP2	1.468196	1.158435	1.860786	0.001491	0.000308
JPH2	1.25775	1.091774	1.448958	0.001493	0.038359
SNAPC5	0.67236	0.526193	0.859131	0.001504	0.045069
URM1	1.435449	1.148241	1.794498	0.001506	0.027857
MS4A7	0.848847	0.767124	0.939276	0.001509	0.038824
FAM9B	2.51894	1.422815	4.459511	0.001525	0.038905
ADAM11	1.155733	1.056777	1.263956	0.001529	0.023262
DEPTOR	0.847044	0.764392	0.938633	0.00153	0.031051
CMKLR1	0.848033	0.765825	0.939065	0.001533	0.000653
SIGLEC5	0.588562	0.424014	0.816965	0.001533	0.006057
CR1L	0.588059	0.423336	0.816877	0.001545	0.000173
DYNC2I2	1.301425	1.105495	1.532079	0.001552	0.00035
NUDT14	1.288737	1.101379	1.507967	0.001553	0.000821
UQCRC1	1.44749	1.151157	1.820105	0.001554	0.001814
EFTUD2	1.630109	1.204367	2.20635	0.001556	0.005735
SNX6	0.777676	0.665505	0.908753	0.001557	0.03389
OPA3	1.624024	1.202585	2.193154	0.001559	0.001276
SMAP2	0.714919	0.580702	0.880159	0.00156	0.00261
ADAM8	0.846548	0.763526	0.938598	0.00156	0.000172
ILVBL	1.265321	1.093552	1.464071	0.00157	0.005658
TBXAS1	0.836312	0.748578	0.934329	0.001571	0.002742
CCNE1	1.294558	1.103058	1.519303	0.001573	0.016744
PTPRF	1.159901	1.057957	1.271669	0.001576	0.034876
ORC1	1.274796	1.096596	1.481954	0.001576	3.79E-05
CR1	0.758853	0.639472	0.900521	0.001579	0.000514
CD28	0.808725	0.708928	0.92257	0.001582	0.000532
KLHDC1	0.721968	0.589843	0.883689	0.001583	0.017301
ATXN1	0.739834	0.613645	0.891973	0.001588	0.005859
CERKL	0.855177	0.776046	0.942376	0.001589	0.000191
RAN	1.525829	1.173702	1.983599	0.001597	0.000191
OFD1	0.761631	0.643138	0.901956	0.0016	0.003773
SMIM14	0.758963	0.639385	0.900905	0.001616	0.020379
CREB3L1	1.147427	1.05341	1.249834	0.001617	0.00011
TRIM45	1.290072	1.101006	1.511604	0.001632	0.011297
CAPNS1	1.536396	1.176107	2.007055	0.001635	0.01344
STX4	0.609827	0.448231	0.829682	0.00164	0.002973
PDE6B	0.767959	0.651562	0.90515	0.001642	0.011762
VTCN1	1.414603	1.139839	1.755601	0.001645	0.011108
ITPKB	1.170311	1.061109	1.290752	0.001651	0.002637
TMPRSS13	1.132947	1.048176	1.224574	0.001657	0.02614
FCN3	0.784447	0.674322	0.912557	0.001658	0.008298
TXNL4A	1.486968	1.161211	1.904112	0.001663	0.001382
PTGES2	1.45029	1.150298	1.828518	0.001665	0.017057
CINP	1.529283	1.173419	1.993069	0.00167	0.038348
UCP2	0.848928	0.766506	0.940213	0.001672	0.005493
NOLC1	1.392881	1.132844	1.712608	0.001672	0.015301
SOWAHD	0.7963	0.69085	0.917846	0.001674	0.000575
PSMD3	1.67927	1.215056	2.320836	0.00169	0.015017
DLL3	1.101075	1.036836	1.169294	0.001693	0.000505

FIZ1	1.612442	1.196461	2.173051	0.0017	0.006357
IKZF2	0.751027	0.62805	0.898083	0.0017	0.006421
TRAIP	1.37132	1.12585	1.670309	0.001702	0.000216
LY86	0.851672	0.770364	0.941562	0.001712	6.58E-05
PLCD3	1.269637	1.093608	1.473999	0.001718	0.002465
ESPL1	1.291309	1.100435	1.51529	0.001732	0.000103
KRT19	1.136399	1.048924	1.231169	0.001756	0.027628
TECPR2	1.333538	1.113435	1.597151	0.001763	0.024279
DPM2	1.39991	1.133553	1.728854	0.001783	0.000142
NXPE3	0.806813	0.705146	0.923138	0.001785	0.011146
ABR	1.304192	1.103905	1.540818	0.001796	0.010412
RPS2	1.36061	1.121444	1.650782	0.001796	0.01919
UBE2W	0.744576	0.618721	0.896031	0.001796	0.023697
CIZ1	1.513083	1.16663	1.962422	0.001799	0.004412
MTHFD1L	1.27887	1.095852	1.492454	0.001799	0.00043
MS4A1	0.881913	0.814994	0.954326	0.001802	6.42E-05
FRA10AC1	0.710884	0.573712	0.880853	0.00181	0.000178
FAM3C	0.802131	0.698371	0.921307	0.001811	0.033579
OSGIN2	0.744237	0.618175	0.896006	0.001811	0.020429
IL12A	0.67997	0.533561	0.866554	0.001823	3.90E-07
ZNF721	0.766275	0.648155	0.905922	0.001829	0.033127
AJUBA	1.272628	1.093593	1.480973	0.00183	0.010874
GLRX5	1.537033	1.172945	2.014135	0.00183	0.003074
PHF23	1.544215	1.174934	2.02956	0.001833	0.017043
KRBA1	1.342587	1.11541	1.616034	0.001841	0.01377
DNER	1.119307	1.042632	1.201621	0.001852	0.044998
GPR25	0.530718	0.356152	0.790847	0.001852	1.51E-05
IL3RA	0.780016	0.667034	0.912134	0.001859	0.006731
HDAC4	1.358659	1.120085	1.648049	0.001864	0.004849
BEND3	1.415246	1.136959	1.761648	0.001877	0.003043
AURKB	1.244797	1.084291	1.429061	0.001878	0.020756
SIGLEC8	0.833104	0.742497	0.934768	0.001882	0.000232
TSPAN13	0.872006	0.799851	0.950671	0.001884	0.000231
IL18R1	0.769418	0.652081	0.907869	0.001904	0.000683
VAT1	1.273543	1.093252	1.483566	0.001905	0.001415
PTBP1	1.668643	1.207736	2.305447	0.001907	0.006957
LYSMD2	0.792953	0.68491	0.91804	0.001908	6.90E-05
ISG20	0.850644	0.768053	0.942117	0.001908	0.001009
TOX2	0.848233	0.764504	0.941133	0.001908	9.93E-05
SNAI3	0.75735	0.635415	0.902685	0.001915	0.000992
CUL2	0.787994	0.677893	0.915977	0.001916	0.04966
TSEN34	1.547989	1.174576	2.040114	0.00192	0.035877
GEMIN4	1.434616	1.142027	1.802168	0.001928	0.025919
TGM2	0.855926	0.775771	0.944364	0.001929	0.023067
SLC15A3	0.858774	0.779926	0.945594	0.001945	0.000614
AXL	0.872379	0.800182	0.951091	0.00195	3.68E-05
CDC20	1.235872	1.080882	1.413087	0.001951	0.003313
WARS1	0.861431	0.783843	0.946698	0.001952	4.97E-05
MRAP2	0.870984	0.798082	0.950546	0.001954	0.001456
MDFI	1.097291	1.034652	1.163722	0.001962	0.030386
CYTL1	1.093004	1.033167	1.156307	0.001963	0.001291

CHAF1A	1.350764	1.116557	1.634098	0.00197	0.001704
C5orf15	0.76356	0.6436	0.905879	0.001978	0.007035
GALNT2	1.264909	1.089878	1.46805	0.001984	0.001707
COMMD3	0.711934	0.57401	0.882999	0.001985	0.01951
KDELR1	1.582478	1.182875	2.117075	0.001995	0.030976
MRPL27	1.450091	1.145561	1.835575	0.002003	0.011242
CDK4	1.290112	1.097616	1.516366	0.002004	0.000316
ARHGAP27	0.77659	0.661477	0.911736	0.00201	0.015481
ILDR1	0.598531	0.432119	0.82903	0.002015	0.001969
ABTB1	0.736355	0.606354	0.894228	0.002015	0.015181
PLA2G7	0.86832	0.793877	0.949744	0.002019	0.010917
TBC1D8B	0.789607	0.679596	0.917427	0.00203	0.001265
MAP3K8	0.797866	0.691216	0.920971	0.002039	0.000458
TMEM11	1.521088	1.1652	1.985676	0.002041	0.03361
SFMBT2	0.803769	0.699555	0.923508	0.002049	0.037225
SERPINB7	1.152377	1.05298	1.261156	0.002058	0.004178
C16orf74	1.152523	1.053007	1.261444	0.002063	0.001856
FHAD1	0.790055	0.680064	0.917835	0.002064	0.001448
ALDH3B2	1.084632	1.029975	1.142189	0.002073	0.040545
RAB5B	1.357531	1.117453	1.649189	0.002081	0.004273
DUOX1	1.159981	1.055383	1.274946	0.002084	0.027066
KCNE1	0.517086	0.339746	0.786994	0.002086	7.49E-05
EARS2	1.468846	1.149764	1.876481	0.002093	0.001646
BTG2	0.826069	0.73136	0.933041	0.002102	0.026097
RAB5C	1.57692	1.179462	2.108315	0.002113	0.001492
PTTG2	0.324469	0.158299	0.665068	0.002113	0.006183
MRPL36	1.478271	1.152142	1.896715	0.002115	0.003483
FLG2	1.144768	1.05018	1.247876	0.002121	0.03823
ADGRE1	0.773758	0.656959	0.911322	0.002125	9.22E-05
CCDC170	0.680158	0.531874	0.869783	0.002127	0.002426
IGLL1	0.699229	0.5565	0.878564	0.002131	0.000449
RAPGEFL1	1.244339	1.082298	1.430641	0.002134	0.013058
KPNA5	0.783443	0.670432	0.915503	0.002135	0.02675
SELENOM	0.876266	0.805384	0.953385	0.002147	0.020836
SPRED1	0.837346	0.74758	0.937892	0.002153	0.032125
NT5M	1.249732	1.083844	1.441011	0.002155	0.019171
LSP1	0.864529	0.787743	0.9488	0.002159	0.004204
UBAP2	1.424509	1.13623	1.785929	0.002162	0.000436
ATP5MC2	1.457235	1.145498	1.853808	0.002169	0.004195
EPB41L2	0.820586	0.723141	0.931161	0.002171	0.00108
SAMD4B	1.501434	1.157876	1.946929	0.002172	0.004833
LOXL4	0.934877	0.895482	0.976005	0.002172	1.72E-05
TRIM27	1.485765	1.153494	1.913749	0.002172	4.62E-05
EPS15L1	1.634834	1.193879	2.238653	0.002177	0.048893
TIMELESS	1.336685	1.110252	1.609298	0.002181	0.000294
DZIP3	0.770576	0.652217	0.910412	0.002191	0.014315
ARID4B	0.801715	0.695987	0.923504	0.002192	0.034209
STAT3	0.716683	0.579028	0.887063	0.002204	0.018514
MLXIPL	0.730323	0.597214	0.8931	0.002204	0.003315
ZNF148	0.788069	0.676478	0.918068	0.002234	0.030067
TMEM120B	1.535736	1.16621	2.02235	0.002252	0.001758

IPO4	1.417625	1.133145	1.773524	0.00226	0.010783
CCNB1	1.304929	1.099953	1.548102	0.002268	0.009897
GPR146	1.360479	1.116485	1.657795	0.002269	0.010971
BCAS4	0.806705	0.702757	0.926027	0.002274	0.022163
DUSP14	1.334088	1.108618	1.605415	0.002276	0.002057
TM6SF1	0.780158	0.66514	0.915065	0.002283	0.016255
HHAT	1.229332	1.076451	1.403924	0.00231	0.000225
SNRNP25	1.433586	1.137133	1.807326	0.00231	0.046592
PI4KB	1.502644	1.156361	1.952623	0.002312	0.006441
CARHSP1	1.396798	1.126581	1.731828	0.002315	0.030358
GIPC1	1.407529	1.129638	1.753781	0.002317	0.000801
ZNF135	1.221881	1.074062	1.390044	0.002319	0.000526
CAMSAP1	1.422589	1.133844	1.784866	0.002325	0.006846
TIMM13	1.359569	1.115626	1.656853	0.002331	0.000479
PPM1K	0.827612	0.732652	0.934879	0.002343	0.024302
KIR3DL1	0.304842	0.141783	0.655432	0.002353	0.003601
CCR7	0.882472	0.814152	0.956525	0.002357	0.011449
MAPK7	1.524334	1.1615	2.000512	0.002371	0.0172
HDHD5	1.387911	1.123443	1.714636	0.002373	0.000283
LGMN	0.799675	0.692292	0.923713	0.002377	0.001933
CCDC71L	0.789623	0.677997	0.919627	0.002386	0.025453
TIMM22	1.561568	1.171235	2.081986	0.002389	0.002886
CIR1	0.741611	0.61142	0.899523	0.002405	0.000829
AKAP7	0.746982	0.618708	0.901852	0.002408	0.03738
FAM214A	0.776591	0.659581	0.91436	0.00241	0.035368
PTPN23	1.456492	1.142378	1.856977	0.002414	0.010994
IGDCC4	1.152729	1.051548	1.263644	0.002427	0.016211
RBM24	1.275047	1.089717	1.491896	0.002429	0.015076
GBP7	0.753401	0.62737	0.904751	0.002432	0.000342
MARS2	1.337441	1.108225	1.614065	0.002435	0.012402
SH2D2A	0.846019	0.759305	0.942635	0.00244	0.005717
ZDHHC13	0.762946	0.640411	0.908927	0.002454	0.017029
FDX2	1.498435	1.153349	1.946772	0.00246	0.009073
LIG1	1.381107	1.12045	1.702403	0.00248	0.0015
SEMA4A	0.81995	0.721006	0.932472	0.002482	0.030565
CYRIA	0.829117	0.734321	0.936151	0.002486	0.045243
CCDC120	1.376819	1.119121	1.693856	0.002491	0.001017
PEPD	1.415674	1.130067	1.773466	0.002498	0.000797
PARD6G	1.260596	1.084853	1.46481	0.002502	0.023353
DTX4	1.147658	1.04962	1.254854	0.002504	0.047138
KAT2B	0.84494	0.757473	0.942507	0.002511	0.015378
SLC15A1	1.220742	1.07249	1.389488	0.002533	0.022589
TVP23A	0.612928	0.446068	0.842205	0.002535	0.033331
SUPT5H	1.655644	1.193451	2.296833	0.002537	0.018575
ZNF71	1.325746	1.103914	1.592155	0.002543	0.000634
SEPSECS	0.748422	0.619989	0.90346	0.002554	0.048419
TUBB	1.369034	1.116244	1.679072	0.002563	0.017691
SHMT2	1.351718	1.111272	1.64419	0.002564	0.011096
PSEN2	1.235866	1.076913	1.41828	0.002571	0.001562
SIGMAR1	1.320734	1.102197	1.582601	0.002576	0.021306
LDB1	1.403341	1.125826	1.749263	0.002577	0.015285

IDUA	0.827193	0.731164	0.935834	0.002584	0.007108
PFAS	1.354219	1.111675	1.649683	0.002601	0.00272
BCL3	0.808959	0.704658	0.928699	0.00261	0.001969
GREB1	1.142696	1.04763	1.246388	0.002613	0.005167
IRX5	1.210074	1.068776	1.370053	0.002614	0.004765
ITGA6	0.874818	0.80185	0.954426	0.002615	0.032848
MAP4K1	0.854419	0.771161	0.946666	0.002632	0.023705
APOLD1	1.181129	1.059672	1.316507	0.00264	0.018747
EGR2	0.840831	0.750973	0.941442	0.002644	0.007156
ATF7	1.625908	1.184261	2.232257	0.002649	0.023642
ATG4D	1.431057	1.132794	1.807853	0.002651	0.001825
PDCD11	1.357495	1.11215	1.656964	0.002655	0.005181
PROX1	0.830671	0.735929	0.93761	0.002677	0.022079
C20orf27	1.355269	1.111306	1.652789	0.00268	0.000161
TLK1	0.770925	0.650476	0.913678	0.002687	0.005636
PLCG2	0.808219	0.703266	0.928835	0.002698	0.008509
KBTBD3	0.712578	0.571069	0.889152	0.002699	0.01648
NDUFB10	1.510035	1.153539	1.976704	0.002704	0.036745
TYMP	0.864789	0.786438	0.950947	0.002718	0.003902
PCSK9	1.154929	1.051117	1.268993	0.002723	0.000813
PLAAT2	0.536274	0.356788	0.806053	0.002727	0.04335
UBA52	1.454117	1.138286	1.85758	0.00273	0.013716
VRK2	0.757058	0.63106	0.908213	0.002731	0.027709
SPACA9	1.335102	1.105082	1.613	0.002738	0.020438
POC1A	1.351195	1.109651	1.645318	0.002741	0.000517
GMPR	1.09704	1.032518	1.165593	0.002747	0.00054
PSMD2	1.456225	1.138652	1.86237	0.002749	0.001034
EIF4G1	1.486034	1.146599	1.925953	0.002754	0.00227
AMPD3	0.806413	0.700443	0.928415	0.00276	0.001232
ELAC2	1.502818	1.150883	1.962373	0.002769	0.006473
FZD6	0.870942	0.795575	0.953448	0.002769	0.040181
DIPK1C	1.104673	1.034907	1.179143	0.002783	0.001848
CLCN7	1.260679	1.082931	1.467601	0.002813	0.000473
PNLIPRP3	1.134382	1.044287	1.232249	0.002824	0.010793
WWC1	1.185507	1.060218	1.325601	0.002826	0.039937
ARHGAP4	0.846162	0.758244	0.944274	0.002842	0.009633
DDX51	1.456448	1.137723	1.86446	0.002846	0.008258
BCAN	1.071039	1.023828	1.120428	0.002847	0.024893
MCAT	1.357998	1.110693	1.660367	0.002849	0.007833
RDH5	0.807346	0.701385	0.929315	0.002872	0.003717
PSTPIP2	0.835205	0.741928	0.940208	0.002879	0.000497
LCN12	0.528202	0.347057	0.803896	0.002895	0.01018
COX5A	1.45648	1.137136	1.865506	0.002905	0.01606
PLSCR4	0.848345	0.761299	0.945343	0.002906	0.037821
YIF1B	1.386485	1.118111	1.719276	0.00291	0.001698
GTF3C1	1.49152	1.146342	1.940636	0.002912	0.00523
TONSL	1.280662	1.088113	1.507285	0.002922	0.030748
IMPDH2	1.287259	1.089914	1.520336	0.00294	0.003496
NLRP3	0.77862	0.660222	0.918252	0.002946	0.001223
NTNG2	0.708944	0.565115	0.88938	0.002946	0.000327
AGPAT2	1.250296	1.079075	1.448686	0.002951	0.006002

SNRPB2	0.76664	0.643422	0.913455	0.002954	0.047013
SERP1	0.694916	0.546643	0.883406	0.002955	0.012537
ATP5MC3	1.415437	1.125623	1.77987	0.002956	0.042046
ETV2	1.341053	1.105113	1.627366	0.002956	4.07E-05
CCDC191	0.754541	0.626641	0.908546	0.002958	0.011194
RFLNB	1.183442	1.059027	1.322475	0.00296	0.000253
GOLGA7B	1.134278	1.04382	1.232575	0.002965	0.000866
PSMA2	0.679585	0.526671	0.876897	0.002978	0.035112
ALDH3A1	1.223175	1.070906	1.397095	0.002979	0.02091
SCARB1	1.173989	1.05606	1.305087	0.00298	0.008392
SMIM43	0.704466	0.55901	0.887769	0.002989	0.000126
HOXA3	0.855304	0.771438	0.948287	0.002994	0.005961
IQGAP3	1.227201	1.072029	1.404835	0.002994	0.016323
RBFA	1.428242	1.128671	1.807325	0.003	0.021918
LARP1	1.483134	1.142672	1.925038	0.003053	0.001138
MGRN1	1.466164	1.138129	1.888747	0.003064	0.005489
PLEKHG6	1.331746	1.101696	1.609835	0.003067	0.022309
MOSPD2	0.805969	0.69872	0.929681	0.003069	0.006733
CNTROB	1.487519	1.143622	1.93483	0.003072	0.001588
BPHL	1.353927	1.107717	1.654863	0.003087	0.002583
P2RX5	0.813474	0.709494	0.932692	0.003091	0.004096
GPR19	1.226637	1.07133	1.404459	0.003101	0.044762
FANCC	1.403699	1.121177	1.757414	0.003101	0.038187
WRAP53	1.49824	1.146079	1.958611	0.003103	0.007809
FDPS	1.375153	1.113272	1.698638	0.003122	0.001389
AHCY	1.310164	1.095234	1.567273	0.003127	0.039623
MRPL4	1.454199	1.134344	1.864243	0.003131	0.000416
CAP2	1.122413	1.039631	1.211786	0.003135	0.02723
FABP5	1.167125	1.053385	1.293147	0.003136	0.017725
CIAPIN1	1.439588	1.130443	1.833277	0.003137	0.004866
MAPKAPK3	1.364424	1.110146	1.676944	0.003147	0.004767
FAM107B	0.847879	0.759903	0.94604	0.003153	0.003602
STX11	0.846989	0.758553	0.945734	0.003161	0.0108
PDCD10	0.78164	0.663676	0.920571	0.003163	0.00348
SLC22A4	0.815631	0.712338	0.933902	0.00318	0.033248
LPAR6	0.848494	0.760741	0.946369	0.003182	0.00886
LRATD2	0.894644	0.830811	0.963381	0.003201	0.021198
CA11	0.817818	0.715442	0.934843	0.003205	0.014268
COQ4	1.450342	1.132617	1.857197	0.003208	0.004773
KLF9	0.829589	0.732642	0.939364	0.003214	0.002348
LETM1	1.394384	1.117689	1.739577	0.00322	0.002913
NLRP7	0.534452	0.352253	0.810892	0.003225	0.001747
BRAT1	1.397264	1.118261	1.745877	0.003245	0.007349
ZNF142	1.57151	1.162969	2.12357	0.003252	0.001554
BCS1L	1.476201	1.138709	1.913719	0.003274	0.0028
LARP7	0.806073	0.698178	0.930642	0.003278	0.037852
NPFFR1	0.575918	0.398662	0.831987	0.003282	0.000392
GSDMB	0.780803	0.662041	0.920869	0.00329	0.034189
C1R	0.874943	0.800352	0.956486	0.003298	0.048812
NPLOC4	1.426522	1.125545	1.807981	0.003302	0.011443
CD1B	0.73464	0.59804	0.902441	0.003304	0.000248

ZNF74	1.350821	1.105246	1.650961	0.003309	0.00456
GBX2	1.169622	1.053513	1.298527	0.003312	0.006691
C1S	0.886267	0.817649	0.960643	0.003319	0.040532
MYO16	1.299185	1.090931	1.547194	0.003321	0.02554
ZNFX1	0.742079	0.608054	0.905645	0.003334	0.039358
COL5A1	1.126611	1.040329	1.220048	0.003362	0.000218
MMP17	1.141814	1.044945	1.247664	0.003369	0.002376
GLIS1	1.265633	1.081036	1.481753	0.003404	0.000906
KCNJ10	0.90221	0.842164	0.966539	0.003406	0.000253
NDUFV1	1.410259	1.120239	1.775363	0.003427	0.016813
CHAC1	1.148732	1.04686	1.260517	0.003428	0.012594
HADHB	1.464838	1.134344	1.891624	0.003431	0.003246
OCSTAMP	0.421996	0.236712	0.75231	0.003447	0.004971
PTDSS1	1.418645	1.122185	1.793423	0.003458	0.01604
CCDC50	0.823212	0.72254	0.93791	0.003465	0.012953
CCR4	0.808812	0.701523	0.932511	0.003475	0.000727
TRUB2	1.421299	1.122738	1.799255	0.003475	0.027085
KLHL18	1.551346	1.155479	2.082837	0.003485	0.005569
ENPP7	0.048131	0.006284	0.368647	0.003493	0.006563
ABCG1	0.846123	0.756366	0.946531	0.003496	0.005716
EFNA3	1.183986	1.057083	1.326124	0.003504	0.014365
HTRA4	0.790534	0.675128	0.925666	0.003508	0.012307
CLDND1	0.750218	0.618572	0.909881	0.003508	0.044486
TESC	0.900522	0.839337	0.966166	0.003514	0.0456
CFP	0.811169	0.704816	0.93357	0.003516	0.007922
PRKCSH	1.459821	1.132277	1.882118	0.00352	0.00166
PWWP2B	1.265994	1.080493	1.483342	0.003527	0.000338
CYBC1	0.690599	0.538493	0.88567	0.00354	0.041402
RHOU	0.840259	0.747508	0.94452	0.003541	0.00541
COL25A1	1.160488	1.050014	1.282585	0.003544	0.005461
POLD2	1.378323	1.110943	1.710056	0.003544	0.0216
ASGR2	0.704862	0.557196	0.891662	0.003546	0.006081
ADORA1	0.844007	0.753057	0.94594	0.003553	0.036961
MDC1	1.362283	1.106528	1.677153	0.003567	0.000326
USP36	1.417246	1.120919	1.79191	0.003571	0.014791
TCL1A	0.871262	0.794123	0.955893	0.003572	0.00862
PCNX2	0.806537	0.697931	0.932045	0.003572	0.003353
CAMKK1	1.329254	1.097605	1.609793	0.003577	0.023632
LINC0155(	0.758703	0.630057	0.913616	0.00358	0.03354
SMAD7	0.781458	0.661911	0.922596	0.003603	0.044366
CD209	0.853562	0.767236	0.949601	0.003608	0.002953
RTN4R	1.139363	1.043496	1.244038	0.003621	0.00289
ERP29	1.475736	1.135385	1.918113	0.003624	0.001138
CRNN	1.1496	1.046522	1.26283	0.00363	0.009548
ZCCHC24	0.825128	0.724817	0.939321	0.003655	0.01075
SMARCD1	1.476457	1.135267	1.920186	0.003658	6.13E-05
AP2A1	1.510852	1.143754	1.995774	0.003664	0.00062
TMEM63B	1.358967	1.104924	1.67142	0.003673	0.045793
RABGAP1L	0.712431	0.566672	0.895682	0.003693	0.021738
PWP1	1.486437	1.137433	1.942527	0.003695	0.003488
NRM	1.300421	1.089058	1.552805	0.0037	0.002386

CLMN	0.822441	0.720668	0.938586	0.003727	0.007528
ANXA7	0.721732	0.578981	0.899678	0.00373	0.002085
PEMT	1.31484	1.092767	1.582042	0.003734	0.002732
MYO1D	1.163416	1.050263	1.28876	0.003739	0.025136
SCARF1	0.782476	0.662881	0.923647	0.00375	0.018725
SLC7A8	1.141969	1.0439	1.249251	0.003758	0.000323
GIT1	1.361832	1.105046	1.678288	0.003768	0.000835
RAPGEF6	0.699816	0.549665	0.890983	0.003771	0.03654
LIPM	0.750151	0.617538	0.911241	0.003774	1.74E-05
SPOUT1	1.517245	1.144235	2.011853	0.003781	0.007019
TEDC2	1.262547	1.078246	1.47835	0.003783	0.044531
RTP4	0.865163	0.784345	0.954309	0.003796	0.020071
SARS2	1.44736	1.126722	1.859243	0.003807	0.029875
TMEM184B	1.327046	1.09555	1.607459	0.003816	0.004386
CERK	1.279317	1.082638	1.511726	0.003825	0.033025
FAM83G	1.181914	1.055352	1.323654	0.003825	0.033831
HLA-A	0.854251	0.76775	0.950497	0.003827	0.000776
MYBL2	1.187477	1.056938	1.334137	0.003828	0.007234
DGKA	0.804511	0.694207	0.932342	0.003839	0.003883
ATG16L2	0.775585	0.652772	0.921503	0.00386	0.039244
ISG20L2	1.362403	1.104541	1.680465	0.003868	0.004011
ABCC1	1.322166	1.093888	1.598081	0.003878	0.03824
RASGEF1B	0.781589	0.661213	0.923881	0.00388	0.03095
KCTD18	0.748314	0.614611	0.911102	0.003889	0.043903
SPRING1	1.319986	1.093063	1.594019	0.00392	0.002358
GTSF1	0.8876	0.818521	0.962508	0.003922	0.001248
BBS2	0.76999	0.644657	0.91969	0.003932	0.018766
IRF5	0.826398	0.725902	0.940807	0.003948	0.031063
TREML1	0.703547	0.553924	0.893586	0.003949	0.032256
NCR1	0.738259	0.600488	0.907639	0.003983	0.001186
PTPRO	0.78185	0.661254	0.92444	0.003987	2.61E-06
SPIB	0.848171	0.758203	0.948815	0.003998	0.019348
SNAPC4	1.376241	1.107214	1.710634	0.004006	0.024397
CD40LG	0.819657	0.715825	0.938549	0.004006	0.000736
SLC22A17	0.896793	0.832665	0.965861	0.004008	0.000126
RBPMS2	1.124122	1.038014	1.217373	0.004008	0.019867
INTS1	1.344112	1.098886	1.644061	0.004009	0.002299
CHRDL1	0.908219	0.850567	0.96978	0.004014	0.003918
TLR6	0.828771	0.729225	0.941907	0.004019	0.02186
CDC42BPB	1.362509	1.103569	1.682206	0.004023	0.023642
CCDC74A	1.178263	1.053634	1.317633	0.004029	0.03652
SLC26A4	0.866434	0.785705	0.955458	0.004065	0.001234
SENP7	0.790998	0.674072	0.928207	0.004068	0.036498
MMP24OS	1.339293	1.097272	1.634695	0.004069	0.000939
FAM166B	0.615082	0.441436	0.857036	0.004085	0.012911
MRTFA	1.445609	1.124071	1.859121	0.00409	0.015466
SPIC	0.647092	0.480659	0.871154	0.004115	0.004472
PSME3	1.513696	1.140384	2.009215	0.004116	0.006113
CCL3L1	0.834089	0.736807	0.944215	0.004142	0.00216
TENM2	1.215721	1.063703	1.389466	0.004156	0.000527
TNFRSF13I	0.77695	0.653776	0.92333	0.004161	0.000141

PPP4R4	0.619591	0.446593	0.859604	0.004162	0.002265
OBSCN	1.226762	1.06666	1.410895	0.004178	0.021002
ABCB6	1.32402	1.092579	1.604487	0.004194	0.045332
UBQLN4	1.348877	1.09894	1.655657	0.004206	0.002319
TMEM52B	0.650166	0.484149	0.87311	0.00421	2.40E-06
AHR	0.870355	0.791363	0.957231	0.004231	0.042497
ETAA1	0.796833	0.681947	0.931074	0.00425	0.015643
LRRCC1	0.849484	0.759556	0.950058	0.004272	0.022017
NAIP	0.48462	0.294836	0.796566	0.004276	0.008513
RPL3L	1.536665	1.14437	2.063442	0.004281	0.025967
RNF175	0.888471	0.819225	0.963571	0.004286	0.014141
CAMK4	0.827905	0.727231	0.942516	0.004305	0.00337
BTBD6	1.346803	1.097792	1.652297	0.004311	0.00249
ZSCAN22	1.527433	1.1419	2.04313	0.004317	0.000452
MRPS16	1.516154	1.1392	2.01784	0.004323	0.037101
SPPL3	1.573317	1.152311	2.148142	0.004342	0.001088
ADAM15	1.303968	1.086533	1.564916	0.004349	0.009634
TRIM14	0.819463	0.714628	0.939677	0.004361	0.000232
ZNF726	1.392205	1.108887	1.74791	0.004368	0.005183
TSPAN33	0.849146	0.758775	0.95028	0.004396	0.035627
BLOC1S5-T	0.603375	0.426166	0.854273	0.004403	0.002627
CSF2RA	0.875518	0.798946	0.959429	0.004415	0.001484
SLC29A3	0.796148	0.680506	0.931441	0.004415	0.003423
IGSF9	1.183855	1.053943	1.32978	0.004429	0.027443
PAAF1	1.366876	1.10217	1.695156	0.00443	0.049827
CTSC	0.811543	0.702788	0.937127	0.004448	0.004503
UBE2C	1.217849	1.063201	1.39499	0.004449	0.016484
UBQLNL	0.646571	0.47867	0.873365	0.004475	4.14E-05
ITGAX	0.844426	0.751463	0.94889	0.004489	0.007953
MKI67	1.241213	1.069324	1.440734	0.004494	0.000903
ZNF114	1.199629	1.058035	1.360172	0.004507	0.007025
CYTH3	1.197432	1.057418	1.355985	0.004512	0.007925
LRCH2	0.851374	0.761886	0.951373	0.004515	0.001215
NMRK2	1.086293	1.025938	1.150199	0.00454	0.039727
MCRS1	1.551365	1.14541	2.101199	0.004553	0.003194
ACTR3	0.745121	0.607955	0.913234	0.004592	0.033764
QARS1	1.449758	1.121377	1.874302	0.004595	0.017436
ACHE	0.826589	0.724578	0.942962	0.004599	0.004941
TMEM158	0.900758	0.83793	0.968297	0.004607	0.000403
BBC3	1.252748	1.071817	1.464222	0.004634	0.00438
ACOD1	0.592039	0.411799	0.851169	0.004656	3.01E-06
SLC40A1	0.89106	0.822642	0.965167	0.004658	0.004711
KALRN	0.796749	0.680677	0.932613	0.004678	0.00937
MRAS	0.82442	0.721166	0.942457	0.004684	0.001201
DAPK3	1.363059	1.099577	1.689677	0.004712	0.011539
ITIH6	0.921002	0.869804	0.975215	0.004802	0.003822
ANO9	0.829398	0.728278	0.944559	0.004806	0.025949
GET3	1.521607	1.13644	2.037316	0.00482	0.01925
CDS2	1.369731	1.100584	1.704697	0.004823	0.034863
RP11-108K	0.052414	0.006742	0.40745	0.004832	0.028615
CDK15	1.193625	1.055351	1.350016	0.004839	0.004299

TFAP4	1.405175	1.108999	1.780451	0.004853	0.000331
RAB3D	1.219229	1.062115	1.399583	0.004861	0.006824
CEP290	0.813386	0.704401	0.939232	0.004891	0.04698
RASGRP2	0.838267	0.741328	0.947882	0.004899	0.010399
NAB2	1.357329	1.09709	1.679299	0.004905	0.020315
BRI3	1.37294	1.100831	1.71231	0.004918	0.007584
NPM3	1.302045	1.083271	1.565002	0.00492	0.006282
HERPUD1	0.742509	0.603372	0.913732	0.004922	0.01406
N4BP3	1.18802	1.053579	1.339618	0.004927	0.000745
NEXN	0.810022	0.699265	0.938321	0.004975	0.000136
CFI	0.900236	0.836555	0.968765	0.004989	0.027386
TDRP	1.157792	1.045209	1.282502	0.004999	0.028628
TRIM11	1.526367	1.136118	2.050664	0.005	0.01816
IL12B	0.421026	0.230126	0.770286	0.005004	0.029692
KIF20A	1.225612	1.063297	1.412704	0.005005	0.024948
PRMT7	1.352053	1.095235	1.669091	0.005011	0.031718
NUP93	1.311374	1.085164	1.58474	0.005016	0.003226
EXO1	1.214024	1.060163	1.390215	0.005033	8.17E-05
BCL2L12	1.397587	1.10577	1.766416	0.005089	9.21E-05
ZNF101	0.752046	0.616086	0.91801	0.005098	0.019414
AKNA	0.838317	0.740979	0.94844	0.005101	0.024231
SCT	0.782807	0.659504	0.929163	0.005108	0.031061
EI24	1.363599	1.097514	1.694194	0.005109	0.026036
ECE2	1.331506	1.089698	1.626972	0.005109	0.039956
FLNC	1.117748	1.033961	1.208324	0.00511	0.008018
UBALD1	1.373009	1.099734	1.71419	0.005118	0.014059
STRA6	0.897656	0.832303	0.96814	0.005118	0.024171
BCAR1	1.257669	1.071167	1.476644	0.005119	0.010633
CAMK1	0.80913	0.697596	0.938497	0.00513	0.020988
WDR81	1.298183	1.081347	1.5585	0.005131	0.010098
TACO1	1.445293	1.116686	1.870598	0.005133	0.00627
PRR5L	0.782914	0.659549	0.929353	0.005151	0.00456
EFCAB13	0.748115	0.610472	0.916794	0.005152	0.001035
IZUMO4	0.716201	0.566838	0.90492	0.005155	0.001237
TOM1	1.33968	1.091454	1.64436	0.005159	0.018118
SSTR3	0.632275	0.458532	0.871852	0.005166	0.000101
TIGD4	0.744126	0.604778	0.915582	0.005212	0.014213
AP2S1	1.403179	1.106308	1.779714	0.005223	0.027697
DSE	0.817779	0.710093	0.941795	0.005232	0.001394
EP400	1.478189	1.12353	1.944803	0.005237	0.007193
PLEKHG4B	1.22478	1.062222	1.412214	0.005258	0.005171
PRNP	0.849133	0.756993	0.952488	0.005261	0.018771
PSMB7	1.395322	1.104208	1.763186	0.005266	0.001658
AARD	0.872608	0.792882	0.96035	0.00531	0.002484
LAMC3	1.137404	1.038951	1.245186	0.005317	0.015069
PLCXD2	0.568662	0.382311	0.845847	0.00533	0.002749
H2AZ1	1.272892	1.074174	1.508372	0.005333	0.003217
SNTA1	1.204855	1.056748	1.37372	0.005357	0.001812
MYO1A	0.320193	0.143621	0.713847	0.005369	0.025138
ELL2	0.818912	0.711422	0.942642	0.00539	0.015547
SLC25A13	1.252083	1.068685	1.466954	0.005402	8.60E-06

GZMM	0.886264	0.813991	0.964954	0.005404	0.04013
RBP5	0.876772	0.799137	0.961948	0.005435	0.002746
POLR2E	1.44955	1.115692	1.88331	0.005442	0.00122
GRAMD1B	0.833858	0.733584	0.947839	0.005445	0.003002
SERPINI1	0.872388	0.792281	0.960595	0.005469	0.034953
OLR1	0.878132	0.801195	0.962456	0.00547	0.000531
TNFAIP1	1.485836	1.123636	1.96479	0.005475	0.010345
BHLHA15	0.836387	0.737311	0.948777	0.00548	0.000248
KIAA0100	1.384037	1.100348	1.740866	0.005485	0.000345
RRAS	0.818372	0.710408	0.942745	0.00549	0.000901
TMEM39A	0.733432	0.589226	0.912931	0.005512	0.022432
SURF2	1.373338	1.097642	1.718281	0.005523	0.040126
ACRV1	1.489571	1.124035	1.97398	0.005539	0.002061
DDX54	1.452314	1.11544	1.890929	0.005583	0.000683
C15orf48	0.87959	0.803286	0.963143	0.005588	0.003709
CIT	1.238903	1.064699	1.441609	0.005591	0.015818
ASMT	0.469169	0.274687	0.801346	0.005592	0.019329
FCRL4	0.542742	0.35225	0.83625	0.005593	0.000529
FAM241A	0.776085	0.648679	0.928515	0.005595	0.006885
BTBD9	1.317504	1.083892	1.601468	0.005624	0.00789
SOX12	1.279867	1.074717	1.524178	0.005633	0.006087
NLRP1	0.828181	0.724665	0.946485	0.005652	0.000234
FDCSP	0.940468	0.90046	0.982254	0.005654	0.00148
YIPF6	0.753299	0.616345	0.920683	0.005654	0.023179
SNRPC	1.396862	1.102408	1.769967	0.005655	0.042845
BANP	1.472751	1.119487	1.937491	0.005665	0.015162
RNF10	1.639283	1.154949	2.326726	0.005671	0.005164
LINGO3	0.651155	0.48025	0.882878	0.005746	0.000473
KIR2DL1	0.410157	0.217907	0.77202	0.005749	0.00015
LAGE3	1.342131	1.089123	1.653914	0.005762	0.000973
NOP16	1.361777	1.093674	1.695603	0.005773	0.017524
TUBE1	0.745302	0.604872	0.918335	0.005784	0.030161
FH	1.346951	1.089892	1.66464	0.00584	0.000586
NCEH1	0.841949	0.744993	0.951524	0.005851	0.000452
RASSF10	1.219468	1.058922	1.404355	0.005872	0.018673
SPAG5	1.28472	1.074964	1.535405	0.005873	0.007159
MFF	0.713513	0.561072	0.907373	0.005912	0.017229
B3GLCT	1.279259	1.07342	1.524569	0.005932	0.002983
CCT3	1.374378	1.095732	1.723884	0.005945	0.017212
QPCTL	1.260384	1.068727	1.486411	0.005964	0.004477
PRRC2A	1.383176	1.097641	1.742988	0.005965	0.000259
GAPT	0.817098	0.707498	0.943675	0.005979	0.000712
C17orf67	1.285852	1.074806	1.538338	0.005985	0.021082
NEURL1B	1.205378	1.054995	1.377196	0.006007	0.000925
CCL23	0.799913	0.68211	0.938059	0.006021	0.005545
FRMPD3	0.637447	0.462248	0.879049	0.006029	0.028102
MIXL1	0.788242	0.665123	0.934151	0.006031	0.008764
KLF5	1.12864	1.035247	1.230458	0.006032	0.018355
ARSJ	0.844769	0.748933	0.952869	0.006037	0.001337
CASKIN2	1.372292	1.094695	1.720284	0.006059	0.016191
VARS1	1.329576	1.084834	1.629531	0.006061	0.000589

FAM120C	0.77667	0.648408	0.930303	0.006061	0.014208
CLSPN	1.213307	1.056823	1.392962	0.006062	0.003799
SEC16A	1.440592	1.109968	1.869697	0.006065	0.00407
CFAP251	1.292926	1.076173	1.553335	0.006068	8.14E-05
RIPK3	0.824846	0.718801	0.946537	0.006097	0.001691
NPHP4	1.262419	1.068723	1.491221	0.006106	0.038748
GIPR	0.719588	0.568696	0.910516	0.006131	0.009634
TYMS	1.217174	1.057574	1.40086	0.006134	0.045777
DCSTAMP	0.880087	0.803222	0.964307	0.006155	0.001971
NYAP2	0.113414	0.023882	0.538603	0.006174	0.020088
TUBB6	1.237421	1.062418	1.441249	0.006177	0.017775
KIF18B	1.238486	1.062677	1.44338	0.006177	0.006489
HYAL4	1.576925	1.1381	2.184951	0.006192	0.024606
PPM1N	0.799559	0.681226	0.938447	0.006193	0.016271
ZBTB45	1.432513	1.107449	1.852992	0.006197	0.007023
RPS6KB2	1.438785	1.108804	1.86697	0.0062	0.046672
STUM	1.149213	1.040269	1.269565	0.006203	0.004601
SLC35G6	3.326822	1.406308	7.870068	0.006217	0.01404
TMEM201	1.322519	1.082503	1.615752	0.006222	0.002526
STK35	1.393141	1.09851	1.766795	0.006239	0.020245
ZDHHC3	1.588846	1.14017	2.214084	0.006242	0.010586
FMO3	0.803803	0.68729	0.940068	0.006267	0.014098
COL1A1	1.107831	1.02939	1.192249	0.006275	0.000655
TERF2IP	0.718622	0.566971	0.910836	0.006291	0.010873
PRRG4	0.85654	0.766469	0.957195	0.006301	0.020347
THAP8	1.413352	1.102607	1.811673	0.006314	0.0047
NDST1	1.314445	1.08015	1.599561	0.006339	0.01916
RBMXL1	0.764929	0.631009	0.927271	0.006354	0.038477
VCPIP1	0.763335	0.628749	0.926731	0.006356	0.022285
CDC42EP1	1.150064	1.040171	1.271568	0.006361	0.042634
TXK	0.793794	0.672452	0.937031	0.006365	0.002084
VNN1	0.797222	0.677441	0.938182	0.006369	0.032968
NR4A2	0.862336	0.775291	0.959153	0.006369	0.003371
IL7	0.800241	0.681828	0.939219	0.006383	0.013498
LBH	0.855646	0.764951	0.957094	0.00639	0.038466
HPS4	1.217698	1.056963	1.402877	0.006392	0.001161
MRPS25	1.298376	1.076175	1.566457	0.0064	0.000408
HTR4	0.00208	2.45E-05	0.176332	0.006409	2.23E-05
PRDM8	0.801867	0.684094	0.939915	0.006439	0.000934
GATA3	0.859421	0.770664	0.958401	0.006451	0.025766
ERCC2	1.376751	1.093779	1.732931	0.006459	0.028158
IFNGR1	0.803861	0.686946	0.940674	0.006476	0.011713
C3	0.920013	0.866422	0.976917	0.006476	0.006869
POF1B	1.171384	1.045277	1.312706	0.00649	0.041409
SPAG4	0.855974	0.765281	0.957416	0.006498	0.026562
TMEM241	1.351711	1.087958	1.679406	0.006506	0.000396
ABHD18	0.748215	0.607139	0.922072	0.006508	0.044733
CRTAP	1.341837	1.085627	1.658511	0.006529	0.02625
TPX2	1.245588	1.063226	1.459229	0.006547	0.00016
ITGAD	0.767708	0.6345	0.928882	0.006554	5.02E-05
STK17B	0.864163	0.777814	0.960098	0.006566	0.006331

AC092835-	1.344146	1.085929	1.663763	0.00658	0.035859
LMNB2	1.289457	1.073422	1.54897	0.006582	0.00059
FAM222B	1.313272	1.078872	1.598598	0.006592	0.000814
BPI	0.643414	0.468059	0.884465	0.006604	0.005082
C7orf26	1.441621	1.10707	1.877272	0.006628	0.027919
FABP3	1.159179	1.041927	1.289626	0.006631	0.011391
SLC25A23	1.263833	1.06727	1.496597	0.006632	0.038013
FBXL5	0.75935	0.622425	0.926398	0.006657	0.044498
PARP4	0.812601	0.699482	0.944014	0.006662	0.008964
INTS4	1.31963	1.079998	1.612433	0.006674	0.02861
DPYS	0.656265	0.484038	0.889774	0.006689	0.000685
TSPAN10	1.085023	1.022873	1.15095	0.0067	0.042961
NR1D2	0.834257	0.731827	0.951024	0.006702	0.014046
PSMB5	1.439411	1.106186	1.873016	0.006705	0.001904
KLHL2	0.78486	0.658769	0.935085	0.006706	0.049125
UQCRQ	1.370963	1.091304	1.722287	0.006716	0.019478
DOCK6	1.301063	1.075598	1.573791	0.006718	0.01634
ABHD14B	1.277827	1.070234	1.525686	0.00672	0.002118
C1orf122	1.3627	1.089357	1.70463	0.006744	0.02241
LHX6	1.202126	1.05223	1.373375	0.006744	0.000913
TMEM132D	1.156563	1.041039	1.284907	0.006748	0.048772
ITGB3	0.920391	0.866774	0.977325	0.00675	0.01039
ICAM2	0.820757	0.711399	0.946925	0.00678	0.015666
PADI1	1.315839	1.078693	1.60512	0.006788	0.002361
URB2	1.295701	1.074047	1.563099	0.006806	0.022479
MYBBP1A	1.345151	1.085196	1.667378	0.006806	0.028955
IGF2	1.106132	1.028162	1.190014	0.006838	0.010526
MLST8	1.37961	1.092625	1.741972	0.006842	0.003182
BANK1	0.843437	0.745504	0.954235	0.006854	3.03E-06
CLPTM1	1.319021	1.078957	1.612499	0.006905	0.007156
LILRA2	0.762752	0.626678	0.928374	0.006908	0.000376
DCTN6	0.746632	0.603945	0.92303	0.006931	0.038162
TK1	1.222254	1.056553	1.413942	0.006933	0.013868
NDUFAF8	1.306686	1.076059	1.586743	0.006937	0.005224
SLC45A2	1.076218	1.02033	1.135167	0.00694	0.004621
SEPHS1	1.423069	1.101472	1.838564	0.006946	0.044459
CAD	1.312354	1.07722	1.598813	0.006968	0.000731
IL17D	1.129244	1.033798	1.233501	0.006982	0.028265
RBM15B	1.53883	1.124886	2.105101	0.007016	0.014142
CCL19	0.93482	0.890106	0.98178	0.007033	0.038479
XPO5	1.283849	1.070446	1.539797	0.007062	0.008006
DERA	1.281032	1.069802	1.533968	0.007063	0.008704
ALG9	1.419051	1.099918	1.830778	0.007088	0.03631
HERC6	0.857131	0.766123	0.95895	0.007105	0.002613
ACSL6	0.595363	0.408014	0.868736	0.007149	0.001795
MRPL14	1.418164	1.099367	1.829407	0.007163	0.042709
IGHMBP2	1.24128	1.060316	1.453129	0.007179	0.015327
NCBP2AS2	1.364438	1.087814	1.711404	0.007186	0.003817
SCNN1G	1.374906	1.090061	1.734183	0.007188	0.000194
CLCF1	0.849287	0.753908	0.956734	0.007195	0.036392
EBP	1.294927	1.072452	1.563553	0.007205	0.022343

SLC4A2	1.288881	1.071037	1.551033	0.007221	0.013322
IFNGR2	0.7636	0.627168	0.929711	0.007238	0.038959
LONP1	1.336482	1.081434	1.65168	0.007262	0.011472
H2AJ	1.145592	1.037308	1.26518	0.007297	0.027181
RNF144A	1.214014	1.053573	1.398889	0.007328	0.003927
EHMT2	1.3506	1.08408	1.682644	0.007367	0.000462
NDUFA10	1.521265	1.119168	2.067829	0.007388	0.00352
NFATC2	0.887036	0.812549	0.968351	0.007393	0.038824
EHBP1L1	0.761976	0.624497	0.929721	0.007411	0.004351
IRX1	1.118466	1.030466	1.213981	0.007412	0.00035
THRAP3	1.516941	1.118153	2.057958	0.007416	0.001422
KCNK13	0.785889	0.658809	0.937483	0.007421	0.000412
LPAR5	0.839199	0.738108	0.954135	0.007431	0.00087
RNF187	1.363837	1.086601	1.711807	0.007444	0.024357
NCMAP	0.750294	0.607927	0.926002	0.007449	0.000436
GPRIN3	0.870961	0.787144	0.963704	0.007449	0.031881
SIX6	1.333569	1.080061	1.646579	0.007453	0.000501
ATL1	0.836819	0.734417	0.953499	0.007474	0.007782
PELP1	1.435379	1.101426	1.870587	0.007474	0.005599
ZNF850	1.284859	1.06929	1.543887	0.007474	0.011956
LBX1	1.247287	1.060841	1.466502	0.007475	0.021577
ITGA10	0.927426	0.877609	0.98007	0.007482	0.019833
TBCEL	0.792874	0.668864	0.939876	0.007484	0.009973
PGP	1.339332	1.081156	1.659159	0.007492	0.007141
GYPC	1.193985	1.048463	1.359705	0.007504	0.032597
ACTG1	1.385999	1.09094	1.760861	0.007526	0.004224
GNPDA1	1.37937	1.089457	1.746432	0.007547	0.004826
VARS2	1.338427	1.080739	1.657556	0.007549	0.014007
CD5L	0.876009	0.794908	0.965386	0.00757	0.008365
KCNMA1	0.838518	0.736765	0.954325	0.007625	0.000777
FCRL1	0.824485	0.715484	0.950091	0.007639	5.59E-05
CHTF8	1.443696	1.10211	1.891153	0.007681	0.030996
RPGRIP1	0.54238	0.345855	0.850576	0.0077	0.010182
CLDN11	0.89809	0.829826	0.97197	0.007703	0.013856
SPATA33	1.414645	1.096067	1.825819	0.007708	0.013756
CASKIN1	1.183911	1.045624	1.340486	0.007723	0.011162
UPK3A	0.610007	0.423982	0.877652	0.007742	0.003596
CD160	0.661709	0.48827	0.896754	0.007753	0.048702
MAP7D2	1.120083	1.03031	1.217678	0.007803	0.016261
PPP1R32	0.762068	0.623816	0.930961	0.007805	0.018789
HDGF	1.355121	1.083213	1.695282	0.007826	0.022107
CAPN1	1.365289	1.08532	1.717478	0.007832	0.009326
COMTD1	1.212497	1.051943	1.397556	0.007844	0.000564
CDCA4	1.296127	1.070521	1.56928	0.007852	0.003122
F12	1.13424	1.033601	1.244677	0.007881	0.011567
PARP1	1.337186	1.079061	1.657057	0.007924	0.003704
ZNF37A	0.79833	0.676035	0.942749	0.007933	0.036147
PHOSPHO1	0.650877	0.474028	0.893706	0.007939	0.01376
MRM3	1.4251	1.097106	1.851152	0.007945	0.001317
PRPF4	1.391073	1.090152	1.775057	0.007954	0.009303
GPRC5A	1.101427	1.025576	1.182888	0.007962	0.043435

RANGAP1	1.311957	1.073519	1.603356	0.007974	0.025555
SLC39A1	1.418595	1.095656	1.836719	0.007975	0.00468
PIGK	0.787049	0.659388	0.939426	0.008002	0.036012
PMCH	0.586917	0.395776	0.87037	0.008036	0.00032
KCNH1	1.176638	1.043268	1.327058	0.008048	0.001453
SMPD2	1.233838	1.056234	1.441307	0.008052	0.033516
DNAJC11	1.393714	1.090167	1.78178	0.008078	0.006614
PRICKLE3	1.368597	1.084905	1.726471	0.008108	0.037154
CD200	0.888046	0.813314	0.969646	0.008116	0.015373
RHEX	0.701571	0.539492	0.912342	0.008183	0.000123
CALM3	1.456308	1.10192	1.92467	0.008239	0.004143
CMTM8	0.878057	0.797308	0.966984	0.00824	0.002665
WDR5B	0.758554	0.617951	0.931149	0.008242	0.008245
CA14	1.078681	1.019694	1.141081	0.008299	0.003981
TDRD6	0.688147	0.521379	0.908259	0.008302	0.021853
RGS9	0.771651	0.636486	0.93552	0.008331	0.000365
DENND2D	0.89117	0.818047	0.970829	0.008347	0.014639
UBAC1	1.477223	1.105341	1.974221	0.008368	0.002616
CHST10	1.321381	1.074113	1.625573	0.008381	0.049212
CYB5R3	1.263174	1.061734	1.502833	0.008394	0.015523
UCK2	1.282344	1.065727	1.542991	0.008432	0.006778
SH3GL1	1.351818	1.0802	1.691733	0.008437	0.002203
CEMIP	0.899807	0.831817	0.973356	0.008447	0.027716
ZNF526	1.434971	1.096552	1.877834	0.008499	0.001436
FOXA3	0.809755	0.691962	0.947599	0.008512	0.000447
SERPINA3	0.836611	0.732459	0.955573	0.008541	0.004846
DACT3	1.147726	1.035703	1.271866	0.008553	0.041758
VWA5A	0.847768	0.749566	0.958835	0.008559	0.008343
SMPDL3B	0.851021	0.75459	0.959776	0.008562	0.013332
CSNK2B	1.402378	1.089775	1.804651	0.008587	0.022824
CD81	1.308379	1.070704	1.598812	0.008592	0.025108
HS1BP3	1.370803	1.083293	1.73462	0.008636	0.019119
MYO1C	1.346081	1.078231	1.680469	0.008657	0.044868
FNDC10	1.112892	1.027468	1.205419	0.008666	0.01106
PQBP1	1.449389	1.098578	1.912224	0.008667	0.00642
MTX3	0.816873	0.70231	0.950124	0.008701	0.014543
POMT2	1.327465	1.074272	1.640333	0.008704	0.009491
ADRB1	0.816011	0.701	0.949891	0.008711	2.27E-06
CDT1	1.211288	1.049601	1.397882	0.008736	0.03177
EFHD1	1.088777	1.021705	1.160251	0.008745	0.011106
TULP1	1.363799	1.081251	1.720181	0.008807	0.001895
KIR2DL3	0.349603	0.159232	0.767571	0.008813	0.000153
MAN1C1	0.840125	0.737424	0.95713	0.008829	0.009546
SYNE1	0.765833	0.627188	0.935126	0.008839	0.048774
ANKRD55	0.677356	0.505924	0.906878	0.008884	0.001349
CPA5	0.644496	0.463743	0.895702	0.0089	0.013493
SPRY2	0.863214	0.773088	0.963847	0.008937	0.023166
LTK	1.113325	1.027234	1.20663	0.008941	0.004658
TSKU	1.13908	1.033128	1.255897	0.008942	0.018181
PPIA	1.490202	1.104945	2.009787	0.008952	0.0497
TMED8	0.765647	0.626692	0.935412	0.008964	0.049468

E2F1	1.233887	1.053896	1.444616	0.008989	0.004139
RP3-370M2	0.714713	0.555476	0.919598	0.009009	0.000383
POC5	0.748048	0.601588	0.930164	0.009024	0.029302
MSANTD3	1.267453	1.060883	1.514246	0.009026	0.022156
GLMP	1.197995	1.046056	1.372003	0.009036	0.004039
TCF19	1.282764	1.063856	1.546717	0.009098	0.000104
HDAC8	0.659966	0.482898	0.901959	0.009124	0.016319
CARNS1	0.730906	0.577443	0.925156	0.009136	0.012998
CAPS	0.867162	0.779006	0.965294	0.009168	0.002057
GFUS	1.326691	1.072559	1.641036	0.009171	0.002473
UBE2I	1.589914	1.121706	2.253555	0.00918	0.013435
GTSE1	1.198496	1.045837	1.37344	0.009197	0.00809
KCP	1.15916	1.037119	1.295562	0.009266	0.010643
TMEM220	1.214235	1.049028	1.40546	0.009284	0.005928
ATAD3A	1.257359	1.05806	1.494197	0.009297	0.012851
C1orf54	0.796831	0.671489	0.94557	0.009298	0.00231
CPXM1	1.098566	1.023417	1.179233	0.009317	0.007493
ATP5MJ	1.380039	1.082529	1.759312	0.00932	0.013832
MMS19	1.387556	1.083878	1.776316	0.009347	0.009667
NAV1	1.234137	1.05309	1.44631	0.009348	0.024704
PPP2R5D	1.365864	1.079507	1.728182	0.009397	0.011772
FCGR2B	0.850611	0.752842	0.961077	0.009398	0.004757
ADGRF3	0.439157	0.235956	0.817353	0.009424	0.020457
MAZ	1.343753	1.07506	1.679601	0.009436	3.44E-05
RASSF7	0.804302	0.682271	0.948159	0.009486	0.032675
MMP2	1.092656	1.021888	1.168324	0.009494	0.000686
FBXW9	1.288087	1.06374	1.55975	0.009519	0.00398
CDYL	1.268915	1.059829	1.51925	0.009528	0.012443
DECR2	1.300093	1.065929	1.585698	0.009595	3.21E-05
ZSWIM1	1.44633	1.093828	1.912431	0.00962	0.042522
LILRB5	0.844513	0.743102	0.959765	0.009621	0.015663
ART5	1.232878	1.052113	1.444701	0.009655	0.016606
UTS2	0.774876	0.638708	0.940075	0.009691	0.004545
HGSNAT	1.315453	1.068675	1.619216	0.009694	0.038552
TVP23B	0.748918	0.601553	0.932383	0.009704	0.013032
SYNGR3	0.831664	0.723145	0.956469	0.00977	0.015158
TSR3	1.451979	1.094207	1.926732	0.009774	0.011541
PEX26	1.391429	1.082902	1.787858	0.009804	0.031589
PPARD	1.283845	1.062029	1.551989	0.009828	0.014117
GRB2	0.678739	0.505755	0.91089	0.009829	0.0282
SIRT2	1.40995	1.086202	1.830194	0.009845	0.006902
CHERP	1.378322	1.080278	1.758594	0.009848	0.001015
MGST3	1.308957	1.066838	1.606026	0.009881	0.036011
ATP13A5	1.569746	1.114004	2.211934	0.009967	0.017312
PIP4K2C	1.267606	1.058417	1.51814	0.009968	0.002232
CNNM1	1.143275	1.032561	1.265861	0.009979	0.01044
SERPINA12	1.171442	1.038567	1.321318	0.009995	0.013012
PPFIA3	1.238166	1.052354	1.456785	0.010022	0.004991
ST6GALNAC	0.873264	0.787662	0.968169	0.010038	0.010557
PLPPR1	0.803201	0.679763	0.949054	0.010048	0.026665
NCALD	1.136486	1.030955	1.252819	0.01008	0.039154

FAM83D	1.188248	1.041835	1.355238	0.010146	0.012813
CDCA5	1.222575	1.048904	1.425001	0.010148	0.01976
CYB5R1	1.307333	1.065723	1.603718	0.010155	0.001761
FHL3	1.278205	1.059946	1.541405	0.010188	0.03582
VPS18	1.303418	1.064798	1.595514	0.010213	0.02906
ANKRD24	0.721803	0.5628	0.925728	0.010233	0.001159
ACACA	1.31292	1.066435	1.616374	0.01028	0.015636
DVL3	1.341668	1.071905	1.679323	0.010281	0.008928
SLC2A1	1.173373	1.038487	1.325778	0.010285	0.002859
S100B	0.926316	0.873698	0.982103	0.010311	0.002699
SEPTIN5	1.153758	1.034224	1.287107	0.010377	0.032816
DNAJA4	1.102832	1.023242	1.188611	0.010432	0.018955
PRKCG	0.273243	0.101189	0.73785	0.010473	0.008722
SLC43A3	1.201131	1.043841	1.382122	0.010494	0.003262
RHPN2	0.842783	0.739285	0.96077	0.010509	0.025319
FBXW8	1.3279	1.068421	1.650396	0.010571	0.000897
ABCC3	0.826884	0.714717	0.956654	0.010596	0.020495
BCORL1	1.29185	1.061488	1.572205	0.010605	0.043538
SMC5	0.828848	0.717699	0.957211	0.010612	0.039373
KLRC3	0.683093	0.509937	0.915047	0.010614	3.95E-05
ACBD4	0.800166	0.674381	0.949414	0.010623	0.03149
NHEJ1	1.441883	1.088924	1.909248	0.010629	0.007699
GRM6	4.593118	1.425345	14.80114	0.010662	4.33E-05
FUNDC1	0.73645	0.582206	0.931557	0.010734	0.040344
BAK1	1.400177	1.081049	1.813511	0.010758	0.022837
GEMIN7	1.45567	1.090801	1.942587	0.010763	0.008663
LTBP3	0.839302	0.733556	0.960291	0.010782	0.003948
BAP1	1.420483	1.084533	1.8605	0.010792	0.001495
GJA1	1.116823	1.025857	1.215854	0.010806	0.007601
SLC7A6	1.309713	1.064289	1.611733	0.010819	0.006413
RIOX1	1.293709	1.06124	1.577101	0.010831	0.011662
TPK1	0.744925	0.593923	0.934318	0.010842	0.004959
ESR1	0.680781	0.506417	0.915181	0.010863	0.00486
KIF1A	1.101911	1.022606	1.187368	0.01088	0.043046
CWF19L2	0.810217	0.68905	0.952691	0.010885	0.026291
PHB2	1.371587	1.075393	1.749361	0.010911	0.003116
SMPD4	1.471489	1.092731	1.981531	0.010959	0.005077
KLHL35	1.156946	1.033969	1.294549	0.011004	0.028322
RNF149	0.720321	0.55929	0.927716	0.011049	0.021119
HHEX	0.87684	0.792293	0.970411	0.011067	0.008656
AFAP1L2	0.909714	0.845666	0.978613	0.011074	0.005755
MDGA2	1.159238	1.034239	1.299343	0.01114	0.025132
NSUN6	0.787927	0.655444	0.947188	0.011161	0.018715
CD1C	0.8672	0.776826	0.968088	0.011163	0.013367
HADH	1.29215	1.059983	1.575169	0.011199	0.024311
HSPB1	1.235112	1.049159	1.454025	0.0112	0.01198
TMEM250	1.364629	1.073176	1.735235	0.01121	0.013004
RMDN2	0.76973	0.628747	0.942327	0.01123	0.01659
KDM4B	1.411896	1.081426	1.843352	0.011234	0.004831
CASP2	1.379766	1.075728	1.769736	0.011253	0.017651
SLC34A1	1.466803	1.090667	1.972657	0.011275	0.000888

GRHPR	1.37447	1.074697	1.757859	0.011282	0.000591
STK25	1.404194	1.079735	1.826151	0.011334	0.02501
POLR1G	1.33943	1.068259	1.679436	0.011339	0.024303
PMF1	1.37044	1.073692	1.749204	0.011372	0.032186
CCDC171	1.206238	1.04318	1.394782	0.011391	0.028578
PTGFRN	1.164219	1.034863	1.309745	0.011399	0.039224
DYNLL1	1.604669	1.112394	2.314793	0.011415	0.032012
DDA1	1.475421	1.091536	1.994317	0.011419	0.000405
WSB2	1.362779	1.072126	1.732229	0.011439	0.011731
BCL2A1	0.917251	0.857838	0.980779	0.011471	0.002664
SLC41A2	0.822653	0.707102	0.957086	0.011473	0.027177
SAR1B	0.754136	0.605914	0.938617	0.011493	0.035138
MRPL28	1.386633	1.075799	1.787277	0.011598	0.04652
KCTD10	1.442424	1.085223	1.917198	0.011625	0.03257
NCLN	1.379321	1.074304	1.77094	0.011667	0.008572
CDH24	1.18248	1.038042	1.347016	0.01168	0.004611
RRP36	1.416506	1.080521	1.856964	0.011716	0.022362
TRAPPC2	0.744936	0.592431	0.936699	0.011752	0.005965
KNOP1	1.335446	1.066317	1.672501	0.011763	0.002976
MED24	1.389851	1.075808	1.795567	0.011764	0.025513
WWP1	0.78554	0.650996	0.94789	0.011793	0.031766
DCTN1	1.423083	1.080833	1.873708	0.011944	0.04087
POPDC2	0.807313	0.683204	0.953968	0.01196	0.032127
TGFB1	0.857589	0.760752	0.966753	0.011969	0.004783
DDT	1.284429	1.056603	1.56138	0.011983	0.000892
TRAP1	1.325584	1.063944	1.651566	0.011988	0.003815
VOPP1	0.870691	0.781517	0.970041	0.012015	0.030818
VPREB3	0.893324	0.818038	0.975538	0.012028	0.001862
DCAF7	1.35864	1.069547	1.725874	0.012047	0.004247
PLCB3	1.310093	1.061043	1.6176	0.012047	0.009904
VANGL1	1.217188	1.04402	1.419079	0.012068	0.008714
SIDT1	0.817246	0.698071	0.956767	0.012089	0.011927
RPTOR	1.339362	1.065997	1.68283	0.012118	0.002277
PARVB	1.241366	1.048416	1.469827	0.012124	0.000158
BIRC5	1.216161	1.043657	1.417177	0.01216	0.039849
CACNB3	1.240003	1.04807	1.467084	0.01217	0.01475
TEX264	1.373354	1.071568	1.760132	0.012212	0.008921
KLKB1	0.777223	0.638112	0.946659	0.012254	0.001158
RPUSD1	1.333841	1.064604	1.671169	0.012274	0.028936
TRPM2	0.868464	0.777698	0.969824	0.01228	0.008786
PRKCB	0.884568	0.803563	0.973739	0.012313	0.012745
MEDAG	0.874436	0.787219	0.971317	0.012321	0.021331
FANCG	1.298238	1.058193	1.592737	0.012342	0.021941
NUDT15	1.269599	1.052972	1.530792	0.012394	0.00099
BTBD19	0.845111	0.740659	0.964294	0.012415	0.001476
FBXO4	0.800691	0.672478	0.953349	0.012541	0.020514
CLEC17A	0.814018	0.692582	0.956747	0.012547	9.62E-05
NCOA5	1.406645	1.076037	1.838831	0.012558	0.007737
ZIK1	1.19169	1.038379	1.367637	0.012561	0.002747
DNAJB1	1.324442	1.062166	1.651482	0.012574	0.031318
GPR55	0.800453	0.672061	0.953373	0.012586	0.000684

NATD1	1.251613	1.049319	1.492908	0.012589	0.017067
APCDD1	1.086666	1.017952	1.160017	0.012637	0.021531
AURKC	0.787903	0.653246	0.950318	0.012672	0.006472
BRF1	1.467952	1.085431	1.985278	0.012696	0.02525
PAPSS2	1.142356	1.028782	1.268468	0.012736	0.020545
ACO2	1.265653	1.051477	1.523455	0.012752	0.01692
DHX33	1.247969	1.048295	1.485675	0.012768	0.011003
DNAJC5	1.375049	1.070125	1.766859	0.012781	0.001015
CECR2	1.176446	1.03515	1.33703	0.012805	0.012587
KYNU	0.82564	0.710018	0.960089	0.012807	0.001023
TMED3	1.328788	1.062287	1.662146	0.012809	0.041865
VEGFB	1.274539	1.052811	1.542963	0.012856	0.029283
TRIM59	0.766373	0.621263	0.945376	0.012975	0.037147
PAQR5	1.167537	1.033155	1.319398	0.013036	0.004131
TGFBRAP1	1.294336	1.055822	1.586731	0.01304	0.00594
ALKBH2	1.280952	1.053507	1.5575	0.013043	0.003536
DPP9	1.368801	1.068135	1.754101	0.013107	0.003438
ANKRD29	0.814977	0.693324	0.957976	0.013121	0.010473
RPLP0	1.279075	1.053005	1.553681	0.013122	0.049522
SYT7	1.151706	1.029983	1.287814	0.0132	0.000231
DCTN5	1.463886	1.082969	1.978784	0.013201	0.000615
CTD-2370N	0.51636	0.306082	0.871098	0.013243	0.049707
SFTPC	1.109035	1.021823	1.203691	0.013265	0.027
STRIP1	1.386843	1.070484	1.796695	0.013303	0.010861
PRC1	1.231081	1.044228	1.45137	0.013314	9.54E-05
RAB39B	0.839783	0.731338	0.96431	0.013319	0.00468
RPS19	1.270447	1.05107	1.535613	0.013325	0.001198
PAFAH1B3	1.249821	1.047189	1.491662	0.013479	0.005299
GPC3	1.093406	1.01863	1.17367	0.013485	0.024968
SOGA1	1.31616	1.05842	1.636663	0.01349	0.000526
MRPL12	1.238205	1.045082	1.467015	0.013525	0.009552
EOMES	0.883167	0.800215	0.974719	0.013557	0.037458
CFAP77	1.237456	1.044868	1.465542	0.013569	0.047819
SLC25A53	0.674858	0.493844	0.92222	0.013581	0.022067
ADAP2	0.857368	0.758733	0.968824	0.013592	0.001339
IMP4	1.45005	1.079313	1.948134	0.013641	0.045633
NFKBIZ	0.870683	0.779928	0.971999	0.013676	0.018328
CSF2	0.736567	0.57761	0.939268	0.013697	0.000756
USP22	1.277106	1.051391	1.551278	0.013701	0.013159
MX1	0.904098	0.834424	0.979589	0.013741	0.020212
NEIL2	1.297497	1.054638	1.596281	0.013775	0.012049
STRN4	1.451913	1.079132	1.953469	0.013778	0.000254
MED22	1.371659	1.066646	1.763892	0.013787	0.017259
PITX2	1.134216	1.026017	1.253826	0.013814	0.014662
DUSP3	1.230569	1.043133	1.451684	0.013862	0.005742
MPPED1	1.354801	1.063734	1.725511	0.013869	0.039078
ADAMTS2	1.128521	1.024904	1.242613	0.013871	0.013012
FKBP5	0.890951	0.812642	0.976805	0.013896	0.006853
MBP	1.11457	1.02226	1.215215	0.013929	0.043791
DCLRE1C	0.793593	0.660042	0.954165	0.013932	0.036444
RSPO3	0.820832	0.701212	0.960857	0.014017	0.006552

CDK14	0.875403	0.787229	0.973453	0.014023	0.004169
RHOBTB1	1.196792	1.036899	1.38134	0.014081	0.001101
CARS2	1.34925	1.062228	1.713827	0.014102	0.013164
KIF2C	1.19922	1.037246	1.386487	0.014131	0.013712
OTOF	0.614886	0.416885	0.90693	0.014181	0.006561
GLIPR2	0.857601	0.758501	0.969648	0.014209	0.004662
ACOX2	0.872747	0.782748	0.973093	0.01424	0.021797
U2AF2	1.461817	1.07902	1.980416	0.01425	0.001703
GPSM1	1.190325	1.035504	1.368293	0.014257	0.037327
TP53I3	1.189824	1.035348	1.367347	0.014304	0.004926
IGHV3OR16	0.853387	0.751712	0.968815	0.014308	0.012951
CFAP65	1.452101	1.077162	1.957548	0.014376	0.013237
BABAM1	1.515264	1.086326	2.113567	0.01438	0.005463
NOP9	1.352052	1.06176	1.721712	0.014448	0.028119
CHKA	1.240016	1.043645	1.473337	0.014461	0.015075
TNKS1BP1	1.258893	1.046768	1.514004	0.014467	0.03728
COG8	1.344747	1.060524	1.705143	0.014485	0.001006
CR2	0.903591	0.833064	0.980089	0.014485	0.000174
FBLN1	1.11879	1.022492	1.224156	0.014512	0.003901
TENM1	0.738302	0.578845	0.941685	0.014527	0.003342
ZBTB39	1.26816	1.048068	1.53447	0.014578	0.024911
NDUFV3	1.376228	1.065111	1.778222	0.01459	0.016789
SLC15A2	0.812726	0.688138	0.95987	0.014591	0.024281
NF2	1.354701	1.061771	1.728447	0.014601	0.002348
CARD10	1.158058	1.029294	1.30293	0.014684	0.016613
BLK	0.849202	0.744702	0.968366	0.014697	0.00216
IL20RA	1.23071	1.041667	1.45406	0.014698	0.008392
MAP2K1	0.743663	0.586174	0.943466	0.014717	0.035664
RFXAP	0.807578	0.680136	0.9589	0.014732	0.038562
GTF2IRD1	1.265586	1.04726	1.529427	0.014772	0.019328
MEPCE	1.373143	1.063894	1.772283	0.014863	0.044032
RAP1GAP	1.121162	1.022535	1.229302	0.01492	0.003331
CKS1B	1.28102	1.049429	1.563719	0.014926	0.011465
HSF5	0.395451	0.187345	0.834724	0.014937	0.000532
TBCD	1.32695	1.056533	1.666579	0.014977	0.005435
CSRNP1	0.817011	0.694204	0.961543	0.015022	0.014485
MPV17L2	1.327823	1.056544	1.668756	0.015029	0.012336
CARD9	0.780953	0.639817	0.953222	0.015057	0.002886
MED20	1.281753	1.049234	1.565801	0.015076	0.004405
TRIM16	1.261945	1.046066	1.522375	0.01508	0.003054
E2F2	1.242647	1.042917	1.480628	0.015099	0.005535
SYVN1	0.69655	0.52033	0.932451	0.015102	0.022459
MSC	1.096862	1.018033	1.181795	0.015113	0.027118
BNIP2	0.794976	0.660618	0.95666	0.015139	0.021755
METTL7A	0.884976	0.801855	0.976714	0.015175	0.010103
SULT1A1	0.866717	0.772154	0.97286	0.015234	0.038393
ARHGEF17	1.208817	1.037114	1.408947	0.015258	0.032588
MPZL3	0.842113	0.73289	0.967614	0.015331	0.000987
RPS3	1.21556	1.038056	1.423417	0.015363	0.006508
TBX1	1.183898	1.032719	1.357209	0.015442	0.029454
SNAI2	1.125462	1.022779	1.238453	0.015461	0.025114

SYCE3	0.824169	0.704675	0.963927	0.015534	0.030594
EGLN2	1.501617	1.080201	2.087439	0.015563	0.03107
POLH	1.242992	1.04209	1.482627	0.015592	0.016454
KLHL30	1.163537	1.029044	1.315609	0.015658	0.023514
CCL24	0.867334	0.772777	0.973461	0.015663	0.03507
PTGS1	0.867428	0.772898	0.97352	0.015699	0.004625
NDUFB7	1.299073	1.050553	1.606384	0.015727	0.010476
RETREG2	1.378032	1.062291	1.78762	0.015731	0.014147
RCCD1	1.29294	1.049458	1.592912	0.015803	0.008829
RETREG1	0.898597	0.823845	0.980132	0.015829	0.018148
NDUFB9	1.295087	1.04972	1.597806	0.015834	0.022742
CCL22	0.866477	0.771245	0.973469	0.015839	0.003516
PGBD4	0.735613	0.573189	0.944062	0.015858	0.011627
ZMYND19	1.316537	1.052834	1.646291	0.01589	0.011816
GNE	0.804688	0.674404	0.960139	0.015894	0.049036
NAA20	0.743864	0.584804	0.946186	0.015926	0.024985
MDGA1	0.849643	0.744146	0.970095	0.016005	0.017072
AKAP12	0.904523	0.833587	0.981495	0.016031	0.033552
AGFG2	0.762344	0.611258	0.950775	0.016046	0.013232
CORO1C	1.28674	1.047921	1.579986	0.016092	0.009281
THAP4	1.405369	1.065112	1.854323	0.016131	0.018042
CTD-2643I	0.487508	0.271482	0.875433	0.016156	0.02828
MTSS1	0.886607	0.803765	0.977986	0.016184	0.043736
DHX37	1.334665	1.054843	1.688718	0.016185	0.017387
PPM1G	1.391835	1.062942	1.822493	0.016228	0.005474
CENPA	1.198033	1.033904	1.388216	0.016239	0.040701
CELSR1	1.119353	1.021009	1.22717	0.016257	0.002186
GLE1	1.406848	1.064866	1.858657	0.016294	0.022751
PLN	0.901961	0.829111	0.981211	0.016332	0.045773
ILF3	1.410545	1.065164	1.867917	0.016372	0.005061
EVI5L	1.306857	1.050326	1.626044	0.016379	0.003188
EIF3C	1.336214	1.054555	1.6931	0.016406	0.002326
DOK4	1.198965	1.033793	1.390528	0.016421	0.00898
LY96	0.901462	0.82821	0.981194	0.01644	0.009347
SEZ6L2	0.925183	0.868231	0.98587	0.016442	0.023993
ZCWPW1	0.814225	0.688346	0.963124	0.016464	0.003582
UBB	1.252402	1.041969	1.505333	0.016485	0.010872
MACC1	0.698519	0.520939	0.936633	0.016513	0.001253
EGFR	1.120181	1.020896	1.229123	0.016544	0.017879
ST3GAL6	0.885306	0.801349	0.97806	0.016559	0.00099
KDF1	1.186075	1.031527	1.363778	0.016587	0.018959
SLC25A10	1.199402	1.033599	1.391802	0.016606	0.049734
AIFM1	1.324526	1.052391	1.667031	0.016614	0.001511
ANKRD45	0.862167	0.763631	0.973418	0.016618	0.00198
CRYBG1	0.8902	0.80931	0.979174	0.016712	0.005764
MMP16	0.919861	0.859012	0.985021	0.016748	0.016838
IDNK	0.787028	0.646788	0.957675	0.016762	0.026594
SPAG1	0.828599	0.710253	0.966663	0.016795	0.014315
FRMD5	0.896621	0.819881	0.980544	0.016832	0.006786
KIAA0930	1.215621	1.035743	1.426739	0.016858	0.035145
FOSL2	1.174558	1.029337	1.340266	0.016877	0.00837

PSKH1	1.27602	1.044765	1.558461	0.016884	0.031227
AHNAK2	1.089747	1.015536	1.169381	0.016923	0.01014
NOTCH2	1.193173	1.032181	1.379275	0.016927	0.027103
GBF1	1.416208	1.064307	1.884462	0.01696	0.014872
RP11-403I	0.43539	0.219957	0.861825	0.016995	0.024628
FBXL16	0.879224	0.791016	0.977268	0.017021	0.005509
FAM177B	0.65876	0.467555	0.928158	0.017025	0.003253
DYNC1H1	1.324683	1.051382	1.669026	0.01708	0.006586
SKA3	1.192918	1.031927	1.379026	0.017086	0.008177
PDGFA	0.913107	0.847369	0.983946	0.017102	0.031456
UPB1	0.416189	0.202431	0.855664	0.017132	0.004452
OPTN	0.854895	0.751502	0.972512	0.017137	0.009444
SPEF2	0.765186	0.614039	0.953539	0.01714	0.028288
NCAPG2	1.228298	1.037168	1.45465	0.017179	0.009763
EAPP	0.738219	0.57509	0.947621	0.017208	0.039508
PRMT1	1.347948	1.054285	1.723409	0.017237	0.005256
LMO1	1.167011	1.027686	1.325225	0.017267	0.047276
VAV3	0.899104	0.823647	0.981474	0.017403	0.034038
ELAVL1	1.609268	1.087171	2.382095	0.017424	0.01824
AURKA	1.21703	1.035077	1.430967	0.017443	0.022808
KCNK5	0.89098	0.810092	0.979945	0.017446	0.019219
INSYN1	1.163054	1.026861	1.31731	0.017449	0.007305
RNASEH2B	0.790262	0.65077	0.959654	0.017521	0.028146
CISD3	1.334909	1.051804	1.694216	0.017536	0.049798
CASP4	0.834788	0.719218	0.968929	0.017543	0.00604
USH1G	1.265727	1.042038	1.537433	0.01755	0.010901
AOX1	0.856877	0.754268	0.973444	0.017618	0.001106
CD99	1.224697	1.035875	1.447938	0.017667	0.029056
QPRT	1.1382	1.022707	1.266736	0.017727	0.004125
CST3	0.827331	0.707318	0.967706	0.017764	0.011065
NRROS	0.87661	0.786159	0.977467	0.017781	0.002271
CSPG4	0.915781	0.851504	0.984911	0.017815	0.007574
AUNIP	1.211233	1.033662	1.419309	0.017823	0.015607
RPS5	1.26164	1.040895	1.5292	0.017865	0.030633
POLR2I	1.334867	1.051044	1.695332	0.017877	0.027647
SAV1	0.790081	0.6501	0.960202	0.017878	0.035647
BAX	1.331726	1.0506	1.688077	0.017886	0.003056
CA4	1.249668	1.039127	1.502869	0.0179	0.038114
CCDC51	1.30039	1.046266	1.616237	0.017902	0.00331
NOC4L	1.306091	1.046998	1.629301	0.01793	0.008306
THBS3	1.173133	1.02783	1.338977	0.017941	0.028216
ASB6	1.374084	1.056129	1.787763	0.017949	0.019266
HNRNPL	1.610337	1.085257	2.389465	0.017966	0.00752
LLGL1	1.284615	1.043707	1.581131	0.018094	0.031734
ISYNA1	1.164062	1.026287	1.320334	0.018095	0.041824
ZNF205	1.304894	1.046124	1.627672	0.018284	0.030166
C4BPB	0.403024	0.18943	0.857459	0.018315	0.0012
FAM186B	1.57226	1.079417	2.290125	0.018363	0.021632
CNTNAP3B	0.783938	0.640322	0.959764	0.018387	0.000905
KIF4A	1.20291	1.03164	1.402614	0.018401	0.037763
AKAP13	0.795485	0.657634	0.962233	0.018449	0.017269

MAP2K2	1.365591	1.053813	1.769611	0.018456	0.024307
HIC2	1.223373	1.034448	1.446801	0.018489	0.018769
SEPTIN6	0.874617	0.78228	0.977853	0.018603	0.012282
SAC3D1	1.251733	1.038233	1.509137	0.018613	0.008024
FUS	1.394612	1.057057	1.83996	0.018652	0.001184
ADAP1	0.850908	0.743757	0.973495	0.018716	0.003678
ALG3	1.289772	1.043165	1.594677	0.018759	0.025258
CFTR	1.642516	1.085794	2.484689	0.018788	0.014002
NTHL1	1.281068	1.041867	1.575188	0.018829	0.003077
ARHGAP8	1.192044	1.02939	1.380399	0.018927	0.025065
MIEF1	1.307582	1.045104	1.635982	0.018983	0.000321
GPR160	0.821245	0.696646	0.968129	0.018988	0.00121
CLEC16A	1.428835	1.060443	1.925205	0.01899	0.014619
NT5DC4	0.463158	0.243418	0.881264	0.019023	0.001544
ARF3	1.443774	1.061932	1.962915	0.01911	0.015349
MYOZ3	1.299323	1.043725	1.617515	0.019135	0.014352
PRR5	1.164281	1.025137	1.322312	0.019167	0.002024
TMEM147	1.245496	1.036464	1.496685	0.019178	0.002723
ASPHD1	0.9058	0.833798	0.984019	0.019223	0.024948
PDE9A	1.127332	1.019661	1.246372	0.019278	0.001777
IGSF5	1.365406	1.051857	1.77242	0.019296	0.000914
ZNF416	1.328657	1.047206	1.685754	0.019297	0.004474
TUBB3	1.150207	1.022963	1.293278	0.019309	0.010069
PLSCR1	0.849843	0.74155	0.973951	0.01931	0.002309
SEMA6D	1.117393	1.018154	1.226305	0.019331	0.047436
NT5C	1.305713	1.044028	1.632991	0.019412	0.016862
PADI3	1.221929	1.032894	1.44556	0.019419	0.003122
RSRP1	0.85889	0.756018	0.975761	0.019442	0.026622
RPL35	1.260562	1.038049	1.530773	0.019449	0.034299
SSNA1	1.317821	1.045466	1.661128	0.019472	3.14E-05
PEBP1	1.276267	1.039948	1.566287	0.019549	0.020875
RP11-986E	0.847273	0.7372	0.973781	0.019588	0.001471
RGR	1.246866	1.03597	1.500695	0.019609	0.028377
KATNIP	1.221373	1.032447	1.44487	0.019679	5.46E-05
SFR1	0.786055	0.642075	0.962323	0.0197	0.00577
AMMECR1L	1.296071	1.042206	1.611774	0.01972	0.041136
FST	0.921188	0.859742	0.987026	0.019768	0.029734
PCSK1	0.915949	0.850745	0.98615	0.0198	0.010289
FZD9	1.100948	1.015326	1.193791	0.019904	0.04107
SLC25A44	1.314507	1.044182	1.654816	0.019911	0.033966
SLC25A11	1.367799	1.050616	1.780739	0.019977	0.003841
FOXL2	1.146669	1.021802	1.286794	0.019986	0.005147
PPCS	0.741216	0.575939	0.953922	0.019995	0.026215
XXYLT1	1.255667	1.036404	1.521316	0.020064	0.03435
GPR35	0.687431	0.501207	0.942846	0.020069	0.037274
MCM4	1.22598	1.032458	1.455776	0.020105	0.008405
MARVELD1	1.22032	1.031656	1.443485	0.020144	0.005883
TMEM123	0.860525	0.758108	0.976777	0.020157	0.032054
HRAS	1.269096	1.037956	1.551708	0.020172	0.000873
TRPS1	0.86762	0.769563	0.97817	0.020305	0.021329
SEC13	1.40818	1.054427	1.880614	0.020394	0.026684

EMC8	1.400548	1.053525	1.861879	0.020401	0.009112
PYCR1	1.196077	1.0281	1.391498	0.020402	0.001193
PIGW	1.288922	1.039998	1.597427	0.02044	0.00602
TMEM161A	1.289456	1.040046	1.598677	0.02045	0.003525
IGFBP1	0.711998	0.534244	0.948893	0.020453	0.004231
ISCU	0.71049	0.532149	0.9486	0.020461	0.044155
MKS1	1.322899	1.044102	1.676141	0.020484	0.045456
MBNL3	0.863713	0.763002	0.977716	0.020546	0.008414
UBE2L3	1.422455	1.05569	1.916641	0.020549	0.017776
HASPIN	1.22763	1.031776	1.46066	0.020738	0.043052
APLN	1.13501	1.019421	1.263705	0.020835	0.009557
ZNF629	1.257996	1.035358	1.528508	0.020909	0.011958
CDC45	1.174057	1.02455	1.345381	0.020946	0.000144
RFK	0.80671	0.672185	0.968157	0.02102	0.049584
RBM19	1.397905	1.051757	1.857977	0.021022	0.005184
CHI3L2	0.896315	0.816722	0.983665	0.02105	0.017442
NPB	4.201041	1.240562	14.22641	0.021092	0.035019
COL3A1	1.095025	1.013681	1.182897	0.021167	0.005831
MAOB	0.93154	0.876987	0.989487	0.021267	0.044647
NUP62	1.515018	1.063854	2.157514	0.021272	0.040747
SPRY1	0.902547	0.827091	0.984887	0.021344	0.030002
FGF13	0.85983	0.756053	0.977852	0.021378	0.047273
SETD1B	1.382136	1.048918	1.82121	0.021489	0.030632
BSG	1.231274	1.031155	1.47023	0.021506	0.030133
EPHX1	1.131176	1.018307	1.256555	0.021548	0.03246
UBE2M	1.326969	1.042515	1.689038	0.02155	0.029677
TMEM106B	0.83858	0.721656	0.974448	0.021573	0.046101
SORCS1	0.920163	0.857111	0.987854	0.021596	0.009996
MRGPRX3	0.935784	0.884237	0.990337	0.021685	0.043818
BICRA	1.394214	1.049741	1.851725	0.02172	0.026915
NTAN1	1.367215	1.046675	1.78592	0.021754	0.000846
NCOA6	1.362681	1.046156	1.774976	0.02176	0.045151
IL26	0.448708	0.226202	0.890083	0.021839	0.021744
ZBED3	1.193588	1.026034	1.388504	0.02185	0.005578
CNEP1R1	0.765188	0.608699	0.961908	0.021868	0.03405
RAP2B	1.278782	1.036324	1.577965	0.021871	0.038909
CA13	0.828918	0.706042	0.973178	0.0219	0.011753
CHAMP1	1.224275	1.029715	1.455596	0.02193	0.002527
GKAP1	0.804986	0.668575	0.96923	0.022029	0.045514
HHIPL1	1.184375	1.024676	1.368965	0.022031	0.008353
EDC3	1.363655	1.045585	1.778483	0.022084	0.006007
PLEKHA5	1.141003	1.019057	1.277542	0.022178	0.021969
MFSD12	1.125209	1.016986	1.244949	0.02223	0.009876
TSFM	1.227626	1.029537	1.463829	0.022361	0.04045
SLC6A9	1.13896	1.018626	1.273509	0.022378	0.002714
DUSP26	0.740252	0.57175	0.958415	0.022474	0.009094
ARHGAP23	1.14996	1.019859	1.296658	0.02255	0.013931
CACNA1I	0.673574	0.479566	0.946067	0.022618	0.000348
CAMK2N1	0.908936	0.837286	0.986717	0.022658	0.016033
NLN	1.236928	1.030226	1.485102	0.022658	0.006835
MYB	0.801861	0.663174	0.969552	0.022661	0.034397

ITGA4	0.905108	0.830725	0.98615	0.022683	0.016308
LRRC41	1.371989	1.045221	1.800916	0.02269	0.002027
RND1	0.75653	0.595092	0.961762	0.022707	0.004365
FOLR2	0.896501	0.816044	0.98489	0.022768	0.04008
SCG2	0.916083	0.84951	0.987873	0.02279	0.013673
SH3BP5L	1.30105	1.037284	1.631889	0.022808	0.031516
MAGEA1	0.942054	0.894841	0.991757	0.022877	0.001979
HES4	1.134446	1.017619	1.264686	0.02291	0.023863
HSPBP1	1.270541	1.033531	1.561903	0.023023	0.003618
MICAL2	1.126191	1.016486	1.247735	0.023046	0.016943
RAB23	0.847331	0.734494	0.977502	0.023083	0.010917
PMFBP1	0.750337	0.585649	0.961337	0.023097	0.032111
CFAP46	1.559962	1.062873	2.289531	0.02312	0.000418
CDC6	1.192936	1.024475	1.389098	0.02313	0.000285
TFPI2	0.936933	0.885699	0.991131	0.023179	0.002905
SOX6	1.101441	1.013263	1.197293	0.023242	0.025596
TLE4	0.892969	0.809792	0.984689	0.023253	0.001267
MTTP	0.764922	0.606837	0.964189	0.023287	0.016852
CFH	0.892948	0.809735	0.984712	0.02329	0.006316
COPZ1	1.470553	1.053795	2.052133	0.023321	0.01066
SPSB1	1.177529	1.022415	1.356175	0.023356	0.032573
UTP4	1.285402	1.034583	1.597029	0.023394	0.027106
C16orf95	1.316507	1.037917	1.669873	0.023406	0.038617
GNAO1	1.100021	1.01298	1.194542	0.023415	0.016619
RP11-176H	1.819689	1.084329	3.053748	0.023422	0.030384
NKPD1	1.224886	1.027715	1.459884	0.023497	0.005516
ABCB8	1.271344	1.032876	1.564869	0.023502	0.00214
C12orf43	1.490368	1.055222	2.104956	0.023507	0.02228
SLC45A3	1.161551	1.020229	1.322448	0.023664	0.036958
KCNQ4	1.145559	1.018314	1.288704	0.023694	0.005224
RACGAP1	1.235268	1.028582	1.483485	0.023721	0.001549
ZBED4	1.251341	1.03035	1.519732	0.023727	0.003722
SIX2	1.120706	1.015244	1.237123	0.023822	5.55E-05
RDH13	1.288178	1.034131	1.604633	0.023857	0.00643
NIBAN3	0.836433	0.716358	0.976633	0.023883	0.001225
SFSWAP	1.402061	1.045432	1.880349	0.02403	0.012221
SNX12	1.4197	1.047149	1.924797	0.024031	0.028257
AIP	0.767901	0.610481	0.965914	0.024055	0.014364
PHPT1	1.243739	1.029042	1.503229	0.024066	0.03747
FAM163B	0.770738	0.61465	0.966464	0.024108	0.035955
REEP4	1.270972	1.031869	1.565481	0.024134	0.03679
MCRIP1	1.237213	1.02822	1.488686	0.02415	0.002923
LTBR	1.263555	1.031056	1.548483	0.02415	0.025456
EML3	0.748311	0.581597	0.962813	0.024155	0.04402
NEO1	1.116895	1.014532	1.229587	0.024188	0.008123
IRGQ	1.29757	1.03452	1.627508	0.024224	0.005365
FOXL2NB	1.195324	1.0235	1.395994	0.024239	0.004272
KXD1	1.47772	1.052143	2.075436	0.024243	0.013174
COLQ	0.815958	0.683637	0.97389	0.024257	0.047245
CFAP91	0.75026	0.58424	0.963456	0.024341	0.011599
HS3ST2	1.093615	1.011645	1.182227	0.024372	0.007992

CRIP3	0.715053	0.533936	0.957606	0.024408	0.00921
VPS33B	1.317051	1.035965	1.674403	0.024548	0.005876
TNPO2	1.335597	1.037813	1.718824	0.024555	0.007938
TEX30	1.193058	1.022879	1.39155	0.024573	0.012401
POU2F3	1.234054	1.027264	1.482472	0.024613	0.03966
ARRDC3	0.887863	0.800368	0.984924	0.024644	0.045907
RNF25	1.443546	1.047925	1.988525	0.024677	0.018869
PIMREG	1.1761	1.020868	1.354937	0.024708	0.035125
FMC1-LUC7	5.692542	1.24647	25.99745	0.024815	0.044585
STOML3	1.244206	1.028018	1.505857	0.02485	0.018491
RP11-426I	0.267814	0.084699	0.84681	0.024893	0.028137
PRR12	1.335638	1.0372	1.719947	0.024894	0.006195
CABP4	0.862436	0.757759	0.981573	0.024982	0.018595
SNAP91	0.57929	0.359378	0.933771	0.025009	0.016796
RTL8C	1.296038	1.032981	1.626085	0.025071	0.027754
BIN1	0.88251	0.791092	0.984492	0.025085	0.005323
ENTR1	1.288951	1.032034	1.609825	0.025223	0.021592
CRKL	1.293136	1.032331	1.61983	0.025297	0.012469
F7	1.202153	1.023035	1.412634	0.025312	0.003191
GIT2	0.8057	0.666697	0.973685	0.025354	0.042171
PRRG3	1.267898	1.029714	1.561175	0.025366	0.000249
CLEC4C	0.719551	0.539157	0.960303	0.025415	0.008774
PGBD1	1.201391	1.022814	1.411147	0.025437	0.005573
ZNF496	1.228643	1.025518	1.472001	0.025532	0.003626
B3GAT2	0.483833	0.255841	0.914999	0.025535	0.037988
HJURP	1.173056	1.019677	1.349506	0.025581	0.003388
ALDH1L2	1.113174	1.013173	1.223046	0.025584	0.049355
CHD7	1.202844	1.022798	1.414583	0.025586	0.00501
SYNGR2	0.817022	0.684076	0.975805	0.02573	0.013669
PTCRA	0.879853	0.786244	0.984607	0.02573	0.015125
LZTR1	1.248655	1.027148	1.517931	0.025827	0.032555
GPC1	1.090185	1.010423	1.176243	0.025916	0.003348
ZNF768	1.309595	1.032884	1.660438	0.025939	0.048947
MEFV	0.721277	0.541027	0.96158	0.025947	0.046299
ZWINT	1.214151	1.023488	1.440333	0.025992	0.01054
GPRIN1	1.138859	1.01566	1.277002	0.026016	0.011145
PXYLP1	0.82661	0.698986	0.977535	0.026047	0.023835
HCFC1	1.341154	1.035637	1.736801	0.026051	0.02936
NPM2	1.094212	1.010796	1.184512	0.026056	0.040943
YARS2	1.351736	1.036515	1.762822	0.026102	0.00633
UHRF1	1.157472	1.017553	1.31663	0.026103	0.005509
ADAMTSL5	1.149177	1.01668	1.298941	0.026106	0.006785
COL9A2	0.901299	0.822427	0.987734	0.026141	0.025712
AP3D1	1.385217	1.039428	1.846039	0.026156	0.001032
MYBPH	0.634009	0.424265	0.947446	0.026191	0.000291
PCIF1	1.349591	1.036086	1.757959	0.026229	0.003941
GINM1	0.799878	0.656909	0.973962	0.026245	0.048023
MEGF6	1.134063	1.014959	1.267145	0.026267	0.009005
SPON1	1.080232	1.009142	1.156331	0.026286	0.038533
FSCN2	0.715638	0.532666	0.961461	0.026362	0.047955
BZW2	1.195714	1.021137	1.400138	0.026438	0.031249

BCO2	0.71185	0.527286	0.961016	0.026443	0.004109
AAR2	1.384729	1.038777	1.845897	0.026462	0.00701
FCER2	0.894271	0.810219	0.987041	0.026488	0.00291
UNC5B	1.139218	1.01533	1.278221	0.026489	0.010696
SLC7A2	1.104048	1.011575	1.204974	0.026567	0.047748
GJB4	1.216095	1.022893	1.445789	0.026665	0.000639
CYB561A3	1.166904	1.01801	1.337575	0.026674	0.026121
IFT74	0.855975	0.74596	0.982216	0.026718	0.047417
HEBP1	1.22722	1.023856	1.470978	0.02676	0.041661
ADCY6	1.169644	1.018176	1.343644	0.026792	0.003565
FAAP100	1.272121	1.027972	1.574258	0.026849	0.001577
FECH	1.265979	1.027369	1.560008	0.026873	0.022098
TBX15	1.148824	1.01601	1.298999	0.026873	0.012551
CCNB2	1.170594	1.018076	1.345961	0.027002	0.00313
C19orf67	0.342267	0.132307	0.885418	0.027042	0.042283
METTL9	1.194412	1.020308	1.398225	0.0271	0.044815
RIPPLY3	1.284307	1.028641	1.603519	0.027154	0.006199
ZFYVE21	1.281298	1.028365	1.596442	0.027157	0.045011
PGLS	1.287048	1.028585	1.610457	0.027357	0.046382
PTPN4	0.778653	0.623476	0.972452	0.027362	0.019831
MATK	0.864706	0.759911	0.983952	0.027426	0.009288
TM2D3	0.747372	0.576914	0.968196	0.027477	0.033443
FAM133A	0.915058	0.845588	0.990234	0.027553	0.026692
TELO2	1.277992	1.027334	1.589807	0.027661	0.000103
ADAM22	0.852409	0.739389	0.982704	0.02778	0.031419
IL1R1	0.881987	0.788624	0.986402	0.027822	0.019058
SSX1	0.932336	0.875872	0.99244	0.027945	0.013149
WBP11	1.300705	1.028828	1.644427	0.027983	0.017379
OTUD7A	1.258946	1.025191	1.546	0.027994	0.026741
NCOA1	0.787764	0.636731	0.974624	0.028046	0.026308
ZBTB12	1.171751	1.017216	1.349763	0.028055	0.004128
ATP5F1A	1.327404	1.030954	1.709097	0.028065	0.00409
DIAPH3	1.168495	1.016853	1.342751	0.02812	0.00036
KCNS1	1.109082	1.011159	1.216487	0.028145	0.005749
URB1	1.241623	1.02341	1.506362	0.028191	0.034895
ANKRD53	1.157729	1.015775	1.319521	0.028201	0.038816
OR13A1	0.285557	0.09323	0.874645	0.028201	0.024279
FAM86B1	1.259708	1.024797	1.548468	0.028338	0.038737
FBXL7	1.096122	1.009764	1.189866	0.028377	0.035385
PRDM4	1.318651	1.029699	1.688688	0.028389	0.040861
MBD6	1.268869	1.025429	1.570103	0.028451	0.016386
RECK	0.825847	0.695682	0.980366	0.028775	0.043897
NCL	1.338271	1.030596	1.737799	0.02881	0.000402
DKKL1	1.182478	1.017428	1.374303	0.028873	0.044878
BAHCC1	1.17337	1.016595	1.354323	0.028897	0.010322
SEPTIN9	1.270347	1.024853	1.574647	0.02896	0.004584
HBD	0.632998	0.419839	0.954382	0.029048	0.006476
PPIL6	1.237338	1.021914	1.498174	0.029104	0.027416
TMBIM6	1.404567	1.035176	1.905771	0.029109	0.01792
GADD45G	0.88436	0.791878	0.987643	0.029213	0.011597
RBIS	0.793906	0.645175	0.976924	0.029217	0.038762

CDH8	1.457627	1.038762	2.045391	0.029259	0.035214
CD68	0.846191	0.728207	0.983291	0.029265	0.010619
CHMP1B	1.235447	1.02156	1.494117	0.029266	0.03261
EXOC6B	1.258216	1.023423	1.546875	0.029282	0.030931
ALDH7A1	1.198588	1.018405	1.41065	0.029303	0.016156
MRPL57	1.349283	1.030202	1.767192	0.029548	0.002107
BAG6	1.317498	1.027714	1.688992	0.029581	0.01773
RGS2	0.905882	0.828687	0.990267	0.029616	0.002259
TMEM37	0.866343	0.761269	0.985919	0.029635	0.046815
AMDHD2	1.194704	1.017587	1.402648	0.029787	0.042139
CTD-2116N	4.214623	1.150742	15.43617	0.029859	0.006443
NKX2-2	1.102594	1.009516	1.204253	0.029973	0.048859
CD1E	0.838607	0.71534	0.983115	0.030014	0.037404
C1QBP	1.259639	1.022514	1.551754	0.030068	0.02539
UGT2B17	0.629519	0.414364	0.95639	0.030088	0.015038
ARRDC4	1.12967	1.011774	1.261305	0.03015	0.007425
ACCSL	1.115363	1.010494	1.231115	0.030221	0.015951
SIN3B	1.370368	1.030486	1.822352	0.030277	0.004991
ADAMTS15	1.125013	1.011277	1.25154	0.030297	0.003903
STK26	0.899377	0.817038	0.990012	0.030398	0.036452
HSD17B10	1.345136	1.02839	1.75944	0.03044	0.037639
NCAPD2	1.251626	1.021279	1.533928	0.030552	0.000349
BTN1A1	0.63173	0.41652	0.958135	0.03068	0.004772
INPPL1	1.285517	1.023626	1.614411	0.030706	0.004172
WNK4	0.925329	0.862398	0.992852	0.030805	0.007999
TREML2	0.801762	0.656078	0.979796	0.030815	0.000117
CHRNB2	1.114368	1.010024	1.229491	0.030865	0.016501
PTGR1	1.22653	1.018865	1.476521	0.030972	0.002856
CFAP54	0.446751	0.214821	0.929085	0.031017	0.003364
E2F5	0.834948	0.708707	0.983676	0.031026	0.023018
BACE2	1.179706	1.015108	1.370992	0.031119	0.000125
EEF1AKMT1	1.218594	1.018069	1.458615	0.031148	0.045074
ATP1A2	1.099109	1.008592	1.197748	0.031156	0.018845
SLC25A5	1.235762	1.019266	1.498241	0.031228	0.006133
PAICS	1.249292	1.019853	1.530348	0.031567	0.013775
COL6A3	1.106231	1.008931	1.212915	0.031615	0.001574
ATP8A1	0.897891	0.813892	0.99056	0.031616	0.011305
PRRT2	0.853193	0.738092	0.986243	0.031768	0.005222
TSPAN18	1.129132	1.010619	1.261542	0.03182	0.03741
MNT	1.380225	1.02833	1.852538	0.031872	0.030306
ARL4C	0.890515	0.801017	0.990014	0.031898	0.015344
MR1	0.861272	0.751414	0.987193	0.031944	0.010907
PSENEN	1.289667	1.022108	1.627265	0.032009	0.014693
EPHB2	0.840829	0.717592	0.98523	0.032034	0.04748
MANBA	0.859084	0.747691	0.987071	0.032064	0.008059
GABPB1	0.745893	0.570502	0.975204	0.03207	0.044411
KIF24	1.26643	1.020405	1.571771	0.032093	0.024345
ARHGDIA	1.278043	1.021149	1.599564	0.032132	0.048571
WFDC1	1.072115	1.005955	1.142626	0.03214	0.003426
ALK	0.880688	0.784046	0.989243	0.032167	0.000853
TGDS	0.876071	0.776182	0.988815	0.032188	0.027047

NMUR1	0.790639	0.637708	0.980246	0.032203	0.002019
MT1X	0.881105	0.784655	0.989411	0.03236	0.002773
RAD51	1.220028	1.016764	1.463927	0.032456	0.002425
C19orf18	1.215561	1.016348	1.453821	0.032555	0.023433
SIGLEC6	0.671729	0.466233	0.967798	0.032708	0.034537
F5	0.918026	0.848696	0.993018	0.032774	0.001416
GUF1	1.252382	1.018494	1.539981	0.032867	0.007503
NEURL1	1.123175	1.009495	1.249656	0.03288	0.005932
RRP1B	1.275056	1.019936	1.59399	0.032903	0.003374
ELOF1	1.36219	1.025304	1.809767	0.032977	0.025254
AFG3L2	1.212172	1.015649	1.446721	0.033005	0.036668
POMK	1.168786	1.012656	1.348988	0.033017	0.045614
ZNF649	1.16501	1.012389	1.340638	0.033021	0.007642
CHRFAM7A	1.510288	1.033656	2.206701	0.033084	0.020258
RBM28	1.306432	1.021517	1.670813	0.033207	0.012619
CH507-9B2	1.244409	1.017341	1.522158	0.033402	0.012545
FBXO16	0.814888	0.674812	0.98404	0.033408	0.002969
KLF16	1.240763	1.017083	1.513635	0.033422	0.007673
MPRIP	1.287491	1.02001	1.625115	0.033444	0.00283
TMEM141	1.234281	1.016599	1.498574	0.033481	0.027897
REPIN1	1.296432	1.02051	1.646956	0.033483	0.009874
ZNF875	1.262877	1.018391	1.566057	0.033506	0.000715
E2F3	1.21243	1.01513	1.448078	0.03353	0.001506
IDH3B	1.321939	1.021945	1.709996	0.033565	0.035314
LEPROT	0.830187	0.699123	0.985821	0.033765	0.042534
RAB3IP	0.848191	0.728501	0.987546	0.033885	0.01748
ARX	1.150512	1.010711	1.309651	0.03391	0.001139
CCDC200	0.707537	0.513875	0.974185	0.033985	0.046271
GALNTL6	1.141029	1.009979	1.289082	0.034049	0.004494
SERPINE1	0.913137	0.839532	0.993195	0.034072	0.02291
KRT4	1.083451	1.005972	1.166898	0.034239	0.022684
GALK2	0.791178	0.636926	0.982788	0.03427	0.021661
MRPS34	1.290028	1.018973	1.633187	0.034333	0.031612
KCNJ11	0.808349	0.663746	0.984453	0.034364	0.016357
MARCHF4	1.196764	1.01319	1.413599	0.034498	0.006298
JDP2	0.822101	0.685531	0.985878	0.034566	0.01532
PCSK2	1.068108	1.004783	1.135424	0.034601	0.017206
OSBP2	0.891263	0.800968	0.991737	0.034669	0.006088
XXbac-BPC	1.760925	1.041048	2.978591	0.034861	0.027001
STUB1	1.268664	1.017029	1.582558	0.034887	0.030917
FAM209B	0.716199	0.525196	0.976667	0.034932	0.04766
JUNB	0.855376	0.73979	0.989022	0.034945	0.023149
RIBC1	0.743311	0.564137	0.979392	0.035037	0.025436
TNFRSF11E	0.8949	0.8071	0.992253	0.035067	3.61E-05
MAFB	0.881939	0.784683	0.991248	0.035081	0.033814
CABLES1	1.093956	1.006251	1.189305	0.035194	0.001788
TMEM94	1.287734	1.01757	1.629627	0.035296	0.005334
RECQL4	1.18049	1.011458	1.37777	0.035339	0.005288
ZNF697	1.124604	1.007897	1.254825	0.03567	0.043594
GPA33	0.666192	0.45604	0.973186	0.035683	0.002249
ERVMER34-	1.129437	1.008171	1.26529	0.035694	0.033164

TMBIM4	0.772282	0.60658	0.983249	0.035989	0.015345
IL1RAP	0.903107	0.820977	0.993453	0.036173	0.023326
MTCL1	1.150679	1.008991	1.312262	0.036306	0.00509
ELOB	1.285503	1.015945	1.626582	0.036464	0.023276
CDKN1C	1.104069	1.006179	1.211483	0.036618	0.02061
KCNH7	0.072157	0.006124	0.850254	0.03672	0.025307
FOXP4	1.222326	1.012269	1.475972	0.036913	0.005879
MRPL9	1.372611	1.019325	1.848341	0.036975	0.040805
RAD54L	1.174495	1.009719	1.366163	0.037036	0.024492
CORO7-PAM	5.101623	1.100393	23.65205	0.037324	0.017529
RAI1	1.200774	1.010736	1.426542	0.037393	0.019366
ZNF598	1.271882	1.014049	1.595271	0.037465	0.001593
SCAP	1.299138	1.015234	1.662435	0.037513	0.023009
ABT1	1.323965	1.016285	1.724793	0.037555	0.005075
TLR5	0.830981	0.697738	0.989669	0.037854	0.03544
IL9R	0.652498	0.436054	0.97638	0.037875	0.016537
MCOLN1	1.254253	1.012708	1.553409	0.037924	0.002751
RETREG3	1.389544	1.018414	1.895922	0.037981	0.038738
AARS1	1.216603	1.010879	1.464195	0.038038	0.003533
YY2	0.804593	0.655162	0.988106	0.038069	0.007324
USP5	1.303646	1.01469	1.674889	0.038077	0.003402
ADGRA3	1.19188	1.009672	1.406969	0.038109	0.009462
CCNH	0.796831	0.642827	0.987729	0.038205	0.021113
ENPP2	0.921499	0.852939	0.99557	0.038217	0.025759
CDC16	1.206622	1.010153	1.441302	0.038324	0.045599
SOX4	1.135487	1.006853	1.280555	0.038331	0.021599
SULF1	0.919939	0.850058	0.995565	0.038431	0.025296
PHC1	1.230123	1.01107	1.496635	0.038453	0.004224
SYK	0.902341	0.818643	0.994597	0.038543	0.008622
TMEM92	1.200204	1.009317	1.427193	0.038932	0.003998
PLIN3	1.246954	1.011198	1.537674	0.039007	0.005021
SLC15A4	1.210196	1.00967	1.450548	0.03901	0.03357
KIF26A	1.132064	1.006203	1.273667	0.039131	0.02264
PINX1	1.170993	1.007799	1.360614	0.039264	0.018256
SNRPN	0.87917	0.777755	0.993809	0.039468	0.046503
ABAT	0.861027	0.746714	0.99284	0.03951	0.039832
PIR	1.084444	1.00387	1.171485	0.039588	0.007028
DCLK3	1.282645	1.011745	1.626081	0.039741	0.032057
PPM1M	0.839061	0.709839	0.991807	0.039747	0.027827
PPP1R11	1.303587	1.012372	1.678574	0.039852	0.004123
RTL8B	1.147439	1.00629	1.308386	0.040015	0.012418
CREBBP	1.302395	1.012092	1.675966	0.040036	0.002774
TMEM254	1.285434	1.011238	1.633977	0.040242	0.005781
USP43	1.181507	1.007395	1.385711	0.040311	0.023429
SAP30	0.857169	0.739752	0.993223	0.040322	0.022896
CRACR2B	0.846241	0.721332	0.99278	0.040472	0.026396
JARID2	1.198497	1.00788	1.425165	0.040484	0.001074
RNF167	1.351604	1.013141	1.803138	0.040488	0.023382
GET4	1.334695	1.012479	1.759456	0.040566	0.00687
EPM2A	1.176562	1.006964	1.374724	0.040624	0.015062
PEDS1	1.317163	1.011825	1.714643	0.040626	0.031263

AFF3	1.10616	1.00423	1.218435	0.040801	0.039585
NCSTN	1.276039	1.010245	1.611763	0.040806	0.023522
CCDC152	0.853107	0.732628	0.993397	0.04083	0.009468
ANKLE2	1.339991	1.01222	1.773898	0.040872	0.012543
RILPL1	1.271782	1.01002	1.601384	0.040879	0.011041
KLHL33	0.476055	0.23364	0.969991	0.040967	0.019706
ST8SIA2	1.097341	1.003775	1.199629	0.04107	0.002186
TUBA1B	1.23088	1.008445	1.502379	0.041087	0.00297
DYNLL2	1.274818	1.009804	1.609382	0.04115	0.011486
HUNK	1.114894	1.004369	1.237582	0.04117	0.035966
RP11-514(	0.766939	0.594476	0.989433	0.041181	0.027733
TNNI2	0.843113	0.715642	0.993289	0.041303	0.032491
IER5	1.227808	1.00792	1.495666	0.041518	0.034583
PRPF8	1.294008	1.009893	1.658053	0.04157	0.01006
POLD1	1.218025	1.007538	1.472486	0.041595	0.033413
ZNF316	1.263765	1.008948	1.582938	0.0416	0.017525
SNRPB	1.282294	1.009421	1.628932	0.041673	0.01361
UQCRH	1.238038	1.007604	1.52117	0.042148	0.041021
UBE2R2	1.31782	1.009675	1.720008	0.042274	0.01946
ZNF133	0.78038	0.614246	0.991449	0.042328	0.042772
PGM1	1.153247	1.004865	1.32354	0.042456	0.004889
ABCF2	1.304149	1.009056	1.685541	0.042474	0.003972
AP5B1	1.173558	1.005443	1.369783	0.042481	0.017913
TIGAR	1.206195	1.006328	1.445757	0.042539	0.040369
ADAMTS12	1.140444	1.004396	1.294919	0.042597	0.005537
ERV3-1	1.129887	1.004081	1.271455	0.042603	0.002021
KIAA1755	0.911661	0.833691	0.996924	0.042611	0.022479
PHACTR4	1.309784	1.009021	1.700196	0.042618	0.003176
TAMM41	1.385148	1.010868	1.898007	0.04264	0.014347
ELAPOR1	0.787716	0.625422	0.992124	0.042648	0.001173
IGSF3	1.109251	1.003423	1.22624	0.042687	0.043738
SRSF9	1.35918	1.010123	1.828858	0.042716	0.003022
ZNF746	1.317936	1.009101	1.721291	0.042717	0.038631
CPNE2	1.220629	1.006563	1.480221	0.042718	0.004411
WSCD1	1.145545	1.004467	1.306437	0.04272	0.02346
CTC-479C5	0.761836	0.585556	0.991184	0.042774	0.01012
ENPEP	1.165824	1.004997	1.352388	0.042787	0.009533
USP31	1.291583	1.008328	1.654408	0.042803	0.015322
ZNF814	1.261253	1.007533	1.578866	0.04282	0.042238
TBX5	0.827306	0.688573	0.99399	0.042936	0.048088
LRRC32	1.125266	1.00365	1.261617	0.043135	0.037351
SCLY	1.3782	1.009739	1.881117	0.043277	0.032032
SLC16A1	1.187849	1.005157	1.403748	0.043349	0.015883
GAN	1.256632	1.006383	1.569107	0.043787	0.039943
CRYM	1.114409	1.002992	1.238202	0.043846	0.019609
UBE2T	1.18857	1.004637	1.406178	0.04402	0.04499
ISL2	0.877436	0.772572	0.996533	0.044069	0.012491
RAB38	1.08257	1.001969	1.169656	0.044453	0.020027
MYG1	1.303732	1.006581	1.688603	0.044467	0.010759
BRI3BP	1.239222	1.00522	1.527696	0.044566	0.044387
PIK3IP1	0.885973	0.787244	0.997084	0.0446	0.001475

ERICH3	0.674372	0.459111	0.990562	0.044611	0.00715
LA16c-431	6.009255	1.043742	34.59777	0.044654	0.024546
COA7	1.240837	1.005121	1.53183	0.044698	0.012597
RFT1	1.355636	1.007213	1.824588	0.044709	0.008531
ARF5	1.304597	1.006206	1.691476	0.044787	0.031524
PHB	1.276753	1.005656	1.62093	0.044826	0.032574
GLI1	1.177572	1.003766	1.381474	0.044846	0.030204
KDM2B	1.341729	1.006684	1.788283	0.044919	0.0038
BEGAIN	1.124765	1.002636	1.26177	0.044979	0.006871
ALDH2	0.906612	0.823744	0.997817	0.045	0.004766
SNRPD2	1.315798	1.006106	1.720816	0.045025	0.003434
PAOX	0.837006	0.703231	0.996228	0.045231	0.043953
SESN2	1.18966	1.003621	1.410184	0.045325	0.046217
FAM131B	0.883992	0.783444	0.997444	0.045337	0.031132
WFS1	1.173864	1.003233	1.373516	0.045474	0.049515
DSCAML1	0.552987	0.309407	0.988323	0.045543	0.010729
CLU	0.939459	0.883675	0.998765	0.045552	0.016685
RYR1	1.112154	1.002021	1.234391	0.045725	0.008249
MOB3A	0.772007	0.598827	0.99527	0.045874	0.007777
SMYD5	1.340774	1.005199	1.788378	0.046016	0.00725
MYO1B	1.141915	1.002332	1.300935	0.046044	0.018019
INPP5E	1.26697	1.004068	1.598709	0.046133	0.004376
SYNGR1	1.150391	1.002391	1.320241	0.046156	0.008185
GBA	1.168746	1.002622	1.362395	0.046214	0.010448
ANTXR2	0.886492	0.787447	0.997995	0.046243	0.001107
C6orf136	1.279423	1.004023	1.630365	0.046324	0.039966
MCCC2	1.313098	1.004432	1.716618	0.046337	0.004407
TWNK	1.193616	1.002754	1.420806	0.046492	0.002659
BRD9	1.270079	1.003604	1.607309	0.046601	0.030689
OTC	0.452665	0.2073	0.988447	0.046687	0.001283
CHD4	1.302922	1.003772	1.691227	0.046784	0.015671
GDNF	0.936079	0.877058	0.999073	0.046825	0.048716
KCNE2	0.795398	0.63463	0.996893	0.046925	0.039069
MAP3K6	1.201582	1.002354	1.440407	0.047101	0.006062
AAAS	1.35705	1.003435	1.835279	0.047454	0.030307
ZNF324	1.35119	1.003347	1.819624	0.047483	0.032303
KLRF1	0.839057	0.705351	0.998107	0.047551	0.01936
LAMB4	1.322909	1.002999	1.744856	0.047573	0.000894
APOF	0.176791	0.031822	0.982186	0.047646	0.02749
TMEM203	1.395741	1.00319	1.941898	0.047831	0.013475
NLE1	1.240877	1.001973	1.536743	0.047925	0.01735
FAT1	1.130188	1.0011	1.275921	0.047959	0.003402
ANAPC7	1.348715	1.002351	1.814766	0.048215	0.008803
PAQR4	1.202185	1.001262	1.443428	0.048441	0.004087
SLC38A5	0.896302	0.803926	0.999291	0.048525	0.026593
RRP12	1.220326	1.001193	1.487422	0.048636	0.043673
TKTL1	1.07542	1.00043	1.156031	0.048653	0.002965
PMVK	1.254434	1.001237	1.57166	0.048757	0.006215
UCN	0.883784	0.78158	0.999353	0.048805	0.013024
ILRUN	1.227664	1.001004	1.505647	0.048885	0.002684
FAM180B	1.115664	1.000496	1.24409	0.048967	0.011012

AXIN1	1.303719	1.001185	1.69767	0.048981	0.000932
WWOX	1.200996	1.000646	1.441462	0.049195	0.043557
TCHP	1.33062	1.000566	1.769548	0.049547	0.032596
LDHB	1.194452	1.000255	1.426353	0.049672	0.000929
TCIM	0.905158	0.819395	0.999897	0.049763	0.017455
ZC3H12A	0.85826	0.736713	0.999861	0.049792	0.005237
IRX6	1.075027	1.000059	1.155615	0.049813	0.039125
